# The mutualism–antagonism continuum in Neotropical palm–frugivore interactions: from interaction outcomes to ecosystem dynamics

**DOI:** 10.1111/brv.12809

**Published:** 2021-11-01

**Authors:** Caroline Marques Dracxler, W. Daniel Kissling

**Affiliations:** ^1^ Institute for Biodiversity and Ecosystem Dynamics (IBED) University of Amsterdam P.O. Box 94240 Amsterdam 1090 GE The Netherlands

**Keywords:** Arecaceae, ecosystem functioning, ectozoochory, endozoochory, granivory, palm endocarp, seed caching, seed dispersal distance, seed predation, vertebrate

## Abstract

Frugivory, that is feeding on fruits, pulp or seeds by animals, is usually considered a mutualism when interactions involve seed dispersal, and an antagonism when it results in the predation and destruction of seeds. Nevertheless, most frugivory interactions involve both benefits and disadvantages for plants, and the net interaction outcomes thus tend to vary along a continuum from mutualism to antagonism. Quantifying outcome variation is challenging and the ecological contribution of frugivorous animals to plant demography thus remains little explored. This is particularly true for interactions in which animals do not ingest entire fruits, that is in seed‐eating and pulp‐eating. Here, we provide a comprehensive review of Neotropical palm–frugivore interactions, with a focus on how frugivore consumption behaviour (i.e. digestive processing, fruit‐handling ability and caching behaviour) and feeding types (fruit‐eating, pulp‐eating and seed‐eating) influence interaction outcomes at different demographic stages of palms. We compiled a total of 1043 species‐level palm–frugivore interaction records that explicitly captured information on which parts of palm fruits are eaten by animals. These records showed consumption of fruits of 106 Neotropical palm species by 273 vertebrate species, especially birds (50%) and mammals (45%), but also fish (3%) and reptiles (2%). Fruit‐eating involved all four taxonomic vertebrate classes whereas seed‐eating and pulp‐eating were only recorded among birds and mammals. Most fruit‐eating interactions (77%) resulted in positive interaction outcomes for plants (e.g. gut‐passed seeds are viable or seeds are successfully dispersed), regardless of the digestive processing type of vertebrate consumers (seed defecation *versus* regurgitation). The majority of pulp‐eating interactions (91%) also resulted in positive interaction outcomes, for instance *via* pulp removal that promoted seed germination or *via* dispersal of intact palm seeds by external transport, especially if animals have a good fruit‐handling ability (e.g. primates, and some parrots). By contrast, seed‐eating interactions mostly resulted in dual outcomes (60%), where interactions had both negative effects on seed survival and positive outcomes through seed caching and external (non‐digestive) seed dispersal. A detailed synthesis of available field studies with qualitative and quantitative information provided evidence that 12 families and 27 species of mammals and birds are predominantly on the mutualistic side of the continuum whereas five mammalian families, six mammal and one reptile species are on the antagonistic side. The synthesis also revealed that most species can act as partial mutualists, even if they are typically considered antagonists. Our review demonstrates how different consumption behaviours and feeding types of vertebrate fruit consumers can influence seed dispersal and regeneration of palms, and thus ultimately affect the structure and functioning of tropical ecosystems. Variation in feeding types of animal consumers will influence ecosystem dynamics *via* effects on plant population dynamics and differences in long‐distance seed dispersal, and may subsequently affect ecosystem functions such as carbon storage. The quantification of intra‐ and inter‐specific variation in outcomes of plant–frugivore interactions – and their positive and negative effects on the seed‐to‐seedling transition of animal‐dispersed plants – should be a key research focus to understand better the mutualism–antagonism continuum and its importance for ecosystem dynamics.

## I. INTRODUCTION

Plant–frugivore interactions are widely considered mutualisms because the consumption of fruits by frugivores mostly results in seed dispersal, a process that is crucial for the establishment of animal‐dispersed plant populations (Jordano, 1987; Wang & Smith, 2002; Schupp, Jordano & Gómez, 2010; Snell *et al.*, 2019). Nevertheless, it is also recognized that frugivores can act as seed predators when directly feeding on seeds or when seeds do not survive the interaction, therefore leading to an antagonistic (i.e. negative) interaction outcome (Janzen, 1971; Correa *et al*., 2015; Mittelman *et al*., 2021). This mutualism–antagonism duality has widely influenced ecological and evolutionary thinking on plant–frugivore interactions and their outcomes, e.g. in classifications of frugivores as seed dispersers (mutualists) *versus* seed predators (antagonists) (Bronstein, 1994; Genrich *et al*., 2017). Such a dual perspective has helped to reinforce that frugivory not only results in obligate mutualistic interactions, but it has also prevented a full understanding of the intra‐specific variability of effects that frugivore behaviours exert on plant population dynamics (e.g. on seed viability, seed deposition and seedling establishment stages) (Hulme, 2002; Simmons *et al*., 2018; Zwolak, 2018; Gómez, Schupp & Jordano, 2019; Schupp *et al*., 2019). Quantifying the role of frugivores along a mutualism–antagonism continuum is therefore of fundamental importance for understanding plant population and ecosystem dynamics.

A mutualism is defined as an interaction between individuals of two species that is beneficial to both partners (Bronstein, 2001). In frugivory, this involves benefits for both the animal and plant species involved in the interaction. For animals, the benefits come from food and energy resources provided by the plants whereas for plants the benefits depend on the effects of frugivores on various demographic stages during the seed‐to‐seedling transition. For instance, frugivores can positively influence seed viability by their direct or indirect effects on seeds (e.g. *via* gut treatment of seeds or seed caching), influencing whether seeds survive interactions or not (Schupp *et al.*, 2010). Moreover, frugivores also determine the sites of seed deposition, resulting in benefits for plants if deposition sites match germination and/or establishment requirements (Côrtes & Uriarte, 2013; Snell *et al*., 2019). At this stage, aspects such as dispersal distances and the quality of deposition microsites, which aggregate biotic conditions (e.g. conspecific and heterospecific seed density, levels of seed predation) and abiotic factors (e.g. luminosity and soil characteristics), determine whether outcomes are positive or negative for the plant (Schupp *et al*., 2010; Lopez‐Toledo *et al*., 2013; Snell *et al*., 2019). These combined effects ultimately determine the influence of frugivory on seedling establishment (Beckman & Rogers, 2013). Importantly, frugivores can exert contrasting effects on different demographic processes, for instance when viable seeds (i.e. after a positive effect on seed viability) are deposited in places that are unsuitable for germination (i.e. *via* a negative effect at the seed‐deposition stage). When interactions with frugivores involve only disadvantages for the plant (e.g. predation of seeds or seed dispersal only to sites which are unsuitable for germination), they are considered antagonistic (Bronstein, 2001). Nevertheless, most plant–frugivore interactions involve both benefits and disadvantages, and interaction outcomes can thus vary along a continuum from mutualism to antagonism (Thompson, 1988; Bronstein, 1994; Montesinos‐Navarro *et al*., 2017; Rodríguez‐Rodríguez, Jordano & Valido, 2017; Gómez *et al*., 2019).

A key element for determining the effects of frugivores on plants is how frugivores interact with fruits and/or seeds (hereafter ‘types of feeding interactions’ or ‘feeding guilds’). For instance, animals can ingest whole fruits (‘fruit‐eaters’), they may only consume the pulp and thus discard the seeds (‘pulp‐eaters’), or they may only feed directly on the seeds and thus discard the pulp (‘seed‐eaters’). These feeding behaviours determine to what extent different plant demographic processes can be affected by frugivores and influence whether interaction outcomes are positive or negative. Interaction outcomes can vary further among different animal species within the same feeding guild. For instance, fruit‐eaters often have a positive influence on seed viability and seed germination due to the gut passage of seeds, but mechanical and chemical effects associated with gut treatment may also decrease seed viability (Traveset, 1998; Traveset & Verdú, 2002). Seed‐eaters mostly have negative effects on seed survival because the seeds are their main food target (Janzen, 1971; Hulme & Benkman, 2002). Nevertheless, some seed‐eaters such as scatter‐hoarding rodents are known to be effective seed dispersers of some plant species because some of the seeds placed in caches can escape retrieval and predation if caches are neglected or abandoned (Vander Wall, 1990; Vander Wall & Beck, 2012; Zwolak & Crone, 2012; Lichti, Steele & Swihart, 2017; Mittelman *et al*., 2021). This might be particularly true for plants that produce large, long‐lasting seeds that are perceived by rodents as good items for food storage. Pulp‐eaters do not necessarily prevent or favour seed dispersal (potentially playing a neutral role), but pulp removal can favour seed escape and increase seed viability and/or seed germination (Loayza & Knight, 2010; Fedriani & Delibes, 2013). In some cases, pulp‐eaters can also damage seeds during pulp consumption, therefore acting as seed predators because of their negative effects on seed viability (Simmons *et al*., 2018). Hence, the position of each frugivore species varies along the mutualism–antagonism continuum, with fruit‐eaters being more likely located towards the mutualism end and seed‐eaters being found more to the antagonistic end. However, interaction outcomes can even differ for the same animal species (Zwolak, 2018; Schupp *et al*., 2019) when feeding on different plant species or different parts of the fruits. This makes the quantification of ecological roles of frugivores a challenging task.

**Fig. 1:**
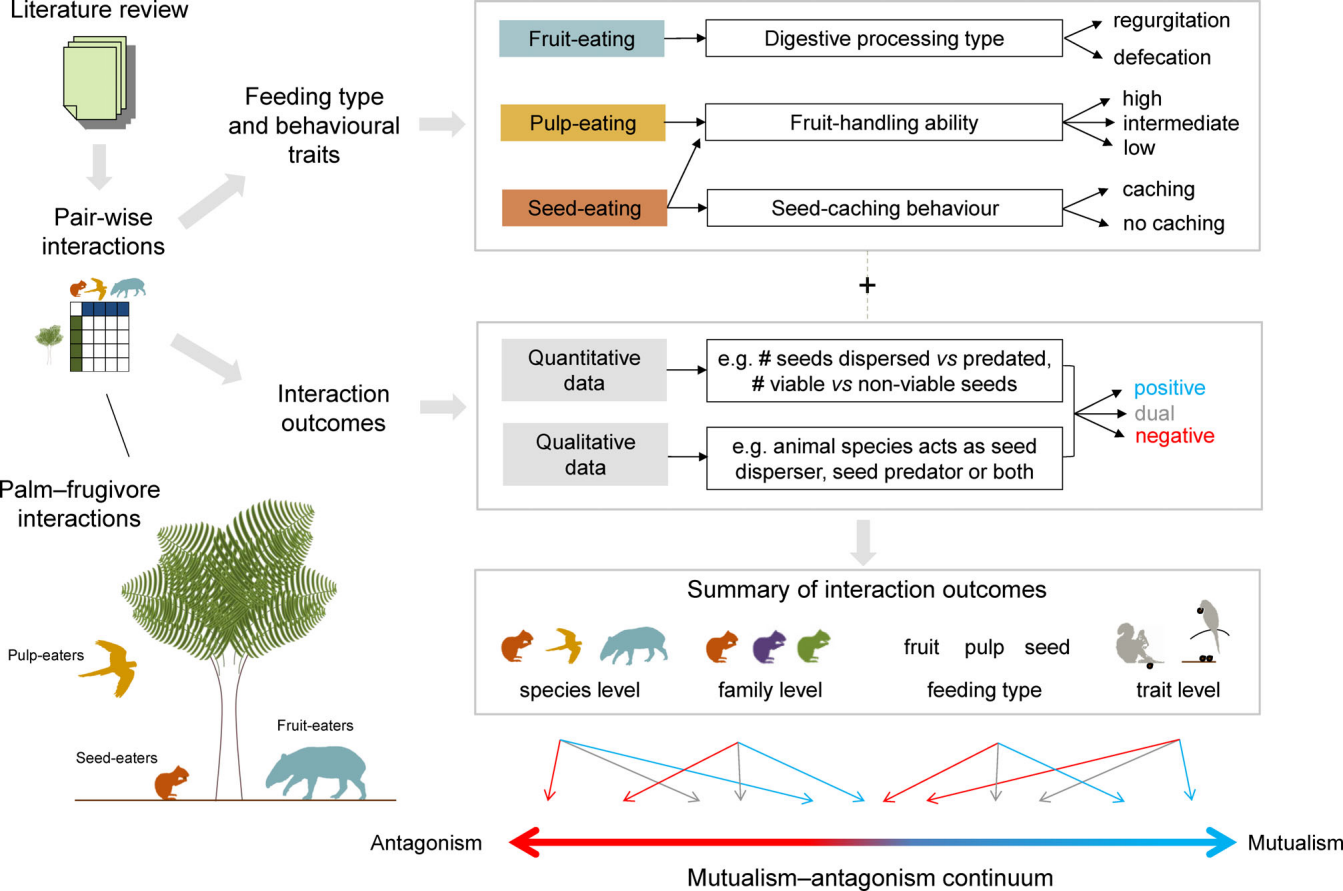
Methodological framework for synthesizing interaction outcomes in Neotropical palm–frugivore interactions along a mutualism–antagonism continuum. From a comprehensive literature review, pairwise interaction data between palms and vertebrate fruit, pulp and seed consumers were identified. Qualitative and quantitative information on feeding types, animal traits and interaction outcomes was extracted. Interaction outcomes were summarized along a mutualism–antagonism continuum at the level of species, families, feeding types and traits.

Here, we review the outcomes of plant–frugivore interactions in Neotropical palms (Arecaceae) to assess our current knowledge of the ecological effects of frugivores on the seed‐to‐seedling transition and their placement along the mutualism–antagonism continuum. Palms are particularly abundant in the Neotropics (ter Steege *et al*., 2013; Muscarella *et al*., 2020) and this biogeographic realm also contains a tremendous diversity of frugivores (Howe & Smallwood, 1982; Fleming, Breitwisch & Whitesides, 1987; Kissling, Böhning‐Gaese & Jetz, 2009). We focus on palms because they are an excellent model system to understand the ecology and evolution of tropical rainforests and their biota (Henderson, 2002; Eiserhardt *et al*., 2011; Couvreur & Baker, 2013; Onstein *et al*., 2017), and because palms provide key food resources for many frugivores, including mammals, birds, reptiles, beetles, crabs, and fish (Zona & Henderson, 1989; Andreazzi, Pires & Fernandez, 2009; Muñoz, Trojelsgaard & Kissling, 2019). They are also a key resource for people and an important part of the livelihoods of rural populations, providing important ecosystem services (Cámara‐Leret *et al*., 2017). With approximately 2,500 palm species distributed globally, and about 700 species in the Neotropics (Kissling *et al*., 2012), fruits and seeds of palms represent a large part of the diet of many animal species, and exhibit fruit traits that are clearly attractive for animals that feed on fruits, pulp and seeds (Kissling *et al*., 2019). We conducted a comprehensive literature review with the aim to identify different types of feeding interactions (i.e. fruit‐eating, pulp‐eating and seed‐eating) and the influence of consumption behaviour (digestive processing, caching behaviour and fruit‐handling ability) on outcomes of Neotropical palm–frugivore interactions at different palm demographic stages (Fig. 1). We gathered information on animal traits such as the digestive processing by fruit‐eaters (i.e. regurgitation or defecation of seeds), seed caching behaviour by seed‐eaters and fruit‐handling ability of pulp‐ and seed‐eaters. This allowed us to quantify how intra‐ and inter‐specific variation of frugivory affects interaction outcomes. Specifically, we focus on the effects of frugivory on three key demographic processes of the seed‐to‐seedling transition of plants, namely seed viability, seed deposition and seedling establishment. Effects of frugivores on each of these three demographic processes can vary both intra‐ and inter‐specifically, resulting in variation along the mutualism–antagonism continuum. We finally discuss important mechanisms of fruit‐, pulp‐ and seed‐eating, how they influence interaction outcomes and ultimately ecosystem processes such as seed dispersal and seedling establishment, and which implications these interactions have for ecosystem dynamics such as carbon storage and nutrient cycling.

## II. METHODS

### (1) Compilation of interaction records and types of feeding interactions

We compiled interaction records of animals feeding on Neotropical palm fruits by screening articles from a comprehensive literature search on the *Web of Science* (WoS) conducted from October 2019 to July 2021. The Neotropical realm comprises South America, Central America, parts of Mesoamerica (Yucatan Peninsula), the Caribbean, and the southern parts of Texas and Florida in the USA (Udvardy, 1975). We used a combination of search terms that included ‘fruit removal’, ‘seed dispersal’, ‘seed predation’, ‘frugivory’, ‘granivory’, ‘seed’, ‘fruit’, ‘palm’, ‘Arecaceae’, ‘Neotropics’ and all Neotropical palm genera and country names (see online Supporting Information, Appendix S1, for the precise combination of search terms). We also conducted searches on the WoS using the same search terms translated into French, Portuguese and Spanish, since these languages are also used for publications in peer‐reviewed journals in the Neotropics. This combination of terms allowed us to identify studies that included information on frugivore species feeding on the fruits and seeds of palm species in the entire Neotropics.

We obtained an initial list of 590 articles. All articles were manually and meticulously screened for information on interactions between frugivores and palm fruits. We also occasionally added additional literature that was cited in the screened articles. We extracted pairwise interaction records from an article when explicit information on animal species feeding on fruits, pulp and seeds of palm species was provided. This was most often referred to as frugivory, granivory, scatter‐hoarding, fruit removal, seed dispersal, seed predation, fruit consumption, seed consumption or pulp consumption, but pairwise interactions could also be extracted when articles provided information on seed defecation, regurgitation, seed hoarding or seed caching. We compiled pairwise interactions regardless of how the interaction outcomes were classified or which type of feeding the animals were displaying. We did not include interaction records when specific species involved in pairwise interactions were not clearly identified taxonomically, when evidence was only anecdotal, or when animal identity was only assumed by the authors (e.g. studies assessing fruit removal with no visual evidence of which animal species had actually removed the fruits). A total of 205 articles contained species‐level information on pairwise interactions between animals and palm fruits, pulp and seeds.

Beyond simply recording the interaction between a palm and a frugivore, we aimed to identify the type of feeding in Neotropical palm–frugivore interactions (Fig. 1). We distinguished three different feeding interaction types: (*i*) fruit‐eating (animals ingesting entire fruits), (*ii*) pulp‐eating (animals feeding on fruit mesocarps only), and (*iii*) seed‐eating (animals discarding fruit mesocarps and feeding only on seeds). This classification was done at the level of each individual interaction record to capture intra‐specific variability because feeding behaviour of the same frugivore species may vary for different palm species. All species‐level interaction records that specifically contained information on types of feeding interactions were considered eligible and included in this review (the data set is available from Dracxler & Kissling, 2021).

### (2) Classification of interaction outcomes

We further classified the outcome of each interaction record (where possible) as (*i*) positive, (*ii*) negative or (*iii*) dual for all three feeding interaction types (Fig. 1). Evidence on interaction outcomes came from both quantitative and qualitative information in the screened articles, e.g. field observations or experimental quantification of seed dispersal, seed caching, seed predation, viability and germination of gut‐passed or handled seeds, or seedling establishment (see criteria in Appendix S2). Available quantitative data on interaction outcomes included the number of seeds dispersed *versus* predated, viable *versus* non‐viable gut‐passed seeds, and success of seed germination or seedling establishment. From this information, we calculated the proportion of positive events (proportion of seeds dispersed, proportion of viable seeds after gut passage, proportion of seeds germinated, etc.) and classified interaction outcomes as positive if the proportion of positive events was ≥50%, and as negative if <50%. Since quantitative evidence is relatively scarce, we additionally extracted qualitative evidence on interaction outcomes. This reflected explicit mentioning in the articles that, for instance, an animal species acted as seed disperser (positive outcome), seed predator (negative) or both (dual). For dual interaction outcomes, seeds could be both predated and dispersed, without knowing the exact fate of the seeds.

### (3) Consumption behaviour

We also compiled information on consumption‐related behavioural traits of frugivores for all three feeding interaction types (fruit‐, pulp‐ and seed‐eating) because this can influence interaction outcomes (Simmons *et al*., 2018) (Fig. 1). For fruit‐eating, we differentiated for each interaction record (whenever possible) how animals expel seeds (i.e. by seed regurgitation, defecation, or both) (Dracxler & Kissling, 2021) because this relates to the gut treatment of seeds which can influence, for instance, seed viability, the quality of seed deposition sites and dispersal distances (Schupp *et al*., 2010). For seed‐eating, we distinguished whether seed‐eaters show caching behaviour or not to evaluate if mechanisms related to seed caching (e.g. seed cleaning, dispersal and burial) influence interaction outcomes. Information on presence of seed‐caching behaviour was compiled for each animal species involved in at least one seed‐eating interaction (Dracxler & Kissling, 2021), either from articles containing the interaction records or from additional literature sources that described the behaviour of the animal at the species level. For seed‐eating and pulp‐eating, we further classified the animals' fruit‐handling ability into low, intermediate, or high (Fig. 1). Differences in fruit‐handling ability can affect feeding efficiency and consumption rates, as well as dispersal and interaction outcomes (Levey, 1987; Feer *et al*., 2001; de Araújo & Marcondes‐Machado, 2011; Fuzessy, Janson & Silveira, 2018). Examples of differences in fruit‐handling ability are the direct consumption of pulp or seeds by tanagers (low fruit‐handling ability), the holding of fruits or seeds by macaws with their feet (intermediate fruit‐handling ability), and the use of forepaws and sitting behaviour to manipulate fruits and seeds by scatter‐hoarding rodents such as agoutis or primates (high fruit‐handling ability). Such information was also compiled at the animal species level (Dracxler & Kissling, 2021).

### (4) Mutualism–antagonism continuum

To assess interaction outcomes along the mutualism–antagonism continuum, we aggregated outcomes (positive or negative) either at the species level (summarizing intra‐specific variability) or at the family level (summarizing inter‐specific variability) [see Gómez *et al*., (2019) for a variation of the continuum approach]. If the aggregated proportion of positive outcomes was ≥0.5, the frugivore species or family was considered to be mostly mutualistic, and if <0.5, then mostly antagonistic. We placed taxa along the mutualism–antagonism continuum using either quantitative or qualitative data on interaction outcomes extracted from the screened articles (see Section II.2). Qualitative data could be assessed at both the species and the family level whereas quantitative data were only available at the species level. We used a minimum of seven studies with qualitative outcomes to aggregate information at the family level along the continuum, and we only included families represented by at least two species (to show inter‐specific variation of outcomes within families). For species‐level continuums, we summarized outcomes for species with at least five outcome records. Species‐level qualitative data on outcomes were abundant, but quantitative data could be summarized for only three species of frugivores for which ≥5 interaction records reported in the screened articles provided evidence on interaction outcomes from field observations or experiments.

## III. EXTENT OF THE LITERATURE REVIEW

**Fig. 2:**
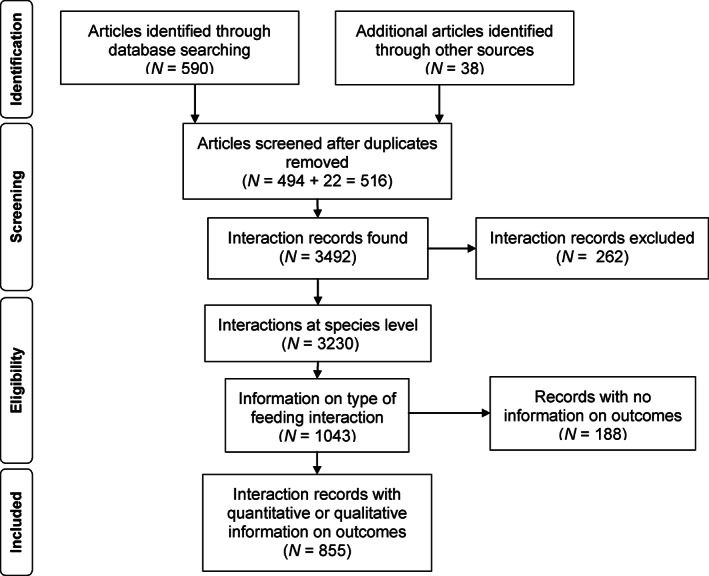
Flow diagram depicting the identification of articles, their manual screening, and reasons for eligibility of interaction records and their inclusion or exclusion. The structure of the flow diagram follows the PRISMA statement for transparent reporting of systematic reviews (Moher *et al*., 2009).

Article screening allowed for the compilation of 3492 interaction records, from which 3230 described evidence of an animal species feeding on a particular palm species. A total of 1043 species‐level records (32% of all records) recorded in 168 articles contained information on which parts of fruits animals feed upon (e.g. type of feeding interaction) (Dracxler & Kissling, 2021) and were therefore eligible for the classification of interaction outcomes and for inclusion in our review (Fig. 2). Interaction records with information on parts of fruits that animals consume were available from Argentina, Belize, Bolivia, Brazil, Colombia, Costa Rica, Ecuador, French Guiana, Guatemala, Honduras, Mexico, Panama, Peru, Trinidad and Tobago, Venezuela and the USA. Pairwise interaction records were obtained either from reviews (e.g. Beck, 2006; Gómez *et al*., 2019; Mendes, Koprowski & Galetti, 2019) or from original articles. Quantitative and/or qualitative information on outcomes available in the screened studies allowed us to classify interaction outcomes for 855 records (82%) with information on feeding interaction types (Fig. 2; Table 1) (Dracxler & Kissling, 2021).

**Table 1:** Total number of interaction records for each interaction type (fruit‐eating, pulp‐eating and seed‐eating) and the number of interactions with classified outcomes. Number of records resulting in positive, negative or dual outcomes as well as the percentage of outcomes per feeding types are shown.

	Number of interactions		Interaction outcomes					
Type of feeding interaction	Total	With outcomes	Positive	%	Negative	%	Dual	%
Fruit‐eating	545	398	308	77%	66	17%	24	6%
Pulp‐eating	115	87	79	91%	5	6%	3	3%
Seed‐eating	383	370	41	11%	106	29%	222	60%
Total	1043	855	428	50%	177	21%	249	29%

## IV. TYPES OF FEEDING INTERACTIONS AND THEIR TAXONOMIC COMPOSITION

About half of the records with information on the type of feeding interaction involved fruit‐eating (52%), followed by seed‐eating (37%) and pulp‐eating (11%) (Dracxler & Kissling, 2021). These records included a total of 273 animal species recorded to feed on the fruits, pulp or seeds of 106 palm species (Table 2) belonging to 13 palm tribes and 40 palm genera (Appendix S3; Dracxler & Kissling, 2021). The majority of animal species recorded were birds and mammals (Table 2). Both groups also showed the largest amount of (total and unique) interaction records, with the number of interaction records being larger for mammals than for birds (Table 2). The diversity of animal families represented in records with data on feeding interaction types was largest in birds (*N* = 30 families), followed by mammals (*N* = 28), fish (*N* = 5) and reptiles (*N* = 5), while the diversity of animal genera was higher in mammals (*N* = 87 genera), followed by birds (*N* = 76), fish (*N* = 9) and reptiles (*N* = 5) (Dracxler & Kissling, 2021).

**Table 2:** Number of frugivore species in each animal class and the corresponding number of palm species they were recorded to feed on. *Number of unique palm species

	Number of species		Interactions	
Frugivore class	Frugivores	Palms	Unique	Total
Aves	136	38	194	310
Osteichthyes	10	4	12	15
Mammalia	122	93	383	708
Reptilia	5	10	10	10
Total	273	106*	599	1043

**Fig. 3:**
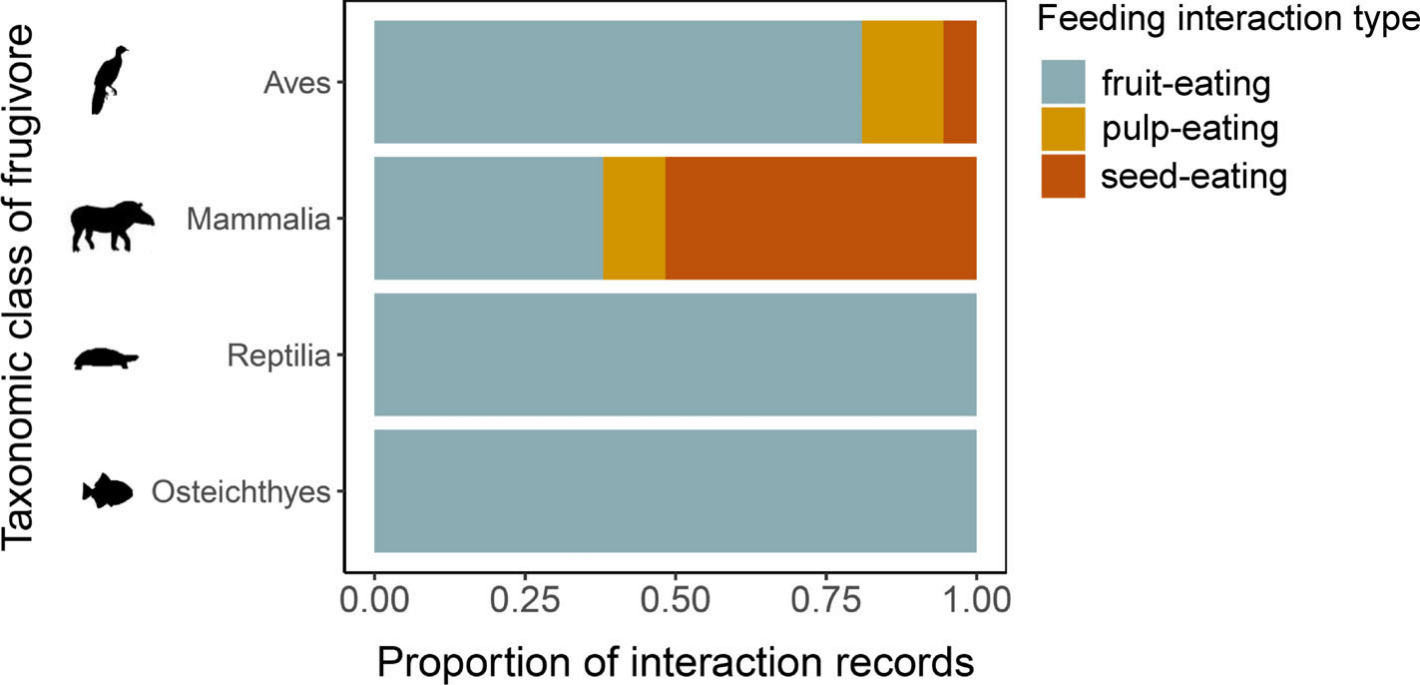
Frequency of types of feeding interactions for four taxonomic classes of frugivores (birds, mammals, reptiles and fish). Proportions of interactions are calculated based on the number of interaction records for which feeding interaction types could be identified (birds: *N* = 310; mammals: *N* = 708; reptiles: *N* = 10; fish: *N* = 15). Feeding interaction types: fruit‐eating = ingesting entire fruits; pulp‐eating = feeding on fruit mesocarps only; seed‐eating = feeding only on seeds (discarding fruit mesocarp).

Birds acted mostly as fruit‐eaters, but some families such as the parrots (Psittacidae) are recorded to feed mainly on pulp or seeds of palm fruits (Appendix S4). All records involving reptile and fish species suggested the ingestion of entire palm fruits (Fig. 3). By contrast, mammals predominantly fed on seeds, although consumption of entire fruits was also high (Fig. 3). Only birds and mammals acted as pulp‐eaters (Fig. 3).

### (1) Fruit‐eaters

A total of 189 frugivore species were recorded in fruit‐eating interactions with 80 palms, totalling 545 interaction records. The majority (58%) of fruit‐eaters were birds, including toucans (Ramphastidae), parrots (Psittacidae), chachalacas, guans and curassows (Cracidae) and thrushes (Turdidae), which accounted for around 40% of all fruit‐eating bird species observed ingesting entire palm fruits (Appendix S4). Mammals such as squirrels (Sciuridae), spiny rats (Cricetidae), primates (Atelidae and Cebidae) and carnivores (Canidae) accounted for 45% of all mammal species ingesting entire palm fruits, but ungulates (Tapiridae, Tayassuidae) and other carnivores (Procyonidae) were also important fruit‐eaters (Appendix S4). The small number of fish and reptile species were also reported to feed on entire palm fruits (Appendix S4).

### (2) Pulp‐eaters

Pulp‐eaters were represented by 32 bird species and 32 mammal species (Appendix S4), which were recorded to feed on the pulp of 27 palm species with a total of 115 interaction records. The two families with the highest number of pulp‐eater species were bird families, namely tanagers (Thraupidae) and parrots (Psittacidae). The other pulp‐feeding birds belonged to eight other families (Appendix S4). Among mammals, primates, marsupials and rodents were among the main mammalian pulp‐feeders of palms, including monkeys (Cebidae, Atelidae), opossums (Didelphidae), squirrels (Sciuridae), agoutis and acouchis (Dasyproctidae), and small rodents (Cricetidae). Each of these families was represented by 3–6 species (Appendix S4).

### (3) Seed‐eaters

A total of 73 animal species were recorded to feed on the seeds of 63 palm species (Appendix S4), with a total of 383 interaction records. This type of feeding interaction was largely dominated by rodent species (*N* = 62 spp.), which were involved in almost 95% of all seed‐eating interaction records (Appendix S4). Other mammalian seed‐eaters included primates and ungulates (Appendix S4). All five bird species involved in seed‐eating interactions with palm seeds were parrots and macaws (Psittacidae) (Appendix S4).

## V. INTERACTION OUTCOMES IN PALM–FRUGIVORE INTERACTIONS

**Fig. 4:**
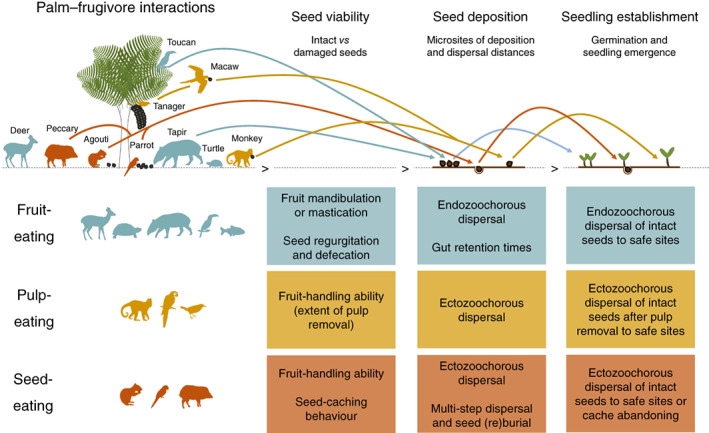
Examples of how the consumption behaviour involved in different feeding interaction types (fruit‐, pulp‐ and seed‐eating) can affect interaction outcomes during the seed‐to‐seedling transition of plants (e.g. seed viability, seed deposition, and seedling establishment). The examples are not exhaustive and refer to consumption behaviours associated with the digestive processing of fruits by fruit‐eaters, the handling ability of pulp‐ and seed‐eaters, and the seed‐caching behaviour of seed‐eaters, as reported in studies of palms (Arecaceae). Silhouettes representing animal taxa exemplify core groups of animal consumers of palm fruits, pulp and seeds. Palm–frugivore interactions are not restricted to these taxa.

We classified interaction outcomes as positive, negative, or dual for 73% of the available fruit‐eating records, 76% of the pulp‐eating records, and 97% of the seed‐eating records (Table 1). The amount and frequency of interaction outcomes varied according to the feeding interaction type (Table 1), with positive outcomes being the most prevalent among fruit‐ and pulp‐eaters and dual outcomes being the most prevalent outcome among seed‐eaters. The interaction outcomes reflected a variety of effects on different stages during the seed‐to‐seedling transition of palms, including seed viability, seed deposition, and seedling establishment (Fig. 4). In the following, we synthesise for (*i*) fruit‐eating, (*ii*) pulp‐eating and (*iii*) seed‐eating how interaction outcomes vary taxonomically and how they may influence plant demographic processes during the seed‐to‐seedling transition *via* effects on seed viability, seed deposition, and seedling establishment (Fig. 4). This includes, for instance, positive and negative effects of (*i*) fruit mastication, gut treatment, and endozoochorous seed dispersal of fruit‐eaters, (*ii*) pulp removal and ectozoochorous seed dispersal of pulp‐eaters, and (*iii*) ectozoochorous seed dispersal and seed caching of seed‐eaters (Fig. 4).

### (1) Fruit‐eating

**Fig. 5:**
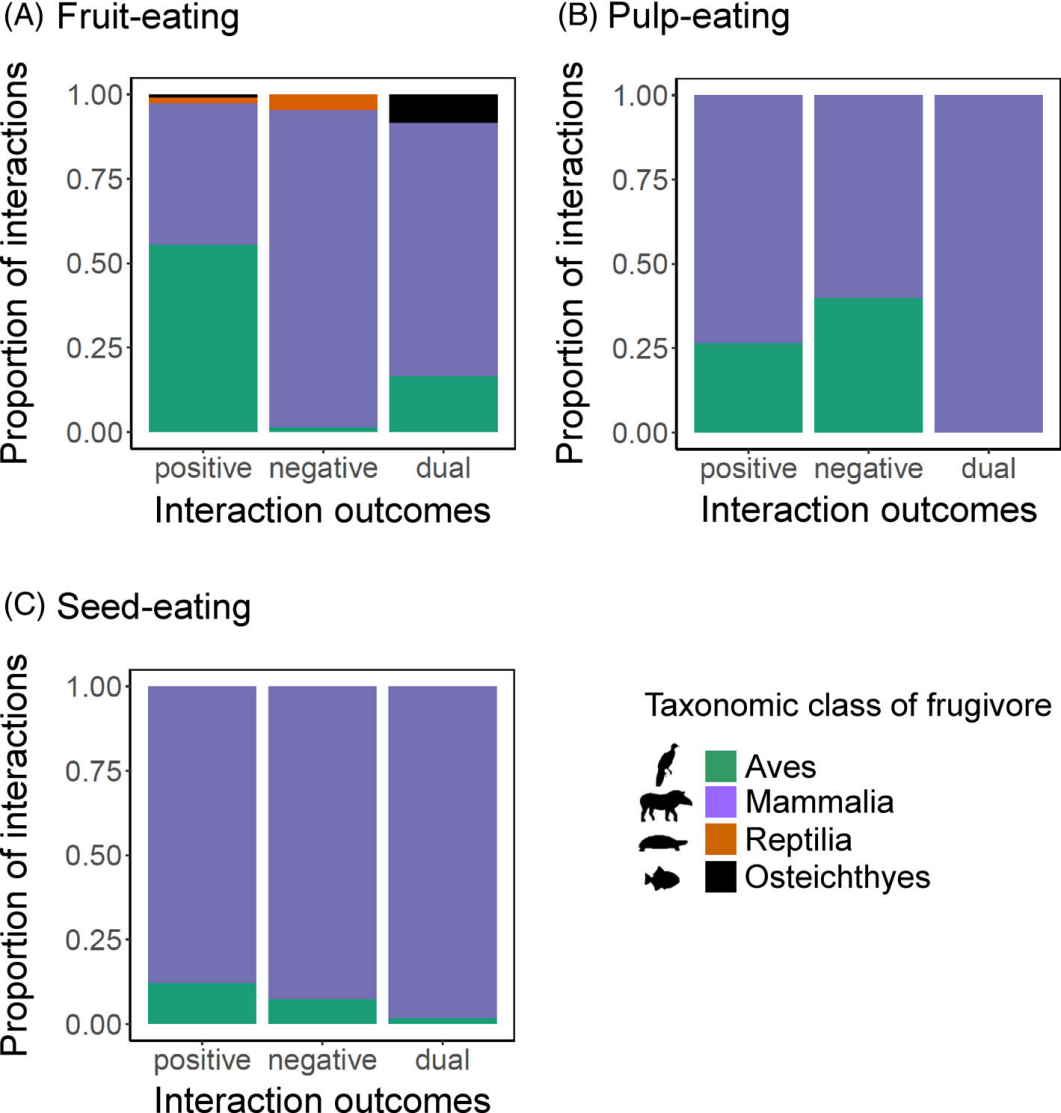
Proportion of interaction records that describe positive, negative or dual interaction outcomes for different taxonomic classes of animals (birds, mammals, reptiles, fish), per feeding interaction type. (A) Fruit‐eating (*N* = 398 interaction records), (B) pulp‐eating (*N* = 87 interaction records), and (C) seed‐eating (*N* = 370 interaction records).

**Fig. 6:**
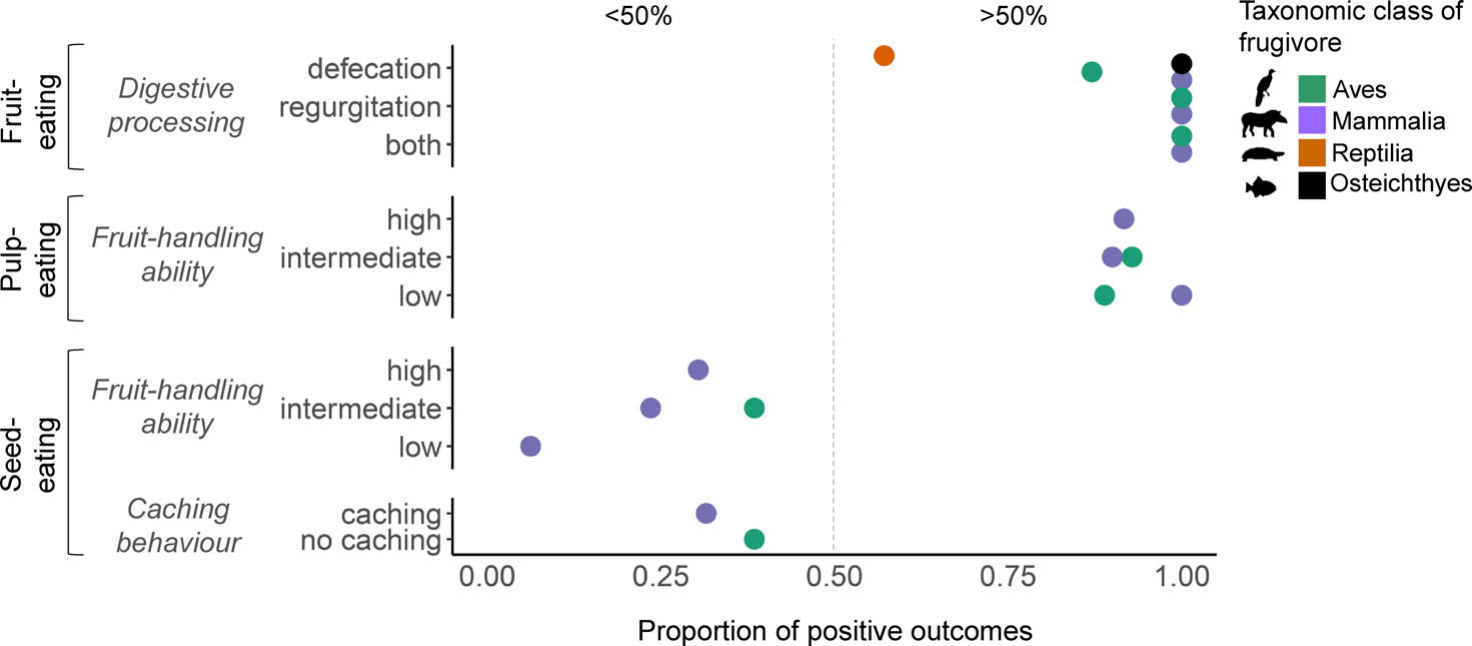
Variation in interaction outcomes within and across different feeding guilds (fruit‐, pulp‐ and seed‐eating). The proportion of positive interaction outcomes (*x*‐axis) is shown for different behavioural traits and separated by taxonomic class of frugivores. Behavioural traits are related to the digestive‐processing type (defecation, regurgitation, both) of fruit‐eaters, the fruit‐handling ability (high, intermediate, low) of pulp‐eaters and seed‐eaters, and the seed‐caching behaviour (caching, no caching) of seed‐eaters. The right side of the continuum (>50%) shows mostly mutualistic outcomes and the left side (<50%) mainly antagonistic outcomes.

A total of 398 fruit‐eating interaction records had information on interaction outcomes, capturing 251 unique interactions between 125 frugivore and 57 palm species. Almost 80% of the fruit‐eating interaction records resulted in positive outcomes (Table 1), mainly involving birds and mammals (Fig. 5A). Negative and dual outcomes of fruit‐eating interaction records were dominated by mammals (Fig. 5A). The type of digestive processing of fruits by animals (defecation, regurgitation, or both) was identified for 211 interaction records, involving 75 animal species and 45 palms (Dracxler & Kissling, 2021). This mainly involved the expelling of seeds by defecation (*N* = 141, mostly mammals) and to a lesser extent regurgitation (*N* = 53, mostly birds), and sometimes both (*N* = 17, mostly birds) (Appendix S5). Most cases of fruit‐eating for which the digestive process could be identified resulted in positive outcomes, regardless of how seeds were expelled (Fig. 6; Appendix S5).

#### (a) Fruit treatment in the beak or mouth

Many fruit‐eaters ingest palm fruits without damaging the seeds, in part because palm endocarps offer a physical protection to seeds against mechanical and chemical damage, but also because many animal species do not mandibulate or masticate fruits in the mouth or beak, swallowing fruits whole. Fruit‐eating without fruit processing in the oral cavity is seen, for example, in gulpers, that is birds that ingest whole fruits without processing in the bill (Levey, 1987). Toucans and toucanets (Ramphastidae) are good examples of gulpers and also important consumers of palm fruits, feeding on entire fruits of at least 11 palm species in interactions that are reported to result in positive outcomes. However, fruit‐eaters can damage palm seeds in their mouth or beak when mandibulating or masticating fruits before swallowing them. The small number of negative outcomes of fruit‐eating reported here are caused by seed damage derived from fruit mastication mainly by turtles (Chelidae), peccaries (Tayassuidae), tapirs (Tapiridae) (Zorzi, 2009; Hibert *et al*., 2011) deer (Cervidae) and agoutis (Dasyproctidae) (Beck, 2006; Bodmer & Ward, 2006; Caputo & Vogt, 2008), but fruit‐eating by fish and birds can also involve fruit mastication and lead to seed predation (Moegenburg & Levey, 2003; Piedade, Parolin & Junk, 2006; Correa *et al*., 2007). Such damage during mastication is often caused when palm seeds have a relatively thin and fragile endocarp, such as those of *Euterpe* palms. However, fruit processing in the mouth or beak can also lead to damage of palm seeds that have stony endocarps, such as those in the palm genera *Astrocaryum* and *Attalea* (Beck, 2006; Bodmer & Ward, 2006). Negative effects on seed viability due to mastication are further caused by the consumption of immature fruits, as reported for carnivores (Kays, 1999), rodents (Smythe, 1989; Mendes & Cândido‐Jr, 2014; Acevedo‐Quintero, Zamora‐Abrego & Ortega‐León, 2018) and primates (Smythe, 1989; Bowler & Bodmer, 2011).

#### (b) Gut treatment

After fruits are swallowed, they pass through the gut, and fruit‐eaters either defecate or regurgitate the seeds. This may influence seed viability because fruit processing in the gut involves a series of chemical and mechanical actions that can affect the structure of the seed coat. This influence can be positive when fruit processing in the gut favours germination, for example due to the breakage of seed dormancy and facilitation of germination, or negative when seeds are destroyed as a result of harsh or long gut treatment (Torres, Castaño & Carranza‐Quiceno, 2020). Whether seeds benefit from gut treatment or not also depends on specific traits of seeds such as seed size and hardness (Verdú & Traveset, 2004; Fuzessy *et al*., 2016; Fricke *et al*., 2019). Examples of regurgitation of viable palm seeds after fruit‐eating (i.e. positive effects of gut treatment) include interactions between palm species and bird species of families such as Ramphastidae, Cotingidae and Cracidae (Pizo & Simão, 2001; Moegenburg & Levey, 2003; Sezen, Chazdon & Holsinger, 2009; Karubian *et al*., 2010, 2016; Campos, Steiner & Zillikens, 2012; Ottewell *et al*., 2018). These regurgitated palm seeds have often a high germination success. Viable palm seeds have also been reported inside stomachs or in the dung of ungulates (e.g. Beck, 2006; Bodmer & Ward, 2006; Barcelos *et al*., 2013), carnivores (Kays, 1999; Campos *et al*., 2012), birds (Pizo & Simão, 2001; Cárdenas, Echeverry‐Galvis & Stevenson, 2021), primates (Stevenson & Link, 2010; Chaves, Stoner & Arroyo‐Rodríguez, 2012), turtles (Liu, Platt & Borg, 2004; Caputo & Vogt, 2008) and fish (Sório, Damasceno‐Junior & Parolin, 2015; Barbosa & Montag, 2017). Our review shows that seeds of more than 30 palm species have been reported to benefit from gut treatment that involves seed defecation. In general, most palm seeds involved in fruit‐eating seem to benefit from fruit processing in the gut of animals, regardless of whether they are defecated or regurgitated (Fig. 6; Appendix S5). Previous reviews have also shown that gut treatment of seeds has an overall positive effect on seed viability (Traveset & Verdú, 2002; Fuzessy *et al*., 2016; Torres *et al*., 2020), independent of whether seeds only pass through part of the animal gut (when regurgitated) or through the whole digestive system (when defecated). The results show that Neotropical palm seeds can have high levels of seed protection and thus are well adapted to fruit processing by fruit‐eaters. Nevertheless, more studies addressing the effects of gut treatment on the viability of palm seeds are needed, especially for some understudied fruit‐eaters such as fish and reptiles [but see Correa *et al*. (2015) and Falcón, Moll & Hansen (2020) for reviews of frugivory by these animal groups].

#### (c) Long‐distance seed dispersal by endozoochory

**Fig. 7:**
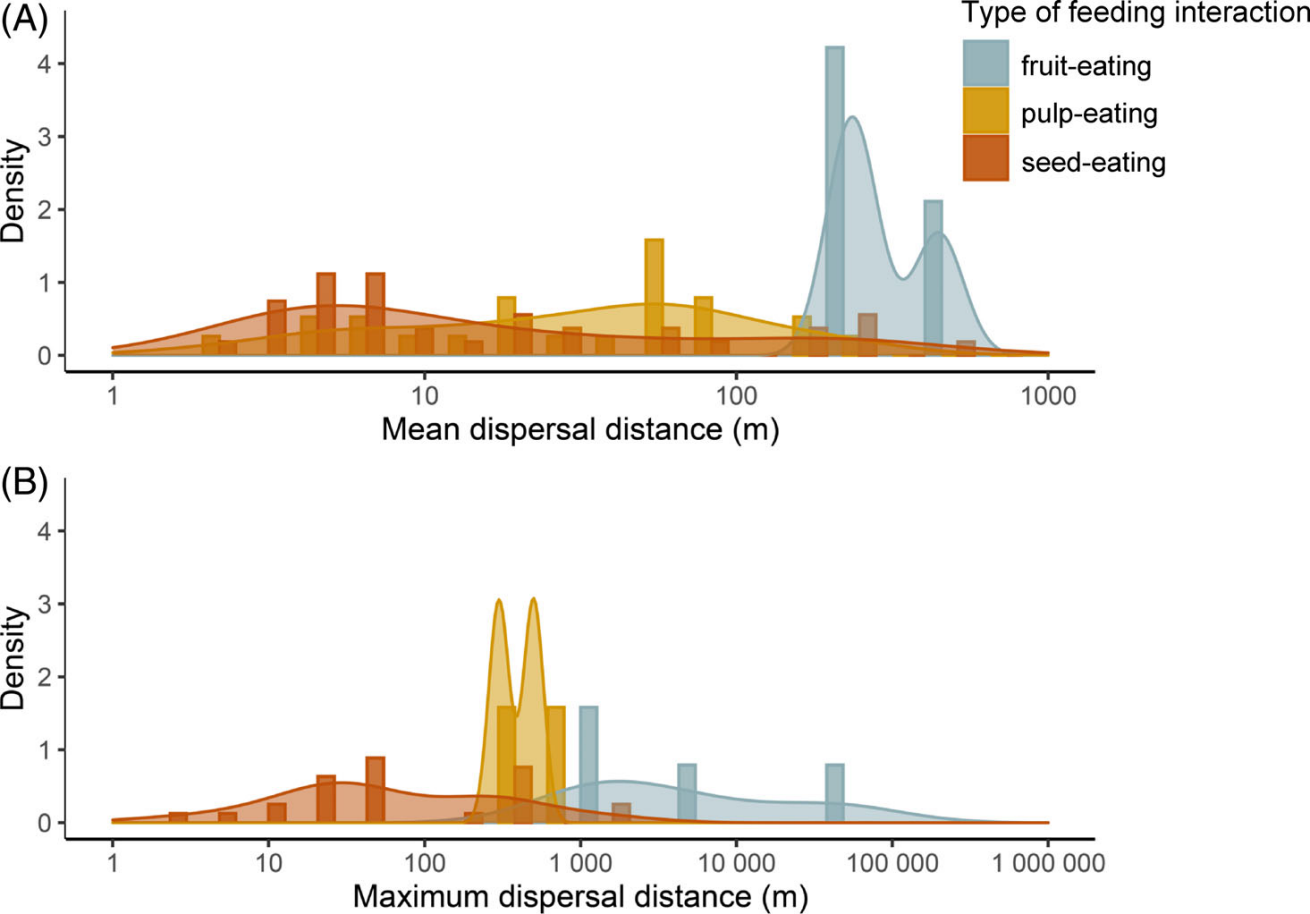
Dispersal distances of fruit‐eaters, pulp‐eaters and seed‐eaters involved in in Neotropical palm–frugivore interactions. (A) Distribution of mean dispersal distances (logarithmic axis) for fruit‐ (*N* = 3 records), pulp‐ (*N* = 25 records) and seed‐eating (*N* = 34 records), involving a total of 26 frugivore species and 22 palm species. (B) Distribution of maximum dispersal distances (logarithmic axis) recorded for fruit‐ (*N* = 4 records), pulp‐ (*N* = 2 records) and seed‐eating (*N* = 25 records), involving a total of 13 frugivore species and 18 palm species. Data on dispersal distances compiled from published articles (see Appendix S6).

Fruit‐eating always involves endozoochory (i.e. seed dispersal *via* ingestion). Subsequent dispersal distances can vary widely and depend on the mobility, space use, body size, gut retention time and digestive fruit‐processing type of seed dispersers (Traveset & Verdú, 2002; Côrtes & Uriarte, 2013). When fruit‐eaters move far after fruit consumption, long‐distance dispersal events are facilitated, often with distances >100 m or even >1 km (Fig. 7; Appendix S6). This contributes to increasing the genetic diversity of palm populations (Sezen *et al*., 2009; Browne, Ottewell & Karubian, 2015; Browne & Karubian, 2018*a*). Long‐distance seed dispersal by frugivores that eliminate viable palm seeds has been suggested for carnivores (Gatti *et al*., 2006; Silva *et al*., 2011; Quintela, Iob & Artioli, 2014; Menezes *et al*., 2018), fish (Anderson *et al*., 2011) and primates (Link & De Luna, 2004; Link & Di Fiore, 2006; Canale *et al*., 2016). Large bird species (e.g. Cotingidae, Cracidae, Ramphastidae, Steathornidae) can deposit seeds far away from parent palms (Appendix S6), showing that avian fruit‐eaters can have an ecological function similar to that of large‐bodied mammalian fruit‐eaters (Stevenson *et al*., 2021). For instance, long‐wattled umbrellabirds (*Cephalopterus penduliger*) show high gut retention times (up to 105 min for seeds of the palm *Oenocarpus bataua*; Karubian *et al*., 2012) and have been shown to disperse intact seeds effectively (Karubian *et al*., 2010) over hundreds of meters away from fruit sources (Karubian *et al*., 2012, 2016). In rare cases like the oilbird (*Steatornis caripensis*), seed dispersal (e.g. of the palm *Oenocarpus bataua*) can even exceed 40 km (Stevenson *et al*., 2021).

#### (d) Seed deposition and seedling establishment

Interaction outcomes of fruit‐eating are influenced by the microsites at which seeds are deposited. Seed deposition sites can be highly variable even when interactions involve the same frugivore species. For instance, latrines of the lowland tapir (*Tapirus terrestris*) are often located at upland forest sites, which are considered suitable sites for the germination of *Attalea maripa* seeds, but tapirs can also use latrines located in flooded areas, which can be disadvantageous for seed germination (Fragoso & Huffman, 2000; Quiroga‐Castro & Roldán, 2001). Such intra‐specific variability in the influence of fruit‐eaters on the deposition of palm seeds can reduce the proportion of positive outcomes derived by palms from interactions with a specific fruit‐eater. By contrast, germination and seedling establishment of palms is facilitated when fruit‐eating animal species consistently deposit seeds at particular microsites with optimal conditions (i.e. directed seed dispersal, such as dispersal to a certain habitat type, at preferred feeding trees, or specific breeding or sleeping sites) (Karubian *et al*., 2012, 2015; Canale *et al*., 2016). For example, long‐wattled umbrellabirds (*Cephalopterus penduliger*) disperse a high proportion of the seeds of *Oenocarpus bataua* within breeding sites known as leks (Karubian *et al*., 2012), many of which are found to germinate and establish into seedlings (Karubian *et al*., 2016). However, this is not the case when palm seeds are dispersed to non‐suitable sites, such as seeds that are deposited in caves by the oilbird *Steatornis caripensis* (Stevenson *et al*., 2017).

When viable palm seeds are deposited at sites that are suitable for germination, fruit‐eaters facilitate the establishment of seedlings. Such positive associations between fruit‐eating and seedling establishment of palms have been found for species of birds (Pizo & Simão, 2001; Sezen *et al*., 2009; Karubian *et al*., 2012), mammals (Fragoso, 1997; Fragoso & Huffman, 2000; Sica, Bravo & Giombini, 2014) and fish (Barbosa & Montag, 2017), involving palm species such as *Attalea maripa*, *Euterpe edulis*, *Iriartea deltoidea*, *Oenocarpus bataua* and *Syagrus romanzoffiana*. For instance, Fragoso, Silvius & Correa (2003) showed that the aggregation of viable *Attalea maripa* seeds in tapir latrines favours local seedling establishment and contributes to generating patches of *A. maripa* adult individuals, an association that was also observed for the Atlantic Forest palm *Syagrus romanzoffiana* (Sica *et al*., 2014).

### (2) Pulp‐eating

For pulp‐eaters, interaction outcomes could be identified for 87 out of 115 interaction records, including 29 mammal species and 15 bird species recorded to interact with 21 and 11 palm species, respectively. Pulp‐eating records resulted mostly in positive interaction outcomes (Table 1).

Most mammalian species recorded in pulp‐eating interactions with classified outcomes have a high fruit‐handling ability (*N* = 15 spp.), but some also showed an intermediate (*N* = 7 spp.) or low (*N* = 7 spp.) ability to handle fruits while consuming the pulp (Dracxler & Kissling, 2021) (Appendix S7A). By contrast, bird species involved in pulp‐eating showed low (*N* = 9 spp.) or intermediate (*N* = 6 spp.) ability to handle fruits (Dracxler & Kissling, 2021). Around half (44%) of all pulp‐eating records involved animals with low handling ability, predominantly mammals (Appendix S7A). This suggests that pulp‐eating might be a feeding strategy that predominantly occurs among animals that cannot skillfully manipulate palm fruits. Positive outcomes generally dominated, regardless of the level of fruit‐handling ability (Fig. 6; Appendix S7B), suggesting that most pulp‐eaters of palm fruits are mutualists.

#### (a) Pulp removal

The way pulp is removed by animals depends on how animals can handle fruits. Pulp‐eating usually involves pulp extraction in the mouth and subsequent spitting (e.g. ungulates), fruit manipulation with the help of hands or forepaws and the mouth or beak (e.g. primates and macaws), or pulp‐pecking by animals that cannot ingest entire fruits or manipulate them otherwise. The removal of palm mesocarps by pulp‐eaters can negatively affect seed viability when animals (e.g. parrots and peccaries) damage seeds during fruit handling and pulp extraction (Keuroghlian & Eaton, 2009; Villalobos & Bagno, 2012). Our review suggests that, in most cases, pulp‐eaters consume the pulp and discard palm seeds intact, a process that benefits seed survival and germination because the pulp often has chemical inhibitors that prevent seeds from germinating (Robertson *et al*., 2006). Effects of pulp‐eating on seed viability and germination also depend on the amount of pulp that is removed, because remaining pulp can favour attacks by pathogens and invertebrate predators or lead to a faster deterioration of seeds (Herrera, 1982). Among pulp‐eaters of palms, partial pulp removal is mainly observed with animals that have a low handling ability, including birds such as tanagers and blackbirds (e.g. Matos & Watkinson, 1998; Villalobos & Bagno, 2012) or mammals such as peccaries (Fragoso, 1997). Pulp‐eaters with intermediate or high handling ability (e.g. macaws or primates) generally remove most of the pulp and then discard the well‐cleaned palm seeds (e.g. Fragoso, 1997; Villalobos & Bagno, 2012; Franco‐Quimbay & Rojas‐Robles, 2015; Filho, Andrade & Bezerra, 2021). This often leads to high palm seed survival and germination, and thus a positive interaction outcome. Yet, cleaned palm seeds that are discarded by pulp‐eaters can be subsequently eaten by other animals, as is the case for the exotic palm *Elaeis guineensis* in the Atlantic Forest in northeastern Brazil, where the red‐back agouti *Dasyprocta iacki* consumes seeds left over after pulp‐eating by the blonde capuchin *Sapajus flavius* (Filho *et al*., 2021). This highlights the importance of considering secondary fates of seeds discarded by pulp‐eaters.

#### (b) Seed dispersal by ectozoochory

Seeds discarded after pulp consumption are often found below or near parent palms (Kays, 1999; Campos, Steiner & Zillikens, 2012; Franco‐Quimbay & Rojas‐Robles, 2015). However, some mammalian pulp‐feeders with high mobility and a high ability to handle fruits (e.g. primates, rodents, marsupials, cingulates and ungulates) transport palm fruits to other sites or feeding trees, away from parent palms, where they feed on the pulp and then discard the viable and cleaned palm seeds (e.g. Cintra & Horna, 1997; Fragoso *et al*., 2003; Pimentel & Tabarelli, 2004; Beck, 2006; Keuroghlian & Eaton, 2009; Brown, 2011; Silva *et al*., 2011; Acevedo‐Quintero & Zamora‐Abrego, 2016; Canale *et al*., 2016; Junges *et al*., 2018). The dispersal distances are typically rather short (e.g. <50 m), but some large birds such as macaws and parrots (Psittacidae) can transport seeds over long (>200 m) distances (Sazima, 2008; Villalobos & Bagno, 2012; Santos & Ragusa‐Netto, 2014; Baños‐Villalba *et al*., 2017; Blanco *et al*., 2019) (Fig. 7; Appendix S6). These defleshed seeds (i.e. the endocarps) are left on the ground surface (without burial, unlike hoarding seed‐eaters) and do not have protective excreta around the seeds (as provided by fruit‐eaters) (Rios & Pacheco, 2006). Nevertheless, they benefit from pulp removal and dispersal, and seeds can successfully germinate and establish as seedlings (e.g. Campos *et al*., 2012). This might be particularly beneficial if the abiotic conditions at the deposition sites (e.g. humidity or luminosity) match the germination requirements of the seeds, or if desiccation associated with seed exposure facilitates the break of dormancy, germination and establishment (Broschat, 1998). Hence, pulp‐eaters such as macaws can act as legitimate, long‐distance seed dispersers of palms and thus play an important role in connecting palm populations between habitat fragments (Baños‐Villalba *et al*., 2017).

### (3) Seed‐eating

A total of 370 seed‐eating records contained information on interaction outcomes, covering 186 unique interactions involving 70 animal species feeding on the seeds of 62 palm species. More than 60% of these seed‐eating records (*N* = 222) showed dual outcomes and almost 30% resulted in negative outcomes (Fig. 5C; Table 1). Some seed‐eating interactions had positive outcomes (*N* = 41). Interaction outcomes of seed‐eaters were dominated by mammals, mainly involving agoutis and squirrels (Appendix S4).

Most species of seed‐eaters had an intermediate ability to manipulate fruits, including 32 rodent species and five parrots (Dracxler & Kissling, 2021). High handling ability was observed for 27 rodent species and two primate species, while a low handling ability was identified for only four species of rodents and two peccary species. The largest proportion of interaction records involved mammalian seed‐eaters with high handling ability (Appendix S8A), partly due the large number of interaction records involving agoutis (*Dasyprocta* spp.). Seed‐eating interactions mostly resulted in dual outcomes regardless of the handling ability of animals (Appendix S8B).

For 61 out of the 70 seed‐eater species (56 mammals and 5 bird species) we could classify the presence or absence of caching behaviour. Around 79% showed caching behaviour, all of which were rodents (Dracxler & Kissling, 2021). Non‐caching animals included birds (*N* = 5 spp.), rodents (*N* = 4 spp.), primates and ungulates (each represented by two species) (Dracxler & Kissling, 2021). The majority of seed‐eating records (*N* = 316) involved seed‐caching rodents (Appendix S8C), mostly resulting in dual outcomes (*N* = 205 records) (Appendix S8D). By contrast, interaction records involving animals that did not cache seeds predominantly (71%) resulted in negative outcomes (Fig. 6; Appendix S8D).

#### (a) Seed predation and dispersal by non‐caching seed‐eaters

Chances that seeds survive interactions with non‐caching seed‐eaters are small because animals without caching behaviour tend to predate palm seeds (e.g. Silvius, 2002; Beck, 2006; Ragusa‐Netto, 2018). Hence, negative interaction outcomes tend to dominate, independent of their fruit handling ability (Fig. 6; Appendix S8B). Nevertheless, palm seeds handled by non‐caching seed‐eaters with intermediate or high fruit‐handling ability (e.g. psitacids or primates) can escape predation when animals accidentally or actively drop non‐consumed fruits or seeds when moving to other sites before consumption (van der Hoek, Solas & Peñuela, 2019; Tella *et al*., 2020). This can lead to positive effects on seed viability because seeds ultimately escape predation (Appendix S8B).

Seed‐eaters such as parrots and macaws can also promote effective long‐distance dispersal of viable seeds (as far as 1620 m from fruit sources; Tella *et al*., 2020) by flying with fruits or defleshed seeds in their beak to perching trees (Blanco *et al*., 2019; Tella *et al*., 2020). This has been recorded for various palm species (genera *Acrocomia*, *Mauritia*, *Syagrus* and *Attalea*). Although some of the seeds are predated at feeding sites, many are left undamaged below feeding trees and successfully establish as seedlings (Blanco *et al*., 2019; Tella *et al*., 2020). This shows that ectozoochory by non‐caching seed‐eaters can allow the effective dispersal of seeds (Fig. 7; Appendix S6). Nevertheless, it generally remains unknown how common this behaviour is among non‐caching seed‐eaters.

#### (b) Seed caching and dispersal

Interaction outcomes involving seed‐caching animals are dependent on multiple decisions made by the animal (Lichti *et al*., 2017). For example, seeds can be cached and later recovered and eaten either by cache owners or by cache thieves (negative outcomes; Jansen *et al*., 2012). Seeds can also be re‐cached multiple times in different locations (positive or negative outcomes; Jansen *et al*., 2012), or may be forgotten or neglected by cache owners and then establish as seedlings (positive outcomes; Pires & Galetti, 2012). For palms, seed‐caching behaviour has been reported to be positive because seeds are cleaned (which reduces seed perishability) and then dispersed and stored in a way that increases seed viability (e.g. Smythe, 1989; Grenha *et al*., 2010; Pires & Galetti, 2012; Kuprewicz, 2015). Nevertheless, because interaction outcomes are not only positive but also negative (e.g. the proportion of seeds being predated), seed‐eating by scatter‐hoarders is mostly classified as dual outcome (Appendix S8D).

The ratio of surviving or cached seeds to predated seeds ultimately determines the net effects of seed‐caching animals on seed viability (Zwolak & Crone, 2012). This can vary even when the same species (of palm and frugivore) are involved in the interaction (Kuprewicz, 2015). Decision‐making processes and cache management by scatter‐hoarding rodents are highly context dependent (Lichti *et al*., 2017), being influenced by fruit availability, palm abundance, site effects (e.g. forest fragment size) and disturbance (e.g. habitat loss and logging). Scatter‐hoarders tend to cache more seeds than they need when fruit availability is high, leading to mostly positive outcomes (Gálvez *et al*., 2009; Ragusa‐Netto, 2016). However, seed predation rates can increase when food is limited, increasing negative outcomes. This has been documented, for example, for the palm *Astrocaryum aculeatum* and rodents in the Brazilian Amazon (Jorge & Howe, 2009).

Large and long‐lasting seeds are cached more often compared to small seeds with thin endocarps because hoarding animals perceive them as the most valuable food items (Jansen *et al*., 2002; Galetti *et al*., 2010; Mittelman *et al*., 2021). This suggests that interactions involving large, stony palm seeds are more likely to result in positive outcomes because those seeds are preferentially cached instead of immediately consumed. Seed caching can also indirectly favour seed viability by facilitating escape from seed predation by invertebrates such as bruchid beetles (Smythe, 1989; Kuprewicz, 2015). This happens because rodents reduce chances of seed encounter by bruchids, which tend to infest seeds near parent palms. They further exert a top‐down control over bruchid populations by consuming bruchid larvae (i.e. grubivory, *sensu* Silvius, 2002) or removing oviposited larvae during seed cleaning (Peguero *et al*., 2017). This secondary benefit of interactions with seed‐caching animals can be crucial for some palm species that would otherwise experience high invertebrate predation if seeds were left near parent palms (Dracxler, Pires & Fernandez, 2011).

Seed caching usually involves seed dispersal because animals tend to move and cache seeds away from parent palms (Smythe, 1989; Lichti *et al*., 2017). Dispersal distances of palm seeds depend on a combination of factors such as frugivore traits [e.g. body size, movement patterns and home ranges of seed‐caching animals (Carvajal & Adler, 2008; Lichti *et al*., 2017)], fruit availability (Gálvez *et al*., 2009) and palm traits such as seed mass (Galetti *et al*., 2010). The Central‐American agouti *Dasyprocta punctata* can move seeds of *Astrocaryum standleyanum* hundreds of meters away from parent palms because cache owners and thieves re‐cache the seeds multiple times (Jansen *et al*., 2012). Nevertheless, most reports of caching‐associated seed dispersal suggest that scatter‐hoarders tend to disperse seeds to distances <50 m (Forget & Wenny, 2005) (Fig. 7; Appendix S6). For palm species that are exclusively dispersed by scatter‐hoarding rodents, dispersal distances associated with seed caching can strongly limit how far a population can expand (Charles‐Dominique *et al*., 2003). However, commonly used methods to track seeds are limited in how far dispersal distances can be measured (Forget & Wenny, 2005; Mittelman *et al*., 2021).

Seed‐caching rodents may select different microsites to deposit the seeds (Hoch & Adler, 1997). For instance, scatter‐hoarding animals often use spatial cues to recover their caches. They cache seeds near large objects such as fallen trunks (Kiltie, 1981; Smythe, 1989; Hoch & Adler, 1997; Pires & Galetti, 2012), move seeds to forest gaps (Carvajal & Adler, 2008), or deposit them in areas with low density of conspecific adults (Brewer & Webb, 2001; Hirsch *et al*., 2012*b*). Importantly, deposition microsites of seed‐caching animals are not always beneficial for the palms. Squirrels, for example, can place seeds in arboreal caches, a microsite that might not be favourable for the development of palms (Carvajal & Adler, 2008). Nevertheless, seed caching is mostly associated with positive outcomes at the seedling establishment stage because caching behaviour involves palm seed cleaning, storage and burial at depths that favour seed germination and seedling emergence (Vander Wall, 1990).

## VI. MUTUALISM–ANTAGONISM CONTINUUMS: QUANTIFYING INTRA‐ AND INTER‐SPECIFIC VARIATION

**Fig. 8:**
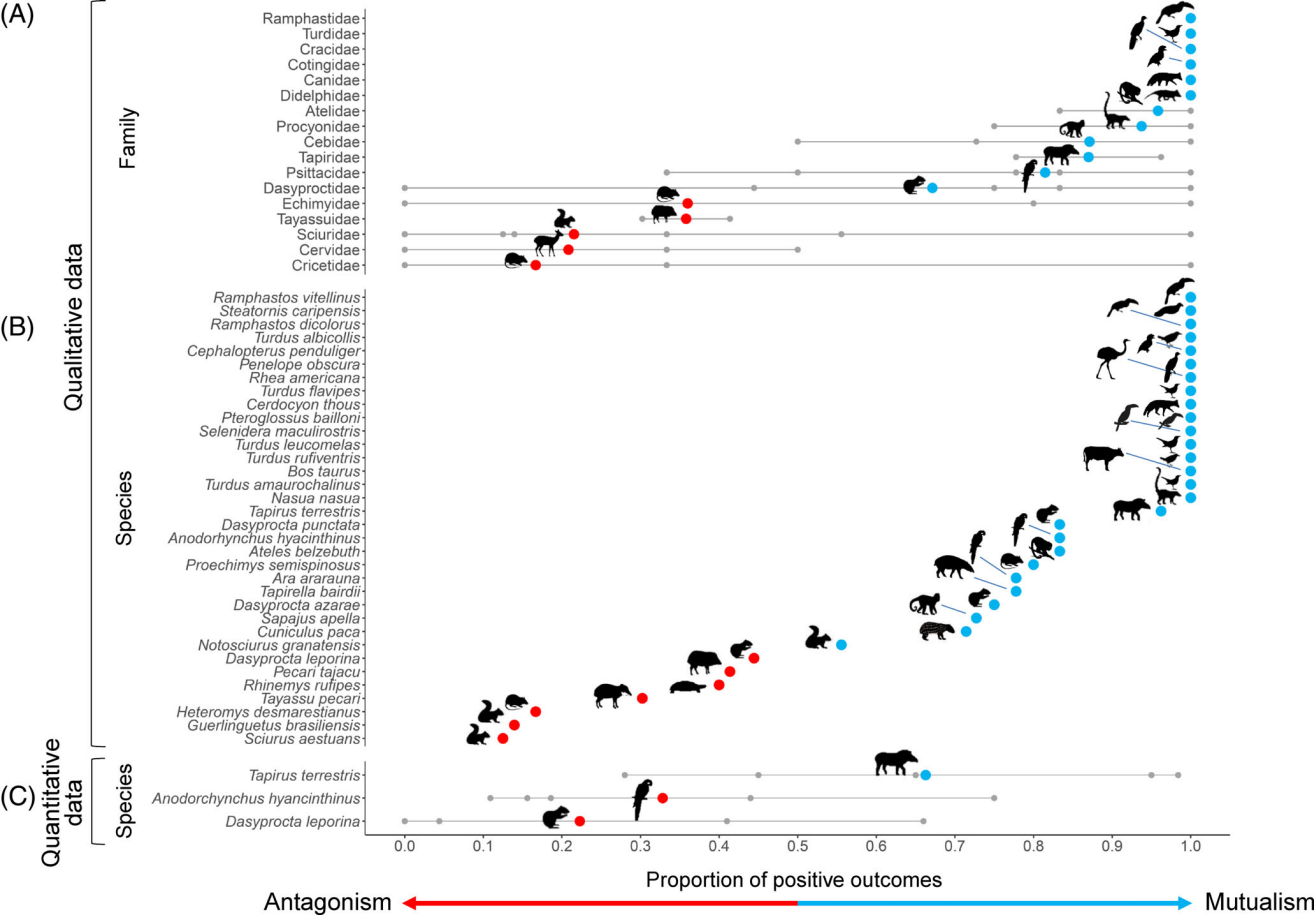
The mutualism–antagonism continuum in Neotropical palm–frugivore interactions. The proportion of positive interaction outcomes from qualitative and quantitative evidence reported in the literature is summarized for frugivore families and species along the mutualism–antagonism continuum (*x*‐axis). The left side of the continuum mostly shows antagonistic families or species (total or mean values in red; proportion of positive outcomes <0.5) and the right side mostly mutualistic species or families (total or mean values in blue; proportion of positive outcomes ≥0.5). Grey dots represent the proportion of positive outcomes for species within families (A) or individual studies within species (C), with grey horizontal lines indicating the range. (A) Qualitative family‐level data summarizing the proportion of positive outcomes across species within 17 frugivore families from 124 papers, with a total of 495 interaction records. The number of species (2–12) and the number of interaction records (mean ± SD: 29.1 ± 23.3, range: 7–83 outcome records) varies per family. (B) Qualitative species‐level data showing the average proportion of positive outcomes for 34 frugivore species with ≥5 outcome observations (mean ± SD: 11.9 ± 11.8, range: 5–53 outcome records) from 111 papers, with a total of 406 interaction records. (C) Quantitative species‐level data for three species of frugivores (the lowland tapir *Tapirus terrestris*, the red‐rumped agouti *Dasyprocta leporina*, and the hyacinth macaw *Anodorhynchus hyacinthinus*) summarizing evidence on the proportion of positive outcomes (five records per species) from six scientific studies. Outcome data are provided in a supplementary file containing interaction records and outcomes extracted from the sources (Continuum data; Dracxler & Kissling, 2021).

Section V documented how outcomes in palm–frugivore interactions can vary among fruit‐eaters, pulp‐eaters, and seed‐eaters and according to their consumption behaviours and traits. In this section we synthesise available evidence on where specific frugivore families and species fall along the mutualism–antagonism continuum and how this position varies along the continuum (Fig. 8). This was done by summarizing variation in interaction outcomes from the best qualitative and quantitative studies available for palms, focusing on the proportion of positive outcomes within individual frugivore species (intra‐specific variability) and across different frugivore species within families (inter‐specific variability).

### (1) Qualitative data

A total of 17 frugivore families had ≥7 studies with qualitative data available at the family level to summarize the proportion of positive outcomes along the mutualism–antagonism continuum (Fig. 8A). These families were represented by a total of 107 animal species recorded in 495 interaction records, and included rodents (Dasyproctidae, Echimyidae, Sciuridae and Cricetidae), ungulates (Tapiridae, Tayassuidae and Cervidae), primates (Atelidae and Cebidae), carnivores (Canidae and Procyonidae), marsupials (Didelphidae) and small (Turdidae) and large birds (Ramphastidae, Cracidae, Cotingidae and Psittacidae). Twelve frugivore families (75%) were classified as mainly mutualistic along the continuum (Fig. 8A), indicating that the majority of species in these families (95%, *N* = 74) provided positive outcomes for palms. Most of the interactions with these animals provided benefits for palms through increased seed survival and seedling establishment after seed treatment. This included even families such as carnivores (Canidae and Procyonidae), parrots (Psittacidae) and rodents (Dasyproctidae) that are rarely perceived as mutualists. In each of two families (Psittacidae, Dasyproctidae) that were classified as mutualistic, two species fall on the antagonistic side of the continuum: two parrots (*Pyrrhura frontalis* and *Triclaria malachitacea*) and two agoutis (*Dasyprocta leporina* and *D. iacki*). This highlights the large inter‐specific variability within parrots and rodents. Other mammalian families with mostly positive outcomes for palms included marsupials (Didelphidae), primates (Atelidae and Cebidae) and ungulates (Tapiridae). Four bird families ranked as highly mutualistic, including toucans (Ramphastidae), thrushes (Turdidae), chachalacas, guans and curassows (Cracidae) and cotingas (Cotingidae). A total of 33 bird species were represented by these four bird families, with a total of 117 interaction outcomes represented in the continuum, all of which involved fruit‐eating and positive outcomes. Canidae and Didelphidae were also classified as fully mutualistic and represented by 11 animal species.

Only five frugivore families (including spiny rats, peccaries, deer and squirrels) fell on the antagonistic side of the continuum, with the average proportion of positive outcomes ranging from 0.17 to 0.36 (Fig. 8A). However, even those families were not exclusively antagonistic because interaction outcomes reported in the literature also indicated positive interaction outcomes for some species. Negative outcomes were mainly explained by the predation of seeds through mastication in fruit‐eating events or by direct consumption (and destruction) of seeds in seed‐eating interactions.

A total of 34 frugivore species had ≥5 studies with qualitative data available at the species level to summarize the proportion of positive interaction outcomes along the mutualism–antagonism continuum (Fig. 8B). Twenty seven species (79%) were predominantly mutualists (with proportion of positive outcomes ≥0.5; Fig. 8B). Sixteen of those were fully mutualistic (proportion of positive outcomes = 1), including two toucans (*Ramphastos vitellinus* and *R. dicolorus*), the oilbird (*Steatornis caripensis*), two toucanets (*Pteroglossus bailloni* and *Selenidera maculirostris*), five thrush species (*Turdus albicollis*, *T. flavipes*, *T. leucomelas*, *T. rufiventris* and *T. amaurochalinus*), the dusky‐legged guan (*Penelope obscura*), the long‐wattled umbrelllabird (*Cephalopterus penduliger*), the greater rhea (*Rhea americana*), the crab‐eating fox (*Cerdocyon thous*), the domestic cow (*Bos taurus*) and the ring‐tailed coati (*Nasua nasua*) (Fig. 8B). The eleven other frugivore species placed on the mutualistic side of the continuum (including two tapirs, two macaws, two monkeys, two agoutis, a paca, a squirrel and a spiny rat) were mutualistic because of their positive role as fruit‐eaters, besides dispersing seeds by ectozoochory, feeding on pulp and discarding intact seeds, and caching seeds. Seven species (21%) were classified as mostly antagonists, including peccaries, squirrels, a spiny rat, an agouti and a turtle species (Fig. 8B). Antagonistic outcomes involving these animals were mainly derived from fruit‐ and seed‐eating interactions, in which seeds are damaged due to fruit mastication or direct seed predation. However, the continuum reveals that none of these species are fully antagonistic because palms can also derive benefits from interactions with these animals. In general, aggregation of outcomes at the species level shows that most animal species fall along the mutualism–antagonism continuum and are neither obligate mutualists nor exclusively antagonists, that is that intra‐specific variation of outcomes is common for most animals interacting with palm fruits.

### (2) Quantitative data

A total of three frugivore species had ≥5 studies available with quantitative information to allow us to summarize trends and intra‐specific variability in the proportion of positive outcomes along the mutualism–antagonism continuum (Fig. 8C). For the lowland tapir (*Tapirus terrestris*), quantitative data were available on seed viability and seedling establishment, representing the proportion of seeds found viable in dung and the germination success of seeds in dung piles, respectively (Rodrigues, Olmos & Galetti, 1993; Fragoso, 1997; Fragoso & Huffman, 2000; Giombini, Bravo & Martinez, 2009). For the hyacinth macaw (*Anodorhynchus hyacinthinus*), quantitative data reflected information on seed damage, either as the proportion of seeds found undamaged after seed dispersal or as the proportion of predated seeds (Tella *et al*., 2020). For the red‐rumped agouti (*Dasyprocta leporina*), quantitative data on seed predation were available as the proportion of seeds predated *versus* cached (Galetti *et al*., 2010). Among these three species, the lowland tapir showed the highest proportion of positive outcomes (mean proportion of positive outcomes = 0.68; Fig. 8C) and was thus clearly a mutualistic frugivore. However, some evidence also indicated a potential antagonistic role in some cases (range of outcomes = 0.28–0.98; Fig. 8C). By contrast, the red‐rumped agouti and the hyacinth macaw predominantly had an antagonistic role for palms (mean proportions of positive outcomes <0.5), although some studies indicated a mutualistic role (Fig. 8C).

The available quantitative evidence of the position and range of species along the mutualism–antagonism continuum shows that outcomes of interactions involving the same frugivore species can vary widely, even for taxa such as the tapir which are generally perceived as mutualistic frugivores. This might explain the lack of consensus in the literature about the ecological roles of some frugivores (Tella *et al*., 2015; Gómez *et al*., 2019; van Leeuwen, Tella & Green, 2020). The red‐rumped agouti provides an interesting example of the variability in interaction outcomes because some studies report the predation of virtually all seeds of some palms (e.g. *Euterpe edulis*, *Syagrus pseudococos* and *S. romanzoffiana*) whereas another study shows a high positive outcome for another palm (66% of *Astrocaryum aculeatissimum* seeds are cached; Fig. 8C) (Galetti *et al*., 2010). Similarly, the hyacinth macaw typically predates the majority of seeds (resulting in a largely antagonistic role) but can also be a mutualist when it discards a large number of undamaged seeds of specific palms (e.g. *Acrocomia totai*, *Attalea barreirensis* and *Attalea eichleri*) below perching trees (Tella *et al*., 2020).

## VII. PROSPECTS

**Fig. 9:**
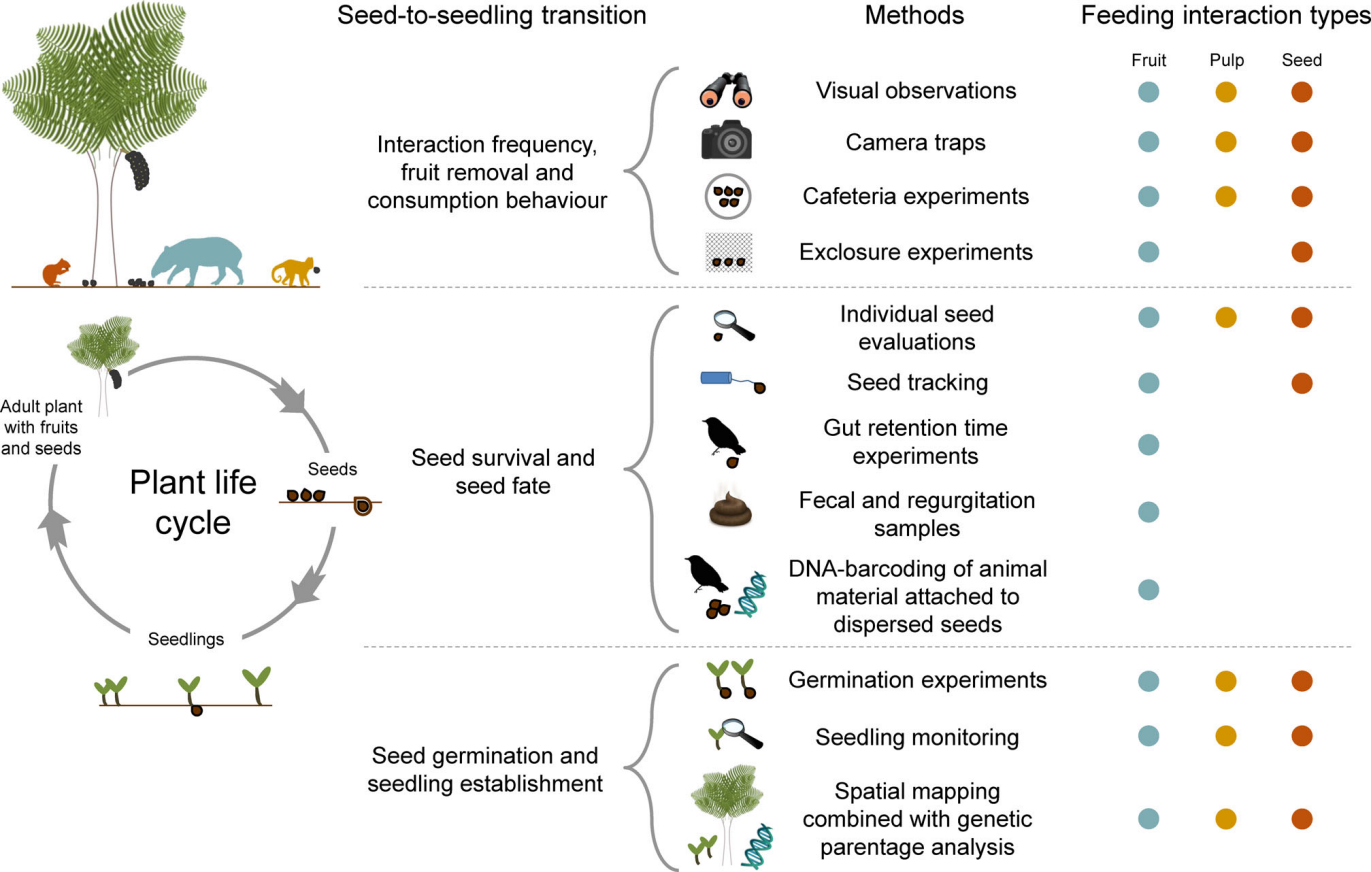
Examples of methods for assessing the effects of frugivores on seed‐to‐seedling transitions, separated by feeding interaction types. The suggested methods allow for assessing interaction aspects such as interaction frequency (e.g. number of visits), fruit removal and consumption behaviour of frugivores, and their effects on plant population dynamics (seed survival and seed fate) and demography (seed germination and seedling establishment). Blue, yellow and orange circles show methods applicable to fruit‐eating (‘fruit’), pulp‐eating (‘pulp’) and seed‐eating (‘seed’), respectively.

To our knowledge, our review provides the most comprehensive evidence to date on the inter‐specific and intra‐specific variability in interaction outcomes of a particular plant–frugivore system. Despite the many studies on palm–frugivore interactions that we have reviewed here, evidence on the positive and negative effects of fruit‐eating, pulp‐eating and seed‐eating on palm demography during the seed‐to‐seedling transition remains scarce. This is due to two main reasons. First, it is often not clear whether animal species recorded in frugivory interactions feed on entire fruits, only on the pulp or only on seeds. This lack of detail on frugivory interactions makes it difficult to gather evidence on the effects of different types of feeding interactions on seed‐to‐seedling transitions. Second, assessing quantitative and qualitative effects of interactions on seed viability, seed deposition, germination and seedling establishment is logistically difficult, time‐consuming and methodologically constrained, and thus rarely conducted (Schupp *et al*., 2010; Beckman *et al*., 2020). Nevertheless, a range of field and laboratory methods are available to study and quantify interaction outcomes (Fig. 9).

### (1) Quantification of interaction outcome variation

Visual observations at focal trees allow identifying which species are involved in interactions and which type of feeding and fruit‐handling ability they have (Fig. 9), for example, whether animals feed on fruits, pulp or seeds and how they handle the fruits and seeds. Repeating this for different types of plant species and different individuals of the same plant species will provide insights into the variability of feeding interaction types within and across frugivorous animal species. Visual observations can further provide quantitative information on fruit removal and interaction frequencies (Schupp *et al*., 2010; Meiga & Christianini, 2020). However, experimental approaches such as cafeteria plots (e.g. Jansen, Bongers & Hemerik, 2004) or exclosure experiments (Hulme, 1994) have an advantage over visual observations because they provide a semi‐controlled setting to evaluate the number of fruits or seeds taken by animals. When used in combination with camera traps, such experiments are effective ways for evaluating species‐specific visitation and fruit or seed removal rates, as well as *in situ* pulp consumption (Jansen *et al*., 2012; Acevedo‐Quintero & Zamora‐Abrego, 2016; Campos *et al*., 2018; Meiga & Christianini, 2020; Selwyn *et al*., 2020).

Following seed survival and seed fates after fruits are taken by frugivores (Fig. 9) is challenging because visual observations are limited in measuring seed viability and dispersal distances when seeds are deposited away from parent plants. Large‐bodied fruit‐eaters can move long distances before they deposit seeds (Nathan, 2006) and scatter‐hoarding rodents may move seeds in multiple steps (Jansen *et al*., 2012), making it difficult to assess outcomes after fruit removal. Misrepresentations of interaction outcomes can emerge if fruit or seed removal is assumed (rather than measured) to result either in mutualisms or antagonisms. For instance, fruit‐eating is often assumed to be equivalent to effective dispersal (mutualism) and seed or fruit removal by seed‐eaters is assumed to be equivalent to seed predation (antagonism) (Gómez *et al*., 2019). However, such conclusions can only be drawn accurately through careful evaluation of individual seeds sampled after interactions have taken place. Measuring seed fate can be done by tracking individual seeds (Fig. 9), e.g. *via* marking seeds through stable isotope enrichment of fruiting trees (Carlo, Tewksbury & del Río, 2009) or by tagging seeds, e.g. with spool‐and‐line or telemetric thread tags (Forget & Wenny, 2005; Hirsch, Kays & Jansen, 2012*a*). These methods also provide information on dispersal distances (Forget & Wenny, 2005; Jansen *et al*., 2012), although fixed‐tagging methods may reduce seed movement and constrain the evaluation of dispersal distances. Applying DNA‐barcoding protocols to identify disperser species from samples of regurgitated or defecated seeds is also a powerful method which allows linking disperser identity to fruit trait choices and seed deposition patterns (González‐Varo, Arroyo & Jordano, 2014). Assessing whether individual retrieved seeds (e.g. in caches, on the forest floor, or in animal dung) are intact after gut treatment by fruit‐eaters, after pulp removal by pulp‐eaters or if seed‐eaters ultimately leave seeds undamaged is necessary to inform the effects of frugivores on seed survival. This might also require manually opening endocarps or using X‐rays to assess endosperm integrity because apparently intact endocarps can actually be infested by bruchid larvae (Brancalion *et al*., 2011; Dracxler *et al*., 2011). Controlled assessments of the effects of mouth or beak and gut treatment on seeds and gut retention times (Fig. 9) – including captive frugivore feeding trials combined with germination experiments – are also useful for estimating the effects of fruit‐eaters on seed viability (Samuels & Levey, 2005; Fricke *et al*., 2019), and for quantifying seed dispersal kernels (Schurr *et al*., 2009; Pires *et al*., 2018; Sorensen *et al*., 2020).

A number of methods exist to assess how germination and seedling establishment is affected by the seed treatment of frugivores (Fig. 9) (Snell *et al*., 2019). Experimental approaches in the laboratory or in the field can evaluate germination success of gut‐passed, handled (e.g. defleshed) or cached seeds (Samuels & Levey, 2005; Kuprewicz, 2015), and when conducted at mid‐ or long‐term, can also show effects of frugivores on seedling survival and transitions into later ontogenetic stages (e.g. the sapling stage). Field observations (e.g. mapping or monitoring of seedlings) can further inform about the net effect of frugivores on seed‐to‐seedling transitions (e.g. seedlings established from seeds found in dung or buried in caches) (Fragoso, 1997; Beckman & Rogers, 2013; Sica *et al*., 2014; Dracxler & Forget, 2017). Finally, an approach that combines a spatial assessment of the distribution of seedlings and adults (e.g. source trees) with molecular techniques such as genetic parentage analysis (Sezen *et al*., 2009; Choo, Juenger & Simpson, 2012; Giombini, Bravo & Tosto, 2016; Giombini *et al*., 2017; Browne & Karubian, 2018*b*; Diaz‐Martin & Karubian, 2021) is a way to shed light on how frugivory influences the spatial and genetic structure of palm populations. The non‐exhaustive list of methods provided here (Fig. 9) illustrates exciting opportunities for deepening our knowledge about the effects of frugivores on interaction outcomes and plant demography.

### (2) Influence of fruit traits on feeding types and interaction outcomes

**Fig. 10:**
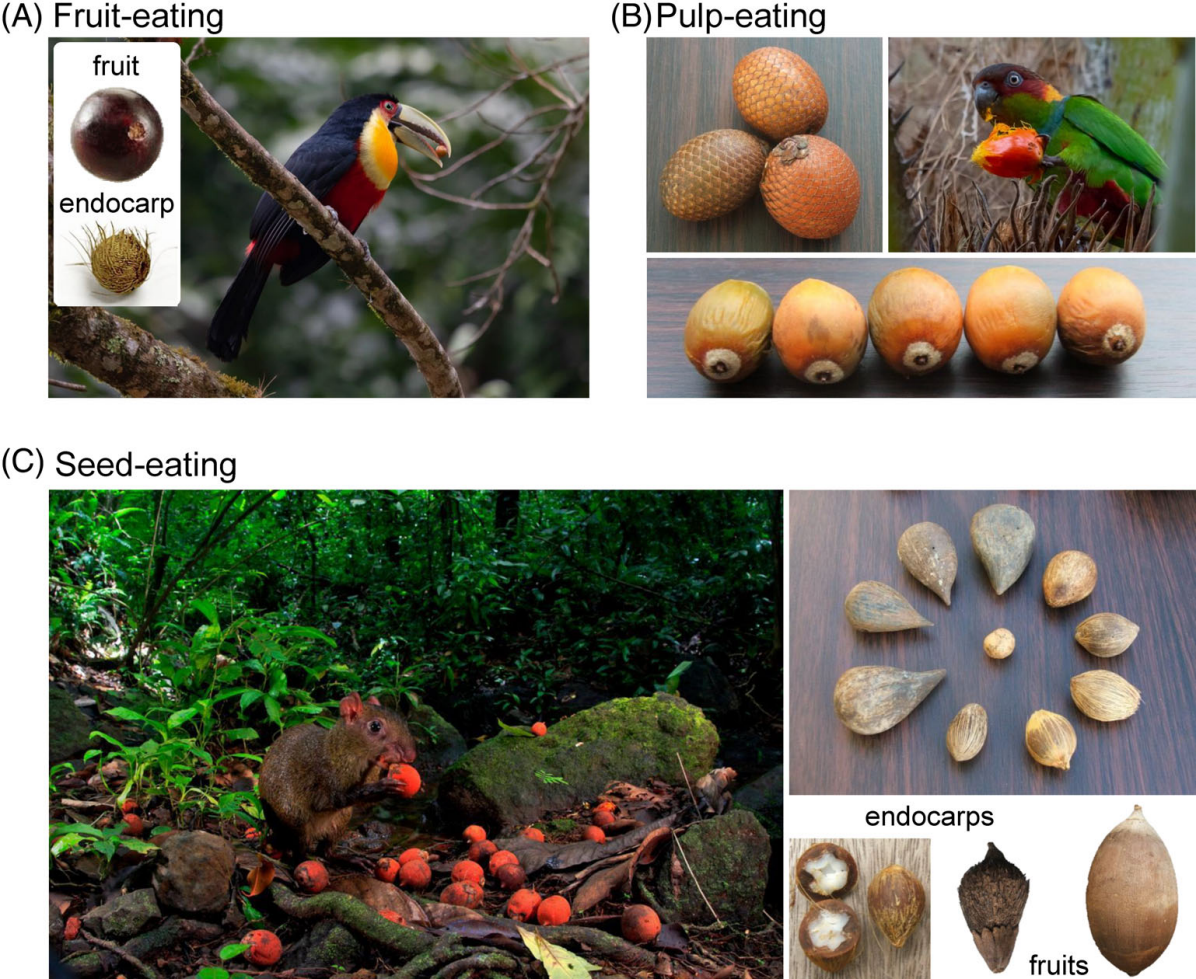
Variation of palm fruit traits and types of feeding interactions, and their influence on interaction outcomes. (A) Fruit‐eating: the fleshy‐fibrous fruits of the palm *Euterpe edulis* (show in the inset) are eaten whole mainly by birds, such as the red‐breasted toucan *Ramphastos dicolorus*, involving interactions that tend to result in positive outcomes (mainly *via* regurgitation of viable endocarps). Their thin endocarps, however, are destroyed when consumed by rodents or ungulates, resulting in negative outcomes. (B) Pulp‐eating interactions usually involve consumption of fleshy or fleshy‐fibrous fruits such as those of the palm genus *Mauritiella* (top left) and *Syagrus* (bottom), by animals such as the ochre‐marked parakeet *Pyrrhura cruentata* (shown feeding on the pulp of the African oil palm *Elaeis guineensis*, an exotic palm species in the Americas). (C) Seed‐eating is very frequent among scatter‐hoarding rodents such as the Central American agouti *Dasyprocta punctata*, which consumes the endosperm (white) enclosed in stony endocarps such as those found in *Astrocaryum* and *Attalea* palms (bottom right). These interactions often result in positive outcomes due to caching behaviour, but variation of endocarp traits (top right and bottom middle; endocarps of different palm genera) influences the decision‐making process by animals and thus interaction outcomes. Photograph credits: A, Toucan – Mathias Pires, fruit and endocarp – Caroline Dracxler; B, Parakeet – Mathias Pires, fruits – Caroline Dracxler; C, Agouti – Christian Ziegler, fruits and endocarps – Caroline Dracxler.

Variation of interaction outcomes within types of feeding interactions might partly be driven by variation in palm fruit and seed traits (Fig. 10). For example, fruits with stony endocarps are found in palm genera such as *Astrocaryum*, *Attalea* and *Syagrus*, sometimes with needle‐like spines on the exocarp, a fibrous mesocarp, and a lipid‐rich endosperm (Fig. 10C). These are mainly consumed by scatter‐hoarding rodents (i.e. seed‐eaters), but because these palm seeds are ideal for storage (i.e. long‐lasting, large nutrient‐rich endocarps), the interactions often result in mutualisms rather than antagonisms due to high caching rates (Jorge & Howe, 2009; Galetti *et al*., 2010; Klinger & Rejmánek, 2010; Jansen *et al*., 2012; Dracxler & Forget, 2017). On the other hand, when rodents feed on small fruits that have rather thin endocarps – such as the fruits of *Euterpe edulis* which have a fleshy‐fibrous pulp and are mainly consumed by birds (Fig. 10A) – interactions with rodents may predominantly result in seed predation rather than seed caching (Pizo, Von Allmen & Morellato, 2006; Galetti *et al*., 2013). Similarly, seeds enclosed in thick endocarps are more likely to survive mouth, beak and gut treatment of fruit‐eaters compared to fruits with thin seed coats (Bodmer & Ward, 2006). The type of pulp in palm fruits (e.g. farinaceous, fibrous or fleshy) might also interfere with how easily seeds are defleshed by pulp‐eaters (Fig. 10B), in turn influencing whether seeds are left undamaged after pulp removal. Future studies should also focus on understanding how palm traits might influence associations with different groups of animals and/or feeding guilds and whether the emergence of positive and negative outcomes can be predicted by palm traits. A workable hypothesis is that fruits sharing specific combinations of traits (e.g. red‐ and purple‐coloured fruits, <2 cm in diameter, and with fleshy pulp such as in *Euterpe*, *Geonoma* and *Oenocarpus*; Kissling *et al*., 2019) that have evolved in response to selection by certain groups of frugivores (e.g. large‐bodied birds) are more likely to benefit from interactions with such groups of frugivores than with other types of frugivores (e.g. rodents). Combining different plant traits (Gautier‐Hion *et al*., 1985; Nascimento *et al*., 2020) with data on trait‐matching relationships (Bender *et al*., 2018) and interaction outcomes (as reviewed here) may provide novel insights into how mutualisms and antagonisms in plant–frugivore interactions have evolved (Guimarães Jr, 2020) and how they could be used to project future changes in communities of interacting species (Schleuning *et al*., 2020).

### (3) Influence of frugivory on plant and ecosystem dynamics

**Fig. 11:**
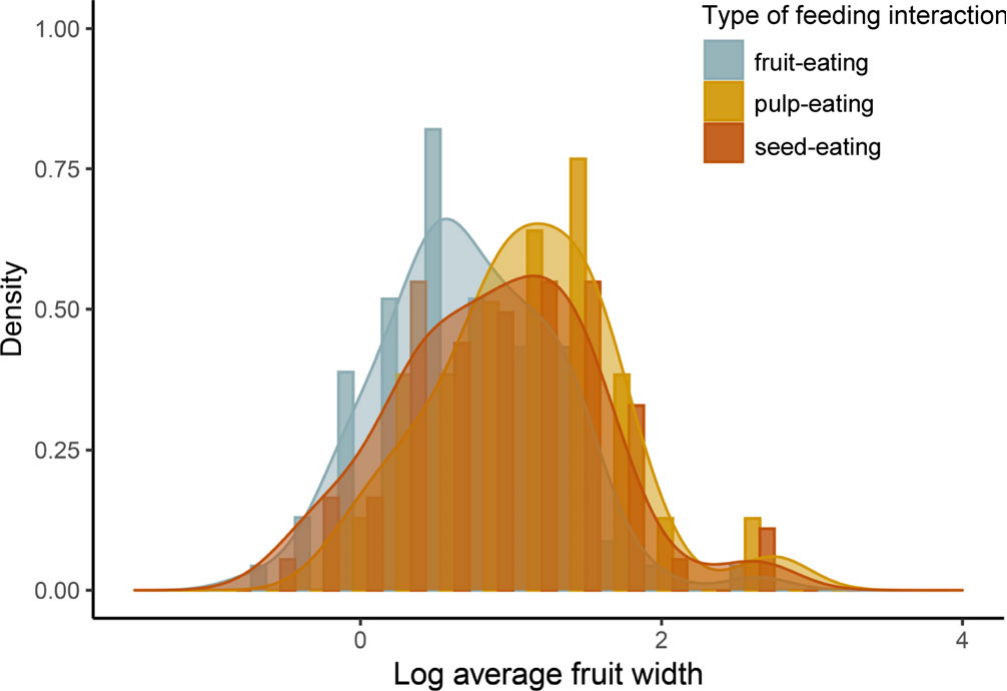
Frequency distribution of palm fruit sizes recorded in different types of feeding interactions with frugivores (fruit‐, pulp‐ and seed‐eating). Fruit size measurements (average fruit width in cm; log‐transformed data) were extracted from PalmTraits 1.0 (Kissling *et al*., 2019) for all palm species (*N* = 106 species) that are captured in the data set of palm–frugivore interaction records (Dracxler & Kissling, 2021). Fruit sizes of 80, 27 and 63 palm species are included for fruit‐, pulp‐ and seed‐eating, respectively (Appendix S9).

In recent years, studies have helped to consolidate functional and trait‐based approaches that link frugivore species to ecosystem dynamics and ecosystem functioning (Schleuning, Fründ & García, 2015). Emerging evidence supports a positive effect of large‐bodied frugivores on carbon storage in tropical forests and thus emphasizes an important role of fruit‐eaters in biogeochemical cycles (Bello *et al*., 2015). Animals such as ungulates and rodents further may affect other biogeochemical cycles such as the nitrogen cycle (Villar *et al*., 2020). To date, the importance of seed‐ and pulp‐eaters for biogeochemical cycles in tropical ecosystems remains little studied, although it is known that seed‐caching animals tend to cache large seeds and predate small ones, thus selecting for palm species that are likely to store more carbon (Mittelman *et al*., 2021). Scatter‐hoarding rodents such as agoutis (*Dasyprocta* spp.) can disperse seeds larger than those dispersed by megaherbivores such as tapirs and large‐bodied monkeys (*Tapirus* spp., *Brachyteles* spp. and *Ateles* spp.) (Mittelman *et al*., 2021). Particular seed‐eaters might thus contribute more to carbon storage than the remaining extant Neotropical megaherbivores. The type of feeding interaction can influence the animal's contribution to carbon storage if fruit and seed size are positively correlated with carbon storage capacity of trees (Fig. 11). Fruit‐eating is often characterized by size matching, that is large‐bodied fruit‐eaters disperse both large and small palm seeds whereas small‐bodied fruit‐eaters can only disperse small palm seeds. However, pulp‐ and seed‐eaters are able to disperse seeds that are larger than their mouth or beak because they can transport seeds externally (either by ectozoochory or synzoochory) (Fig. 11). This suggests that animal species which are typically considered as non‐mutualistic can actually contribute to palm population dynamics and ecosystem functions such as carbon storage. This contribution, however, depends on the interaction outcomes with frugivores, emphasizing the need to assess intra‐specific variation of the effects of frugivores on the several stages of the plant life cycle (Fig. 9).

Assessing how disturbances and novel communities can influence interaction outcomes of palm–frugivore interactions is of fundamental importance for predicting the consequences of habitat loss and modification for palm population dynamics (Jorge & Howe, 2009; Klinger & Rejmánek, 2010; Schupp *et al*., 2019; Escobar *et al*., 2020; Lamperty, Karubian & Dunham, 2021). Habitat and climate change can also alter palm phenology (e.g. Andreazzi *et al*., 2012; Tucker Lima *et al*., 2018; Pedroso *et al*., 2021), and the physiological and ecological responses of both palms and frugivores to habitat and climate change can have cascading effects on palm regeneration. Quantifying how seed dispersal distances vary according to palm fruit traits and feeding guilds of frugivores is also crucial for forecasting palm species distributions under climate change (Butler & Larson, 2020; Sales *et al*., 2021). Pulp‐eaters and seed‐eaters may not be able to disperse palm seeds as far as fruit‐eaters can, but certain frugivores in these feeding guilds provide highly relevant long‐distance dispersal services for several palms (Fig. 7). Ongoing defaunation might mean that seed‐ and pulp‐eaters can take on the role as main dispersers of some large‐fruited palm species (e.g. Meiga & Christianini, 2020). Since palms are particularly abundant in the Neotropics (ter Steege *et al*., 2013; Muscarella *et al*., 2020), understanding the interactions with different feeding guilds and projecting the consequences for palm demography and ecosystem dynamics and functioning should be of high priority.

## VIII. CONCLUSIONS

1. Frugivory involves both advantages and disadvantages for animal‐dispersed plants. Because the effects of frugivory on the seed‐to‐seedling transition in plants vary both within and among animal species, assessing intra‐ and inter‐specific variation of interaction outcomes along a mutualism–antagonism continuum is essential for understanding the consequences of frugivory for plant demography and population and ecosystem dynamics.
2. Frugivory can involve fruit‐eating, pulp‐eating and seed‐eating. Each of these types of feeding interactions can have different influences on seed viability, seed deposition and seedling establishment of plants. Based on a comprehensive compilation of 1043 Neotropical palm–frugivore interaction records that capture variation of feeding interaction types – covering 273 species of birds, mammals, fish and reptiles feeding on the fruits, pulp and seeds of 106 palm species (40 palm genera) – we show that the majority of interactions involve fruit‐eating (545 records; birds, mammals, fish and reptiles), followed by seed‐eating (383 records; birds and mammals) and pulp‐eating (115 records; birds and mammals).
3. All types of feeding interactions can result in positive, negative and dual outcomes for palms. However, the majority of fruit‐eating (77%) and pulp‐eating (91%) results in mutualistic outcomes whereas seed‐eating mostly results in dual outcomes (60%). Variation in interaction outcomes can partly be explained by the consumption behaviour of animals, e.g. in relation to digestive processing (seed defecation *versus* regurgitation), caching behaviour (caching *versus* non‐caching) and fruit‐handling ability (high, intermediate, low). Positive interaction outcomes can be derived from mechanisms such as gut treatment (e.g. leading to increased seed viability and better seed germination after gut passage), endozoochory (e.g. long‐distance seed dispersal by large‐bodied fruit‐eaters), ectozoochory and pulp removal (e.g. discarding of viable and defleshed seeds after dispersal by pulp‐eaters), or seed caching (e.g. facilitation of seedling establishment after seeds are forgotten or neglected by cache owners). Negative outcomes emerge when seeds are damaged during fruit, pulp or seed consumption, or when seeds are deposited in sites that are non‐suitable for germination and establishment.
4. Mutualism–antagonism continuums illustrate how outcomes of plant–frugivore interactions vary intra‐ and inter‐specifically. The mutualism–antagonism continuums for palm–frugivore interactions reveal that most animal species can act as both mutualists and antagonists. The frequency of such outcomes ultimately determines the predominant contribution of frugivores to palm population dynamics and their position along the continuum. These species‐level continuums show that ungulates can be highly mutualistic (e.g. the lowland tapir *Tapirus terrestris*), but also predominantly antagonistic (e.g. the white‐lipped peccary *Tayassu pecari* and the collared peccary *Pecari tajacu*). Similarly, rodents fall both on the mutualistic side (e.g. the paca *Cuniculus paca*, the Central‐American agouti *Dasyprocta punctata* and the red‐tailed squirrel *Notosciurus granatensis*) and on the antagonistic side (e.g. the red‐rumped agouti *D. leporina* and the Brazilian squirrel *Guerlinguetus brasiliensis*). Most bird species, on the other hand, fall on the mutualistic side of the continuum. Family‐level continuums further highlight the contribution of overlooked mutualists such as carnivores (Canidae and Procyonidae) and marsupials (Didelphidae) and reinforce the mostly antagonistic nature of animal groups such as certain rodents or ungulate families.
5. Evaluating the full range of positive and negative effects of fruit‐eating, pulp‐eating and seed‐eating on plant demography during the seed‐to‐seedling transition is challenging. Nevertheless, a range of methods exists to quantify how animals affect seed viability, seed deposition and seedling establishment of plants. Besides visual observations at focal trees, experimental approaches in the laboratory and field can quantify fruit removal rates (e.g. with cafeteria plots or exclosure experiments) and the effects of frugivores on seed survival and seedling establishment (e.g. with germination experiments of gut‐passed, handled or cached seeds). The application of relatively recent and novel methods such as camera traps, individual seed tracking with telemetric thread tags, or molecular techniques such as genetic parentage analysis considerably expand the toolbox for assessing the effects of frugivores on plant population dynamics and demography. Trait‐based approaches will further help to elucidate the link between fruit‐, pulp‐ and seed‐eaters and ecosystem functions such as carbon storage.
6. Our review is a first step towards synthesizing the effect of different consumption‐related behavioural traits and feeding guilds of vertebrate fruit consumers on plant regeneration and ecosystem functioning. While a large amount of evidence is available for fruit‐ and seed‐eaters, little is still known about how pulp‐eaters or certain groups of fruit‐eaters (e.g. frugivorous fish and reptiles) influence seed‐to‐seedling transitions in animal‐dispersed plants. Moreover, quantitative evidence of intra‐ and inter‐specific variability in outcomes of plant–frugivore interactions along the mutualism–antagonism continuum remains limited, as does information on seed dispersal distances. Filling this gap will require more studies that link feeding behaviour and animal traits to the effects of frugivores on seed viability, seed deposition and seedling establishment, and the consideration of how different feeding types and their interaction outcomes affect ecosystem functions and dynamics.

## Supporting information


**Appendix S1.** Combination of search terms for literature compilation in the *Web of Science*.
**Appendix S2.** Interaction types and criteria to classify interactions into positive, negative or dual outcomes based on quantitative and qualitative evidence from published articles on palm–frugivore interactions.
**Appendix S3.** List of palm species by tribe and genera recorded in interactions with frugivores in the data set with types of feeding interactions.
**Appendix S4.** Number of animal species and interaction records in different types of feeding interactions (fruit‐, pulp‐, and seed‐eating), summarized for each family, order and class.
**Appendix S5.** The role of digestive‐processing types for fruits in interactions between fruit‐eaters and palms.
**Appendix S6.** Dispersal distances recorded for interactions between vertebrate frugivores and Neotropical palm species.
**Appendix S7.** The role of fruit‐handling ability in interactions between pulp‐eaters and palms.
**Appendix S8.** The role of handling ability and seed‐caching behaviour in interactions between seed‐eaters and palms.
**Appendix S9.** Fruit size of palm species recorded in interactions with frugivores, according to parts of fruits consumed by animals.

## References

[brv12809-bib-0001] Acevedo‐Quintero, J. F. & Zamora‐Abrego, J. G. (2016). Role of mammals on seed dispersal and predation processes of *Mauritia flexuosa* (Arecaceae) in the Colombian Amazon. Revista de Biologia Tropical 64, 5–15. 28861988 10.15517/rbt.v64i1.18157

[brv12809-bib-0002] Acevedo‐Quintero, J. F. , Zamora‐Abrego, J. G. & Ortega‐León, Á. M. (2018). The prickles of *Astrocaryum malybo* as a structural defense to avoid seed predation. Food Webs 16, e00088.

[brv12809-bib-0003] * Adler, G. H. & Kestell, D. W. (1998). Fates of Neotropical tree seeds influenced by spiny rats (*Proechimys semispinosus*). Biotropica 30, 677–681.

[brv12809-bib-0004] * Aliaga‐Rossel, E. & Fragoso, J. M. (2015). Defaunation affects *Astrocaryum gratum* (Arecales: Arecaceae) seed survivorship in a sub‐montane tropical forest. Revista de Biología Tropical 63, 57–67. 26299115 10.15517/rbt.v63i1.13042

[brv12809-bib-0005] * Alves, B. C. , Mendes, C. P. & Ribeiro, M. C. (2018). Queen palm fruit selection and foraging techniques of squirrels in the Atlantic Forest. Biotropica 50, 274–281.

[brv12809-bib-0006] Anderson, J. T. , Nuttle, T. , Saldaña Rojas, J. S. , Pendergast, T. H. & Flecker, A. S. (2011). Extremely long‐distance seed dispersal by an overfished Amazonian frugivore. Proceedings of the Royal Society B: Biological Sciences 278, 3329–3335. 10.1098/rspb.2011.0155 PMC3177626 21429923

[brv12809-bib-0007] * Andrade, P. C. , Mota, J. V. L. & Carvalho, A. A. F. (2013). Interações mutualísticas entre aves frugívoras e plantas em um fragmento urbano de Mata Atlântica, Salvador, BA. Revista Brasileira de Ornitologia 19, 63–73.

[brv12809-bib-0008] Andreazzi, C. S. , Pires, A. S. & Fernandez, F. A. (2009). Mamíferos e palmeiras neotropicais: interações em paisagens fragmentadas. Oecologia Brasiliensis 13, 554–574.

[brv12809-bib-0009] Andreazzi, C. S. , Pimenta, C. S. , Pires, A. S. , Fernandez, F. A. S. , Oliveira‐Santos, L. G. & Menezes, J. F. S. (2012). Increased productivity and reduced seed predation favor a large‐seeded palm in small Atlantic Forest fragments. Biotropica 44, 237–245.

[brv12809-bib-0010] * Ballari, S. A. , Cuevas, M. F. , Ojeda, R. A. & Navarro, J. L. (2015). Diet of wild boar (*Sus scrofa*) in a protected area of Argentina: the importance of baiting. Mammal Research 60, 81–87.

[brv12809-bib-0011] Baños‐Villalba, A. , Blanco, G. , Diaz‐Luque, J. A. , Denes, F. V. , Hiraldo, F. & Tella, J. L. (2017). Seed dispersal by macaws shapes the landscape of an Amazonian ecosystem. Scientific Reports 7, 7373. 28785083 10.1038/s41598-017-07697-5 PMC5547140

[brv12809-bib-0012] Barbosa, T. A. P. & Montag, L. F. A. (2017). The role of *Lithodoras dorsalis* (Siluriformes: Doradidae) as seed disperser in Eastern Amazon. Neotropical Ichthyology 15, e160061.

[brv12809-bib-0013] Barcelos, A. R. , Bobrowiec, P. E. D. , Sanaiotti, T. M. & Gribel, R. (2013). Seed germination from lowland tapir (*Tapirus terrestris*) fecal samples collected during the dry season in the northern Brazilian Amazon. Integrative Zoology 8, 63–73. 23586561 10.1111/1749-4877.12003

[brv12809-bib-0014] Beck, H. (2006). A review of peccary‐palm interactions and their ecological ramifications across the Neotropics. Journal of Mammalogy 87, 519–530.

[brv12809-bib-0015] Beckman, N. G. , Aslan, C. E. , Rogers, H. S. , Kogan, O. , Bronstein, J. L. , Bullock, J. M. , Hartig, F. , HilleRisLambers, J. , Zhou, Y. , Zurell, D. , Brodie, J. F. , Bruna, E. M. , Cantrell, R. S. , Decker, R. R. , Efiom, E. , et al. (2020). Advancing an interdisciplinary framework to study seed dispersal ecology. AoB Plants 12, plz048. 32346468 10.1093/aobpla/plz048 PMC7179845

[brv12809-bib-0016] * Beckman, N. G. & Muller‐Landau, H. C. (2007). Differential effects of hunting on pre‐dispersal seed predation and primary and secondary seed removal of two Neotropical tree species. Biotropica 39, 328–339.

[brv12809-bib-0017] Beckman, N. G. & Rogers, H. S. (2013). Consequences of seed dispersal for plant recruitment in tropical forests: interactions within the seedscape. Biotropica 45, 666–681.

[brv12809-bib-0018] Bello, C. , Galetti, M. , Pizo, M. A. , Magnago, L. F. S. , Rocha, M. F. , Lima, R. A. , Peres, C. A. , Ovaskainen, O. & Jordano, P. (2015). Defaunation affects carbon storage in tropical forests. Science Advances 1, e1501105. 26824067 10.1126/sciadv.1501105 PMC4730851

[brv12809-bib-0019] Bender, I. M. A. , Kissling, W. D. , Blendinger, P. G. , Böhning‐Gaese, K. , Hensen, I. , Kühn, I. , Muñoz, M. C. , Neuschulz, E. L. , Nowak, L. , Quitián, M. , Saavedra, F. , Santillán, V. , Töpfer, T. , Wiegand, T. , Dehling, D. M. , et al. (2018). Morphological trait matching shapes plant–frugivore networks across the Andes. Ecography 41, 1910–1919.

[brv12809-bib-0020] Blanco, G. , Tella, J. L. , Diaz‐Luque, J. A. & Hiraldo, F. (2019). Multiple external seed dispersers challenge the megafaunal syndrome anachronism and the surrogate ecological function of livestock. Frontiers in Ecology and Evolution 7, 328.

[brv12809-bib-0021] Bodmer, R. & Ward, D. (2006). Frugivory in large mammalian herbivores. In Large Herbivore Ecology, Ecosystem Dynamics and Conservation (eds K. Danell , P. Duncan , R. Bergström and J. Pastor ), pp. 232–260. Cambridge University Press, Cambridge.

[brv12809-bib-0022] * Bordignon, M. , Margarido, T. C. C. & Lange, R. R. (1996). Formas de abertura dos frutos de *Syagrus romanzoffiana* (Chamisso) Glassman efetuadas por *Sciurus ingrami* Thomas (Rodentia, Sciuridae). Revista Brasileira de Zoologia 13, 821–828.

[brv12809-bib-0023] * Bossi, M. A. S. , Migliorini, R. P. , Santos, T. G. & Kasper, C. B. (2019). Comparative trophic ecology of two sympatric canids in the Brazilian Pampa. Journal of Zoology 307, 215–222.

[brv12809-bib-0024] Bowler, M. & Bodmer, R. E. (2011). Diet and food choice in Peruvian red Uakaris (*Cacajao calvus ucayalii*): selective or opportunistic seed predation? International Journal of Primatology 32, 1109–1122.

[brv12809-bib-0025] Brancalion, P. H. , Rodrigues, R. R. , Novembre, A. D. & Gómez, J. M. (2011). Are we misinterpreting seed predation in palms? Biotropica 43, 12–14.

[brv12809-bib-0026] * Brewer, S. W. (2001). Predation and dispersal of large and small seeds of a tropical palm. Oikos 92, 245–255.

[brv12809-bib-0027] * Brewer, S. W. & Rejmánek, M. (1999). Small rodents as significant dispersers of tree seeds in a Neotropical forest. Journal of Vegetation Science 10, 165–174.

[brv12809-bib-0028] Brewer, S. W. & Webb, M. A. H. (2001). Ignorant seed predators and factors affecting the seed survival of a tropical palm. Oikos 93, 32–41.

[brv12809-bib-0029] Bronstein, J. L. (1994). Conditional outcomes in mutualistic interactions. Trends in Ecology & Evolution 9, 214–217. 21236825 10.1016/0169-5347(94)90246-1

[brv12809-bib-0030] Bronstein, J. L. (2001). The exploitation of mutualisms. Ecology Letters 4, 277–287.

[brv12809-bib-0031] * Brooks, D. M. , Bodmer, D. M. & Matola, S. (1997). Status Survey and Conservation Action Plan‐Tapirs. IUCN/SSC Tapir Specialist Group‐Action Plan, Gland.

[brv12809-bib-0032] Broschat, T. K. (1998). Endocarp removal enhances *Butia capitata* (Mart.) Becc. (pindo palm) seed germination. HortTechnology 8, 586–587.

[brv12809-bib-0033] Brown, D. D. (2011). Fruit‐eating by an obligate insectivore: palm fruit consumption in wild northern tamanduas (*Tamandua mexicana*) in Panamá. Edentata 12, 63–65.

[brv12809-bib-0034] Browne, L. & Karubian, J. (2018a). Habitat loss and fragmentation reduce effective gene flow by disrupting seed dispersal in a neotropical palm. Molecular Ecology 27, 3055–3069. 29900620 10.1111/mec.14765

[brv12809-bib-0035] Browne, L. & Karubian, J. (2018b). Rare genotype advantage promotes survival and genetic diversity of a tropical palm. New Phytologist 218, 1658–1667. 29603256 10.1111/nph.15107

[brv12809-bib-0036] Browne, L. , Ottewell, K. & Karubian, J. (2015). Short‐term genetic consequences of habitat loss and fragmentation for the neotropical palm *Oenocarpus bataua* . Heredity 115, 389–395. 25920669 10.1038/hdy.2015.35 PMC4611232

[brv12809-bib-0037] Butler, C. J. & Larson, M. (2020). Climate change winners and losers: the effects of climate change on five palm species in the southeastern United States. Ecology & Evolution 10, 10408–10425. 33072269 10.1002/ece3.6697 PMC7548205

[brv12809-bib-0038] * Cabral, S. D. O. , Freitas, I. D. S. , Morlanes, V. , Katzenberger, M. & Calabuig, C. (2019). Potential seed dispersers: a new facet of the ecological role of *boa constrictor constrictor* Linnaeus 1758. Biota Neotropica 19, e20180626.

[brv12809-bib-0039] Cámara‐Leret, R. , Faurby, S. , Macía, M. J. , Balslev, H. , Göldel, B. , Svenning, J.‐C. , Kissling, W. D. , Rønsted, N. & Saslis‐Lagoudakis, C. H. (2017). Fundamental species traits explain provisioning services of tropical American palms. Nature Plants 3, 16220. 28112717 10.1038/nplants.2016.220

[brv12809-bib-0040] * Camilo‐Alves, C. S. P. & Mourão, G. M. (2010). Palms use a bluffing strategy to avoid seed predation by rats in Brazil. Biotropica 42, 167–173.

[brv12809-bib-0041] Campos, R. C. , Steiner, J. & Zillikens, A. (2012). Bird and mammal frugivores of *Euterpe edulis* at Santa Catarina Island monitored by camera traps. Studies on Neotropical Fauna and Environment 47, 105–110.

[brv12809-bib-0042] Campos, C. M. , Velez, S. , Miguel, M. F. , Papú, S. & Cona, M. I. (2018). Studying the quantity component of seed dispersal effectiveness from exclosure treatments and camera trapping. Ecology and Evolution 8, 5470–5479. 29938066 10.1002/ece3.4068 PMC6010695

[brv12809-bib-0043] Canale, G. R. , Suscke, P. , Rocha‐Santos, L. , Bernardo, C. S. S. , Kierulff, M. C. M. & Chivers, D. J. (2016). Seed dispersal of threatened tree species by a critically endangered primate in a Brazilian hotspot. Folia Primatologica 87, 123–140. 10.1159/000447712 27553249

[brv12809-bib-0044] Caputo, F. P. & Vogt, R. C. (2008). Stomach flushing vs. fecal analysis: the example of *Phrynops rufipes* (Testudines: Chelidae). Copeia 2, 301–305.

[brv12809-bib-0045] Cárdenas, S. , Echeverry‐Galvis, M. Á. & Stevenson, P. R. (2021). Seed dispersal effectiveness by oilbirds (*Steatornis caripensis*) in the southern Andes of Colombia. Biotropica 53, 671–680.

[brv12809-bib-0046] Carlo, T. A. , Tewksbury, J. J. & del Río, C. M. (2009). A new method to track seed dispersal and recruitment using 15N isotope enrichment. Ecology 90, 3516–3525. 20120818 10.1890/08-1313.1

[brv12809-bib-0047] Carvajal, A. & Adler, G. H. (2008). Seed dispersal and predation by *Proechimys semispinosus* and *Sciurus granatensis* in gaps and understorey in Central Panama. Journal of Tropical Ecology 24, 485–492.

[brv12809-bib-0048] * Carvalho, C. S. , Valverde, J. , Souza, M. , Ribeiro, T. , Nazareth, S. , Galetti, M. & Côrtes, M. C. (2019). El papel de los zorzales en el mantenimiento de la estructura y diversidad genética de una palma tropical. Ecosistemas: Revista Cietifica y Tecnica de Ecologia y Medio Ambiente 28, 26–34.

[brv12809-bib-0049] * Castro, E. R. , Galetti, M. & Morellato, L. P. C. (2007). Reproductive phenology of *Euterpe edulis* (Arecaceae) along a gradient in the Atlantic rainforest of Brazil. Australian Journal of Botany 55, 725–735.

[brv12809-bib-0050] Charles‐Dominique, P. , Chave, J. , Dubois, M. A. , De Granville, J. J. , Riera, B. & Vezzoli, C. (2003). Colonization front of the understorey palm *Astrocaryum sciophilum* in a pristine rain forest of French Guiana. Global Ecology and Biogeography 12, 237–248.

[brv12809-bib-0051] * Chaves, O. M. & Bicca‐Marques, J. C. (2016). Feeding strategies of brown howler monkeys in response to variations in food availability. PLoS One 11, e0145819. 26848959 10.1371/journal.pone.0145819 PMC4743924

[brv12809-bib-0052] Chaves, O. M. , Stoner, K. E. & Arroyo‐Rodríguez, V. (2012). Differences in diet between spider monkey groups living in forest fragments and continuous forest in Mexico. Biotropica 44, 105–113.

[brv12809-bib-0053] Choo, J. , Juenger, T. E. & Simpson, B. B. (2012). Consequences of frugivore‐mediated seed dispersal for the spatial and genetic structures of a Neotropical palm. Molecular Ecology 21, 1019–1031. 22229743 10.1111/j.1365-294X.2011.05425.x

[brv12809-bib-0054] * Cid, B. , Oliveira‐Santos, L. G. R. & Mourão, G. (2013). Seasonal habitat use of agoutis (*Dasyprocta azarae)* is driven by the palm *Attalea phalerata* in Brazilian Pantanal. Biotropica 45, 380–385.

[brv12809-bib-0055] * Cid, B. , Figueira, L. , de T. e Mello, A. F. , Pires, A. S. & Fernandez, F. A. (2014). Short‐term success in the reintroduction of the red‐humped agouti *Dasyprocta leporina*, an important seed disperser, in a Brazilian Atlantic Forest reserve. Tropical Conservation Science 7, 796–810.

[brv12809-bib-0056] Cintra, R. & Horna, V. (1997). Seed and seedling survival of the palm *Astrocaryum murumuru* and the legume tree *Dipteryx micrantha* in gaps in Amazonian forest. Journal of Tropical Ecology 13, 257–277.

[brv12809-bib-0057] Correa, S. B. , Costa‐Pereira, R. , Fleming, T. , Goulding, M. & Anderson, J. T. (2015). Neotropical fish–fruit interactions: eco‐evolutionary dynamics and conservation. Biological Reviews 90, 1263–1278. 25599800 10.1111/brv.12153

[brv12809-bib-0058] Correa, S. B. , Winemiller, K. O. , López‐Fernández, H. & Galetti, M. (2007). Evolutionary perspectives on seed consumption and dispersal by fishes. Bioscience 57, 748–756.

[brv12809-bib-0059] Côrtes, M. C. & Uriarte, M. (2013). Integrating frugivory and animal movement: a review of the evidence and implications for scaling seed dispersal. Biological Reviews 88, 255–272. 23136896 10.1111/j.1469-185X.2012.00250.x

[brv12809-bib-0060] Couvreur, T. L. & Baker, W. J. (2013). Tropical rain forest evolution: palms as a model group. BMC Biology 11, 1–4. 23587415 10.1186/1741-7007-11-48 PMC3627317

[brv12809-bib-0061] * de Almeida, L. B. & Galetti, M. (2007). Seed dispersal and spatial distribution of *Attalea geraensis* (Arecaceae) in two remnants of Cerrado in southeastern Brazil. Acta Oecologica 32, 180–187.

[brv12809-bib-0062] de Araújo, C. B. & Marcondes‐Machado, L. O. (2011). Diet and feeding behavior of the yellow‐faced parrot (*Alipiopsitta xanthops*) in Brasilia, Brazil. Ornitologia Neotropical 22, 79–88.

[brv12809-bib-0063] * de Arruda Nascimento, V. L. , de Souza, L. L. , Ferreira, J. A. , Tomas, W. M. , Borges, P. A. L. , Desbiez, A. & Takahasi, A. (2004). Utilização de frutos de acuri (Attalea phalerata Mart. ex Spreng) por cutias (Dasyprocta azarae) no Pantanal da Nhecolândia. *IV Simpósio sobre Recursos Naturais e Sócio‐econômicos do Pantanal*, pp. 1–7.

[brv12809-bib-0064] Diaz‐Martin, Z. & Karubian, J. (2021). Forest cover at landscape scales increases male and female gametic diversity of palm seedlings. Molecular Ecology 30, 4353–4367. 34216497 10.1111/mec.16060

[brv12809-bib-0065] * Dittel, J. W. , Lambert, T. D. & Adler, G. H. (2015). Seed dispersal by rodents in a lowland forest in Central Panama. Journal of Tropical Ecology 31, 403–412.

[brv12809-bib-0066] * Dobey, S. , Masters, D. V. , Scheick, B. K. , Clark, J. D. , Pelton, M. R. & Sunquist, M. E. (2005). Ecology of Florida black bears in the Okefenokee‐Osceola ecosystem. Wildlife Monographs 158, 1–41.

[brv12809-bib-0067] * Donatti, C. I. , Guimarães, P. R. & Galetti, M. (2009). Seed dispersal and predation in the endemic Atlantic rainforest palm *Astrocaryum aculeatissimum* across a gradient of seed disperser abundance. Ecological Research 24, 1187–1195.

[brv12809-bib-0068] * dos Santos, J. , Varassin, I. G. & Muschner, V. C. (2018a). Effects of neighborhood on pollination and seed dispersal of a threatened palm. Acta Oecologica 92, 95–101.

[brv12809-bib-0069] * dos Santos, J. , Varassin, I. G. & Muschner, V. C. (2018b). Survey dataset on mutualistic interactions among *Euterpe edulis* Mart.(Arecaceae) and floral and frugivorous visitors considering influence of neighborhood plant density and availability of resources. Data in Brief 21, 2015–2019. 30510986 10.1016/j.dib.2018.11.066 PMC6258885

[brv12809-bib-0070] Dracxler, C. M. & Forget, P.‐M. (2017). Seed caching by rodents favours seedling establishment of two palm species in a lowland Atlantic forest remnant. Journal of Tropical Ecology 33, 228–231.

[brv12809-bib-0071] Dracxler, C. M. & Kissling, W. D. (2021). Data from: the mutualism–antagonism continuum in Neotropical palm–frugivore interactions: from interaction outcomes to ecosystem dynamics. Dryad Dataset. 10.5061/dryad.6t1g1jwz5. PMC9297963 34725900

[brv12809-bib-0072] Dracxler, C. M. , Pires, A. S. & Fernandez, F. A. S. (2011). Invertebrate seed predators are not all the same: seed predation by bruchine and scolytine beetles affects palm recruitment in different ways. Biotropica 43, 8–11.

[brv12809-bib-0073] * Eguiarte, L. E. , Burquez, A. , Rodriguez, J. , Martínez‐Ramos, M. , Sarukhan, J. & Pinero, D. (1993). Direct and indirect estimates of neighborhood and effective population size in a tropical palm, *Astrocaryum mexicanum* . Evolution 47, 75–87. 28568093 10.1111/j.1558-5646.1993.tb01200.x

[brv12809-bib-0074] Eiserhardt, W. L. , Svenning, J. C. , Kissling, W. D. & Balslev, H. (2011). Geographical ecology of the palms (Arecaceae): determinants of diversity and distributions across spatial scales. Annals of Botany 108, 1391–1416. 21712297 10.1093/aob/mcr146 PMC3219491

[brv12809-bib-0075] Escobar, S. , Razafindratsima, O. H. , Montúfar, R. & Balslev, H. (2020). Post‐dispersal seed removal in a large‐seeded palm by frugivore mammals in Western Ecuador. Tropical Conservation Science 13, 1–14.

[brv12809-bib-0076] * Fadini, R. F. , Fleury, M. , Donatti, C. I. & Galetti, M. (2009). Effects of frugivore impoverishment and seed predators on the recruitment of a keystone palm. Acta Oecologica 35, 188–196.

[brv12809-bib-0077] Falcón, W. , Moll, D. & Hansen, D. M. (2020). Frugivory and seed dispersal by chelonians: a review and synthesis. Biological Reviews 95, 142–166. 10.1111/brv.12558 31608582

[brv12809-bib-0078] Fedriani, J. M. & Delibes, M. (2013). Pulp feeders alter plant interactions with subsequent animal associates. Journal of Ecology 101, 1581–1588.

[brv12809-bib-0079] Fedriani, J. M. , Zywiec, M. & Delibes, M. (2012). Thieves or mutualists? Pulp feeders enhance endozoochore local recruitment. Ecology 93, 575–587. 22624212 10.1890/11-0429.1

[brv12809-bib-0080] Feer, F. , Henry, O. , Forget, P.‐M. & Gayot, M. (2001). Frugivory and seed dispersal by terrestrial mammals. In Nouragues: Dynamics and Plant–Animal Interactions in a Neotropical Rainforest (eds F. Bongers , P. Charles‐Dominique , P.‐M. Forget and M. Théry ), pp. 227–232. Kluwer Academic, Dordrecht.

[brv12809-bib-0081] Filho, R. F. , Andrade, B. M. T. & Bezerra, B. (2021). Trash, tasty and healthy: the red‐back agouti (*Dasyprocta iacki*) feed on leftovers from blonde capuchins (*Sapajus flavius*). Tropical Ecology 62, 149–152.

[brv12809-bib-0082] Fleming, T. H. , Breitwisch, R. & Whitesides, G. H. (1987). Patterns of tropical vertebrate frugivore diversity. Annual Review of Ecology and Systematics 18, 91–109.

[brv12809-bib-0083] * Fleury, M. & Galetti, M. (2004). Effects of microhabitat on palm seed predation in two forest fragments in Southeast Brazil. Acta Oecologica 26, 179–184.

[brv12809-bib-0084] * Fleury, M. & Galetti, M. (2006). Forest fragment size and microhabitat effects on palm seed predation. Biological Conservation 131, 1–13.

[brv12809-bib-0085] * Forget, P.‐M. (1991). Scatterhoarding of *Astrocaryum paramaca* by *Proechimys* in French Guiana: comparison with *Myoprocta exilis* . Tropical Ecology 32, 155–167.

[brv12809-bib-0086] * Forget, P.‐M. , Munoz, E. & Leigh, E. G. Jr. (1994). Predation by rodents and bruchid beetles on seeds of *Scheelea* palms on Barro Colorado Island, Panama. Biotropica 26, 420–426.

[brv12809-bib-0087] Forget, P.‐M. & Wenny, D. (2005). How to elucidate seed fate? A review of methods used to study seed removal and secondary seed dispersal. In Seed Fate: Predation, Dispersal and Seedling Establishment (eds P.‐M. Forget , J. E. Lambert , P. E. Hulme and S. B. Vander Wall ), pp. 331–350. CABI Publishing, Wallingford.

[brv12809-bib-0088] Fragoso, J. M. V. (1997). Tapir‐generated seed shadows: scale‐dependent patchiness in the Amazon rain forest. Journal of Ecology 85, 519–529.

[brv12809-bib-0089] Fragoso, J. M. V. & Huffman, J. M. (2000). Seed‐dispersal and seedling recruitment patterns by the last Neotropical megafaunal element in Amazonia, the tapir. Journal of Tropical Ecology 16, 369–385.

[brv12809-bib-0090] Fragoso, J. M. V. , Silvius, K. M. & Correa, J. A. (2003). Long‐distance seed dispersal by tapirs increases seed survival and aggregates tropical trees. Ecology 84, 1998–2006.

[brv12809-bib-0091] Franco‐Quimbay, J. & Rojas‐Robles, R. (2015). Frugivoría y dispersión de semillas de la palma *Oenocarpus bataua* en dos regiones con diferente estado de conservación. Actualidades Biológicas 37, 273–285.

[brv12809-bib-0092] Fricke, E. C. , Bender, J. , Rehm, E. M. & Rogers, H. S. (2019). Functional outcomes of mutualistic network interactions: a community‐scale study of frugivore gut passage on germination. Journal of Ecology 107, 757–767.

[brv12809-bib-0093] Fuzessy, L. F. , Cornelissen, T. G. , Janson, C. & Silveira, F. A. O. (2016). How do primates affect seed germination? A meta‐analysis of gut passage effects on neotropical plants. Oikos 125, 1069–1080.

[brv12809-bib-0094] Fuzessy, L. F. , Janson, C. & Silveira, F. A. O. (2018). Effects of seed size and frugivory degree on dispersal by Neotropical frugivores. Acta Oecologica 93, 41–47.

[brv12809-bib-0095] * Galetti, M. & Aleixo, A. (1998). Effects of palm heart harvesting on avian frugivores in the Atlantic rain forest of Brazil. Journal of Applied Ecology 35, 286–293.

[brv12809-bib-0096] * Galetti, M. , Donatti, C. I. , Pizo, M. A. & Giacomini, H. C. (2008). Big fish are the best: seed dispersal of *Bactris glaucescens* by the pacu fish (*Piaractus mesopotamicus*) in the Pantanal, Brazil. Biotropica 40, 386–389.

[brv12809-bib-0097] Galetti, M. , Donatti, C. I. , Steffler, C. , Genini, J. , Bovendorp, R. S. & Fleury, M. (2010). The role of seed mass on the caching decision by agoutis, *Dasyprocta leporina* (Rodentia: Agoutidae). Zoologia 27, 472–476.

[brv12809-bib-0098] Galetti, M. , Guevara, R. , Côrtes, M. C. , Fadini, R. , Von Matter, S. , Leite, A. B. , Labecca, F. , Ribeiro, T. , Carvalho, C. S. , Collevatti, R. G. , Pires, M. M. , Guimarães, P. R. Jr. , Brancalion, P. H. , Ribeiro, M. C. & Jordano, P. (2013). Functional extinction of birds drives rapid evolutionary changes in seed size. Science 340, 1086–1090. 23723235 10.1126/science.1233774

[brv12809-bib-0099] * Galetti, M. , Keuroghlian, A. , Hanada, L. & Morato, M. I. (2001). Frugivory and seed dispersal by the lowland tapir (*Tapirus terrestris*) in southeast Brazil1. Biotropica 33, 723–726.

[brv12809-bib-0100] * Galetti, M. , Laps, R. & Pizo, M. A. (2000). Frugivory by toucans (Ramphastidae) at two altitudes in the Atlantic Forest of Brazil. Biotropica 32, 842–850.

[brv12809-bib-0101] * Galetti, M. , Martuscelli, P. , Olmos, F. & Aleixo, A. (1997). Ecology and conservation of the Jacutinga *Pipile jacutinga* in the Atlantic forest of Brazil. Biological Conservation 82, 31–39.

[brv12809-bib-0102] * Galetti, M. , Paschoal, M. & Pedroni, F. (1992). Predation on palm nuts (*Syagrus romanzoffiana*) by squirrels (*Sciurus ingrami*) in south‐East Brazil. Journal of Tropical Ecology 8, 121–123.

[brv12809-bib-0103] * Gálvez, D. & Jansen, P. A. (2007). Bruchid beetle infestation and the value of *Attalea butyracea* endocarps for neotropical rodents. Journal of Tropical Ecology 23, 381–384.

[brv12809-bib-0104] Gálvez, D. , Kranstauber, B. , Kays, R. W. & Jansen, P. A. (2009). Scatter hoarding by the central American agouti: a test of optimal cache spacing theory. Animal Behaviour 78, 1327–1333.

[brv12809-bib-0105] * Garzon‐Lopez, C. X. , Ballesteros‐Mejia, L. , Ordoñez, A. , Bohlman, S. A. , Olff, H. & Jansen, P. A. (2015). Indirect interactions among tropical tree species through shared rodent seed predators: a novel mechanism of tree species coexistence. Ecology Letters 18, 752–760. 25939379 10.1111/ele.12452

[brv12809-bib-0106] Gatti, A. , Bianchi, R. , Rosa, C. R. X. & Mendes, S. L. (2006). Diet of two sympatric carnivores, *Cerdocyon thous* and *Procyon cancrivorus*, in a Restinga area of Espirito Santo state, Brazil. Journal of Tropical Ecology 22, 227–230.

[brv12809-bib-0107] Gautier‐Hion, A. , Duplantier, J. M. , Quris, R. , Feer, F. , Sourd, C. , Decoux, J. P. , Dubost, G. , Emmons, L. , Erard, C. , Hecketsweiler, P. , Moungazi, A. , Roussilhon, C. & Thiollay, J. M. (1985). Fruit characters as a basis of fruit choice and seed dispersal in a tropical forest vertebrate community. Oecologia 65, 324–337. 28310436 10.1007/BF00378906

[brv12809-bib-0108] Genrich, C. M. , Mello, M. A. , Silveira, F. A. , Bronstein, J. L. & Paglia, A. P. (2017). Duality of interaction outcomes in a plant–frugivore multilayer network. Oikos 126, 361–368.

[brv12809-bib-0109] Giombini, M. I. , Bravo, S. P. & Martinez, M. F. (2009). Seed dispersal of the palm *Syagrus romanzoffiana* by tapirs in the semi‐deciduous Atlantic Forest of Argentina. Biotropica 41, 408–413.

[brv12809-bib-0110] Giombini, M. I. , Bravo, S. P. & Tosto, D. S. (2016). The key role of the largest extant Neotropical frugivore (*Tapirus terrestris*) in promoting admixture of plant genotypes across the landscape. Biotropica 48, 499–508.

[brv12809-bib-0111] Giombini, M. I. , Bravo, S. P. , Sica, Y. V. & Tosto, D. S. (2017). Early genetic consequences of defaunation in a large‐seeded vertebrate‐dispersed palm (*Syagrus romanzoffiana*). Heredity 118, 568–577. 28121308 10.1038/hdy.2016.130 PMC5436022

[brv12809-bib-0112] * Glanz, W. E. , Thorington, R. W. , Giacalone‐Madden, J. & Heaney, L. R. (1982). Seasonal food use and demographic trends in *Sciurus granatensis* . In The Ecology of a Tropical Forest: Seasonal Rhythms and Long‐Term Changes (eds E. G. Leigh , A. S. Rand and D. M. Windsor ), pp. 239–252. Smithsonian Institution Press, Washington.

[brv12809-bib-0113] * Göldel, B. , Araujo, A. C. , Kissling, W. D. & Svenning, J. C. (2016). Impacts of large herbivores on spinescence and abundance of palms in the Pantanal, Brazil. Botanical Journal of the Linnean Society 182, 465–479.

[brv12809-bib-0114] Gómez, J. M. , Schupp, E. W. & Jordano, P. (2019). Synzoochory: the ecological and evolutionary relevance of a dual interaction. Biological Reviews 94, 874–902. 30467946 10.1111/brv.12481

[brv12809-bib-0115] González‐Varo, J. P. , Arroyo, J. M. & Jordano, P. (2014). Who dispersed the seeds? The use of DNA barcoding in frugivory and seed dispersal studies. Methods in Ecology and Evolution 5, 806–814.

[brv12809-bib-0116] Grenha, V. , Macedo, M. V. , Pires, A. S. & Monteiro, R. F. (2010). The role of *Cerradomys subflavus* (Rodentia, Cricetidae) as seed predator and disperser of the palm *Allagoptera arenaria* . Mastozoología Neotropical 17, 61–68.

[brv12809-bib-0117] Guimarães, P. R. Jr. (2020). The structure of ecological networks across levels of organization. Annual Review of Ecology, Evolution, and Systematics 51, 433–460.

[brv12809-bib-0118] * Guix, J. C. & Ruiz, X. (1995). Toucans and thrushes as potential dispersers of seed‐predatory weevil larvae in southeastern Brazil. Canadian Journal of Zoology 73, 745–748.

[brv12809-bib-0119] * Guix, J. C. & Ruiz, X. (1997). Weevil larvae dispersal by guans in southeastern Brazil. Biotropica 29, 522–525.

[brv12809-bib-0120] * Guix, J. C. & Ruiz, X. (2000). Plant‐disperser‐pest evolutionary triads: how widespread are they? Orsis 15, 121–126.

[brv12809-bib-0121] * Guix, J. C. , Ruiz, X. & Jover, L. (2001). Resource partitioning and interspecific competition among coexisting species of guans and toucans in SE Brazil. Netherlands Journal of Zoology 51, 285–297.

[brv12809-bib-0122] Henderson, A. (2002). Evolution and Ecology of Palms. Bronx, NY: New York Botanical Garden Press.

[brv12809-bib-0123] * Hernandez, A. (2011). Internal dispersal of seed‐inhabiting insects by vertebrate frugivores: a review and prospects. Integrative Zoology 6, 213–221. 21910840 10.1111/j.1749-4877.2011.00245.x

[brv12809-bib-0124] Herrera, C. M. (1982). Defense of ripe fruit from pests: its significance in relation to plant‐disperser interactions. The American Naturalist 120, 218–241.

[brv12809-bib-0125] Hibert, F. , Sabatier, D. , Andrivot, J. , Scotti‐Saintagne, C. , Gonzalez, S. , Prévost, M. F. , Grenand, P. , Chave, J. , Caron, H. & Richard‐Hansen, C. (2011). Botany, genetics and ethnobotany: a crossed investigation on the elusive tapir's diet in French Guiana. PLoS One 6, e25850. 21991372 10.1371/journal.pone.0025850 PMC3185057

[brv12809-bib-0126] Hirsch, B. T. , Kays, R. & Jansen, P. A. (2012). A telemetric thread tag for tracking seed dispersal by scatter‐hoarding rodents. Plant Ecology 213, 933–943.

[brv12809-bib-0127] * Hirsch, B. T. , Kays, R. & Jansen, P. A. (2013). Evidence for cache surveillance by a scatter‐hoarding rodent. Animal Behaviour 85, 1511–1516.

[brv12809-bib-0128] Hirsch, B. T. , Kays, R. , Pereira, V. E. & Jansen, P. A. (2012). Directed seed dispersal towards areas with low conspecific tree density by a scatter‐hoarding rodent. Ecology Letters 15, 1423–1429. 22958079 10.1111/ele.12000

[brv12809-bib-0129] Hoch, G. A. & Adler, G. H. (1997). Removal of black palm (*Astrocaryum standleyanum*) seeds by spiny rats (*Proechimys semispinosus*). Journal of Tropical Ecology 13, 51–58.

[brv12809-bib-0130] Howe, H. F. & Smallwood, J. (1982). Ecology of seed dispersal. Annual Review of Ecology and Systematics 13, 201–228.

[brv12809-bib-0131] Hulme, P. E. (1994). Post‐dispersal seed predation in grassland: its magnitude and sources of variation. Journal of Ecology 82, 645–652.

[brv12809-bib-0132] Hulme, P. E. (2002). Seed‐eaters: seed dispersal, destruction and demography. In Seed Dispersal and Frugivory: Ecology, Evolution, and Conservation (eds D. J. Levey , W. R. Silva and M. Galetti ), pp. 257–274. CABI Publishing, Wallingford.

[brv12809-bib-0133] Hulme, P. E. & Benkman, C. W. (2002). Granivory. In Plant‐Animal Interactions: An Evolutionary Approach (eds C. M. Herrera and O. Pellmyr ), pp. 132–156. Oxford University Press, Oxford.

[brv12809-bib-0134] * Izawa, K. & Mizuno, A. (1977). Palm‐fruit cracking behavior of wild black‐capped capuchin (*Cebus apella*). Primates 18, 773–792.

[brv12809-bib-0135] Jansen, P. A. , Bartholomeus, M. , Bongers, F. , Elzinga, J. A. , Den Ouden, J. & Van Wieren, S. E. (2002). The role of seed size in dispersal by a scatterhoarding rodent. In Seed Dispersal and Frugivory: Ecology, Evolution and Conservation (eds D. J. Levey , W. R. Silva and M. Galetti ), pp. 209–225. CAB International, Wallingford.

[brv12809-bib-0136] Jansen, P. A. , Bongers, F. & Hemerik, L. (2004). Seed mass and mast seeding enhance dispersal by a neotropical scatter‐hoarding rodent. Ecological Monographs 74, 569–589.

[brv12809-bib-0137] * Jansen, P. A. , Elschot, K. , Verkerk, P. J. & Wright, S. J. (2010). Seed predation and defleshing in the agouti‐dispersed palm *Astrocaryum standleyanum* . Journal of Tropical Ecology 26, 473–480.

[brv12809-bib-0138] Jansen, P. A. , Hirsch, B. T. , Emsens, W.‐J. , Zamora‐Gutierrez, V. , Wikelski, M. & Kays, R. (2012). Thieving rodents as substitute dispersers of megafaunal seeds. Proceedings of the National Academy of Sciences of the United States of America 109, 12610–12615. 22802644 10.1073/pnas.1205184109 PMC3412018

[brv12809-bib-0139] * Jansen, P. A. , Visser, M. D. , Joseph Wright, S. , Rutten, G. & Muller‐Landau, H. C. (2014). Negative density dependence of seed dispersal and seedling recruitment in a Neotropical palm. Ecology Letters 17, 1111–1120. 25039608 10.1111/ele.12317

[brv12809-bib-0140] Janzen, D. H. (1971). Seed predation by animals. Annual Review of Ecology and Systematics 2, 465–492.

[brv12809-bib-0141] * Jerozolimski, A. , Ribeiro, M. B. N. & Martins, M. (2009). Are tortoises important seed dispersers in Amazonian forests? Oecologia 161, 517–528. 19575239 10.1007/s00442-009-1396-8

[brv12809-bib-0142] Jordano, P. (1987). Patterns of mutualistic interactions in pollination and seed dispersal: connectance, dependence asymmetries, and coevolution. The American Naturalist 129, 657–677.

[brv12809-bib-0143] Jorge, M. L. S. P. & Howe, H. F. (2009). Can forest fragmentation disrupt a conditional mutualism? A case from Central Amazon. Oecologia 161, 709–718. 19633870 10.1007/s00442-009-1417-7

[brv12809-bib-0144] Junges, S. D. O. , Consatti, G. , Périco, E. , Bordignon, S. A. D. L. , Freitas, E. M. D. & Cademartori, C. V. (2018). Endozoochory by *Didelphis albiventris* Lund, 1840 (Mammalia, Didelphimorphia) in a semideciduous seasonal forest remnant in the south of Brazil. Biota Neotropica 18, 20170389.

[brv12809-bib-0145] Karubian, J. , Browne, L. , Cabrera, D. , Chambers, M. & Olivo, J. (2016). Relative influence of relatedness, conspecific density and microhabitat on seedling survival and growth of an animal‐dispersed Neotropical palm, *Oenocarpus bataua* . Botanical Journal of the Linnean Society 182, 425–438.

[brv12809-bib-0146] Karubian, J. , Duraes, R. , Storey, J. L. & Smith, T. B. (2012). Mating behavior drives seed dispersal by the long‐wattled umbrellabird *Cephalopterus penduliger* . Biotropica 44, 689–698.

[brv12809-bib-0147] Karubian, J. , Ottewell, K. , Link, A. & Di Fiore, A. (2015). Genetic consequences of seed dispersal to sleeping trees by white‐bellied spider monkeys. Acta Oecologica 68, 50–58.

[brv12809-bib-0148] Karubian, J. , Sork, V. L. , Roorda, T. , Duraes, R. & Smith, T. B. (2010). Destination‐based seed dispersal homogenizes genetic structure of a tropical palm. Molecular Ecology 19, 1745–1753. 20345676 10.1111/j.1365-294X.2010.04600.x

[brv12809-bib-0149] Kays, R. W. (1999). Food preferences of kinkajous (*Potos flavus*): a frugivorous carnivore. Journal of Mammalogy 80, 589–599.

[brv12809-bib-0150] * Keuroghlian, A. & Eaton, D. P. (2008). Fruit availability and peccary frugivory in an isolated Atlantic forest fragment: effects on peccary ranging behavior and habitat use. Biotropica 40, 62–70.

[brv12809-bib-0151] Keuroghlian, A. & Eaton, D. P. (2009). Removal of palm fruits and ecosystem engineering in palm stands by white‐lipped peccaries (*Tayassu pecari*) and other frugivores in an isolated Atlantic Forest fragment. Biodiversity and Conservation 18, 1733–1750.

[brv12809-bib-0152] Kiltie, R. A. (1981). Distribution of palm fruits on a rain forest floor: why white‐lipped peccaries forage near objects. Biotropica 13, 141–145.

[brv12809-bib-0153] Kissling, W. D. , Balslev, H. , Baker, W. J. , Dransfield, J. , Göldel, B. , Lim, J. Y. , Onstein, R. E. & Svenning, J. C. (2019). PalmTraits 1.0, a species‐level functional trait database of palms worldwide. Scientific Data 6, 1–13. 31551423 10.1038/s41597-019-0189-0 PMC6760217

[brv12809-bib-0154] Kissling, W. D. , Böhning‐Gaese, K. & Jetz, W. (2009). The global distribution of frugivory in birds. Global Ecology and Biogeography 18, 150–162.

[brv12809-bib-0155] Kissling, W. D. , Eiserhardt, W. L. , Baker, W. J. , Borchsenius, F. , Couvreur, T. L. , Balslev, H. & Svenning, J. C. (2012). Cenozoic imprints on the phylogenetic structure of palm species assemblages worldwide. Proceedings of the National Academy of Sciences 109, 7379–7384. 10.1073/pnas.1120467109 PMC3358898 22529387

[brv12809-bib-0156] Klinger, R. & Rejmánek, M. (2010). A strong conditional mutualism limits and enhances seed dispersal and germination of a tropical palm. Oecologia 162, 951–963. 20049480 10.1007/s00442-009-1542-3 PMC2841266

[brv12809-bib-0157] * Kuprewicz, E. K. (2013). Mammal abundances and seed traits control the seed dispersal and predation roles of terrestrial mammals in a Costa Rican forest. Biotropica 45, 333–342.

[brv12809-bib-0158] Kuprewicz, E. K. (2015). Scatter hoarding of seeds confers survival advantages and disadvantages to large‐seeded tropical plants at different life stages. PLoS One 10, e0124932. 25970832 10.1371/journal.pone.0124932 PMC4430353

[brv12809-bib-0159] * Lambert, T. D. , Sumpter, K. L. , Dittel, J. W. , Dupre, S. , Casanova, K. , Winker, A. & Adler, G. H. (2014). Roads as barriers to seed dispersal by small mammals in a neotropical forest. Tropical Ecology 55, 263–269.

[brv12809-bib-0160] Lamperty, T. , Karubian, J. & Dunham, A. E. (2021). Ecological drivers of intraspecific variation in seed dispersal services of a common neotropical palm. Biotropica 53, 1226–1237.

[brv12809-bib-0161] Levey, D. J. (1987). Seed size and fruit‐handling techniques of avian frugivores. The American Naturalist 129, 471–485.

[brv12809-bib-0162] Lichti, N. I. , Steele, M. A. & Swihart, R. K. (2017). Seed fate and decision‐making processes in scatter‐hoarding rodents. Biological Reviews 92, 474–504. 26587693 10.1111/brv.12240

[brv12809-bib-0163] Link, A. & De Luna, A. G. (2004). The importance of *Oenocarpus bataua* (Arecaceae). In the diet of spider monkeys at Tinigua National Park, Colombia. Folia Primatologica 75, 391–391.

[brv12809-bib-0164] Link, A. & Di Fiore, A. (2006). Seed dispersal by spider monkeys and its importance in the maintenance of neotropical rain‐forest diversity. Journal of Tropical Ecology 22, 235–246.

[brv12809-bib-0165] Liu, G. , Platt, S. G. & Borg, C. K. (2004). Seed dispersal by the Florida box turtle (*Terrapene carolina bauri*) in pine Rockland forests of the lower Florida keys, United States. Oecologia 138, 539–546. 14685845 10.1007/s00442-003-1445-7

[brv12809-bib-0166] Loayza, A. P. & Knight, T. (2010). Seed dispersal by pulp consumers, not “legitimate” seed dispersers, increases *Guettarda viburnoides* population growth. Ecology 91, 2684–2695. 20957962 10.1890/09-0480.1

[brv12809-bib-0167] Lopez‐Toledo, L. , Portillo‐Cruz, Y. , Pulido, M. T. & Endress, B. A. (2013). Seed dynamics of an endemic palm in a northwestern Mexican tropical dry forest: implications for population spatial structure. Plant Ecology 214, 1115–1125.

[brv12809-bib-0168] * Mahoney, M. C. , Browne, L. , Diaz‐Martin, Z. , Olivo, J. , Cabrera, J. , Gonzalez, M. , Hazlehurst, J. & Karubian, J. (2018). Fruit removal by large avian frugivores varies in relation to habitat quality in continuous Neotropical rainforest. Ornitologia Neotropical 29, 247–254.

[brv12809-bib-0169] * Martínez‐Gallardo, R. & Sánchez‐Cordero, V. (1993). Dietary value of fruits and seeds to spiny pocket mice, *Heteromys desmarestianus* (Heteromyidae). Journal of Mammalogy 74, 436–442.

[brv12809-bib-0170] Matos, D. M. S. & Watkinson, A. R. (1998). The fecundity, seed, and seedling ecology of the edible palm *Euterpe edulis* in southeastern Brazil. Biotropica 30, 595–603.

[brv12809-bib-0171] Meiga, A. Y. Y. & Christianini, A. V. (2020). Potential impact of mammal defaunation on the early regeneration of a large‐seeded palm in the Brazilian Atlantic Forest. Neotropical Biology & Conservation 15, 177–193.

[brv12809-bib-0172] Mendes, C. P. & Cândido‐Jr, J. F. (2014). Behavior and foraging technique of the Ingram's squirrel *Guerlinguetus ingrami* (Sciuridae: Rodentia) in an araucaria moist forest fragment. Zoologia 31, 209–214.

[brv12809-bib-0173] Mendes, C. P. , Koprowski, J. L. & Galetti, M. (2019). NEOSQUIRREL: a data set of ecological knowledge on Neotropical squirrels. Mammal Review 49, 210–225.

[brv12809-bib-0174] * Mendieta‐Aguilar, G. , Pacheco, L. F. & Roldán, A. I. (2015). Dispersión de semillas de *Mauritia flexuosa* (Arecaceae) por frugívoros terrestres en Laguna Azul, Beni, Bolivia. Acta Amazonica 45, 45–56.

[brv12809-bib-0175] Menezes, L. F. T. , Pugnaire, F. I. , Matallana, G. , Nettesheim, F. C. , Carvalho, D. C. & Mattos, E. A. (2018). Disentangling plant establishment in sandy coastal systems: biotic and abiotic factors that determine *Allagoptera arenaria* (Arecaceae) germination. Acta Botanica Brasilica 32, 12–19.

[brv12809-bib-0176] * Mengardo, A. L. & Pivello, V. R. (2012). Phenology and fruit traits of *Archontophoenix cunninghamiana*, an invasive palm tree in the Atlantic forest of Brazil. Ecotropica 18, 45–54.

[brv12809-bib-0177] Mittelman, P. U. , Dracxler, C. M. , Santos‐Coutinho, P. R. O. & Pires, A. S. (2021). Sowing forests: a synthesis of agouti (*Dasyprocta* spp.) seed dispersal and predation and their influence on plant communities. Biological Reviews. 10.1111/brv.12761. 34156131

[brv12809-bib-0178] Moegenburg, S. M. & Levey, D. J. (2003). Do frugivores respond to fruit harvest? An experimental study of short‐term responses. Ecology 84, 2600–2612.

[brv12809-bib-0179] Moher, D. , Liberati, A. , Tetzlaff, J. , Altman, D. G. & Prisma Group (2009). Preferred reporting items for systematic reviews and meta‐analyses: the PRISMA statement. PLoS Medicine 6, e1000097. 19621072 10.1371/journal.pmed.1000097 PMC2707599

[brv12809-bib-0180] Montesinos‐Navarro, A. , Hiraldo, F. , Tella, J. L. & Blanco, G. (2017). Network structure embracing mutualism–antagonism continuums increases community robustness. Nature Ecology & Evolution 1, 1661–1669. 28970589 10.1038/s41559-017-0320-6

[brv12809-bib-0181] * Montes‐Sánchez, J. J. , Huato‐Soberanis, L. , Buntinx‐Dios, S. E. & León‐de La Luz, J. L. (2020). The feral pig in a low impacted ecosystem: analysis of diet composition and its utility. Rangeland Ecology & Management 73, 703–711.

[brv12809-bib-0182] * Morini, M. S. C. , da Silva, R. R. & Kato, L. M. (2003). Non‐specific interaction between ants (hymenoptera; Formicidae) and fruits of *Syagrus romanzoffiana* (Arecaceae) in an area of the Brazilian Atlantic Forest. Sociobiology 42, 663–673.

[brv12809-bib-0183] Muñoz, G. , Trojelsgaard, K. & Kissling, W. D. (2019). A synthesis of animal‐mediated seed dispersal of palms reveals distinct biogeographical differences in species interactions. Journal of Biogeography 46, 466–484.

[brv12809-bib-0184] Muscarella, R. , Emilio, T. , Phillips, O. L. , Lewis, S. L. , Slik, F. , Baker, W. J. , Couvreur, T. L. P. , Eiserhardt, W. L. , Svenning, J.‐C. , Affum‐Baffoe, K. , Aiba, S.‐I. , Almeida, E. C. , Almeida, S. S. , Oliveira, E. A. , Álvarez‐Dávila, E. , et al. (2020). The global abundance of tree palms. Global Ecology and Biogeography 29, 1495–1514.

[brv12809-bib-0185] Nascimento, L. F. D. , Guimarães, P. R. Jr. , Onstein, R. E. , Kissling, W. D. & Pires, M. M. (2020). Associated evolution of fruit size, fruit colour and spines in Neotropical palms. Journal of Evolutionary Biology 33, 858–868. 32198956 10.1111/jeb.13619

[brv12809-bib-0186] Nathan, R. (2006). Long‐distance dispersal of plants. Science 313, 786–788. 16902126 10.1126/science.1124975

[brv12809-bib-0187] * O'Farrill, G. , Galetti, M. & Campos‐Arceiz, A. (2013). Frugivory and seed dispersal by tapirs: an insight on their ecological role. Integrative Zoology 8, 4–17. 23586556 10.1111/j.1749-4877.2012.00316.x

[brv12809-bib-0188] * Olmos, F. , Pardini, R. , Boulhosa, R. L. P. , Bürgi, R. & Morsello, C. (1999). Do tapirs steal food from palm seed predators or give them a lift? Biotropica 31, 375–379.

[brv12809-bib-0189] Onstein, R. E. , Baker, W. J. , Couvreur, T. L. , Faurby, S. , Svenning, J. C. & Kissling, W. D. (2017). Frugivory‐related traits promote speciation of tropical palms. Nature Ecology & Evolution 1, 1903–1911. 29062122 10.1038/s41559-017-0348-7

[brv12809-bib-0190] Ottewell, K. , Browne, L. , Cabrera, D. , Olivo, J. & Karubian, J. (2018). Genetic diversity of dispersed seeds is highly variable among leks of the long‐wattled umbrellabird. Acta Oecologica 86, 31–37.

[brv12809-bib-0191] * Palacios, E. , Rodriguez, A. & Defler, T. R. (1997). Diet of a group of *Callicebus torquatus lugens* (Humboldt, 1812) during the annual resource bottleneck in Amazonian Colombia. International Journal of Primatology 18, 503–522.

[brv12809-bib-0192] * Paschoal, M. & Galetti, M. (1995). Seasonal food use by the neotropical squirrel *Sciurus ingrami* in southeastern Brazil. Biotropica 27, 268–273.

[brv12809-bib-0193] Pedroso, P. M. , Mariano, V. , Kimura, M. G. & Christianini, A. V. (2021). Drought changes fruiting phenology, but does not affect seed predation of a keystone palm. Flora 283, 151917.

[brv12809-bib-0194] Peguero, G. , Muller‐Landau, H. C. , Jansen, P. A. & Wright, S. J. (2017). Cascading effects of defaunation on the coexistence of two specialized insect seed predators. Journal of Animal Ecology 86, 136–146. 27611694 10.1111/1365-2656.12590

[brv12809-bib-0195] * Peres, C. A. (1994). Composition, density, and fruiting phenology of arborescent palms in an Amazonian terra firme forest. Biotropica 26, 285–294.

[brv12809-bib-0196] * Peris, J. E. , Malara, T. M. , Borges, R. , Falconi, J. R. , Peña, L. & Fedriani, J. M. (2019). Reunion overseas: introduced wild boars and cultivated orange trees interact in the Brazilian Atlantic forest. Revista de Biología Tropical 67, 901–912.

[brv12809-bib-0197] Piedade, M. T. F. , Parolin, P. & Junk, W. (2006). Phenology, fruit production and seed dispersal of *Astrocaryum jauari* (Arecaceae) in Amazonian black water floodplains. Revista de Biología Tropical 54, 1171–1178. 18457155

[brv12809-bib-0198] Pimentel, D. S. & Tabarelli, M. (2004). Seed dispersal of the palm *Attalea oleifera* in a remnant of the Brazilian Atlantic Forest. Biotropica 36, 74–84.

[brv12809-bib-0199] * Piñero, D. , Martinez‐Ramos, M. & Sarukhán, J. (1984). A population model of Astrocaryum mexicanum and a sensitivity analysis of its finite rate of increase. Journal of Ecology 72, 977–991.

[brv12809-bib-0200] * Pinto, S. R. R. , Santos, A. M. M. & Tabarelli, M. (2009). Seed predation by rodents and safe sites for large‐seeded trees in a fragment of the Brazilian Atlantic forest. Brazilian Journal of Biology 69, 763–771. 10.1590/s1519-69842009000400003 19802435

[brv12809-bib-0201] Pires, A. S. & Galetti, M. (2012). The agouti *Dasyprocta leporina* (Rodentia: Dasyproctidae) as seed disperser of the palm *Astrocaryum aculeatissimum* . Mastozoología Neotropical 19, 147–153.

[brv12809-bib-0202] Pires, M. M. , Guimarães, P. R. Jr. , Galetti, M. & Jordano, P. (2018). Pleistocene megafaunal extinctions and the functional loss of long‐distance seed‐dispersal services. Ecography 41, 153–163.

[brv12809-bib-0203] * Pizo, M. A. & Almeida‐Neto, M. (2009). Determinants of fruit removal in *Geonoma pauciflora*, an understory palm of neotropical forests. Ecological Research 24, 1179–1186.

[brv12809-bib-0204] Pizo, M. A. & Simão, I. (2001). Seed deposition patterns and the survival of seeds and seedlings of the palm *Euterpe edulis* . Acta Oecologica 22, 229–233.

[brv12809-bib-0205] Pizo, M. A. , Von Allmen, C. & Morellato, L. P. C. (2006). Seed size variation in the palm *Euterpe edulis* and the effects of seed predators on germination and seedling survival. Acta Oecologica 29, 311–315.

[brv12809-bib-0206] * Puechagut, P. B. , Politi, N. , Bellis, L. M. & Rivera, L. O. (2013). A disappearing oasis in the semi‐arid Chaco: deficient palm regeneration and establishment. Journal for Nature Conservation 21, 31–36.

[brv12809-bib-0207] Quintela, F. M. , Iob, G. & Artioli, L. G. S. (2014). Diet of *Procyon cancrivorus* (Carnivora, Procyonidae) in Restinga and estuarine environments of southern Brazil. Iheringia Serie Zoologia 104, 143–149.

[brv12809-bib-0208] Quiroga‐Castro, V. D. & Roldán, A. I. (2001). The fate of *Attalea phalerata* (Palmae) seeds dispersed to a tapir latrine. Biotropica 33, 472–477.

[brv12809-bib-0209] Ragusa‐Netto, J. (2016). Nut density and removal in *Syagrus loefgrenii* glassman (Arecaceae) in the Brazilian Cerrado. Brazilian Journal of Biology 76, 726–734. 10.1590/1519-6984.02715 27097083

[brv12809-bib-0210] Ragusa‐Netto, J. (2018). The effect of size and density on nut removal in *Syagrus loefgrenii* glassman (Arecaceae) in the Brazilian Cerrado. Brazilian Journal of Biology 78, 147–154. 10.1590/1519-6984.166617 28699969

[brv12809-bib-0211] * Ramírez, B. H. , Parrado‐Rosselli, Á. & Stevenson, P. (2009). Seed dispersal of a useful palm (*Astrocaryum chambira* Burret) in three Amazonian forests with different human intervention. Colombia Forestal 12, 5–16.

[brv12809-bib-0212] * Ramos, S. L. F. , Dequigiovanni, G. , Sebbenn, A. M. , Lopes, M. T. G. , Kageyama, P. Y. , de Macêdo, J. L. V. , Kirst, M. & Veasey, E. A. (2016). Spatial genetic structure, genetic diversity and pollen dispersal in a harvested population of *Astrocaryum aculeatum* in the Brazilian Amazon. BMC Genetics 17, 1–10. 27108235 10.1186/s12863-016-0371-8 PMC4842287

[brv12809-bib-0213] * Ribeiro, L. F. , Conde, L. O. M. & Tabarelli, M. (2010). Predação e remoção de sementes de cinco espécies de palmeiras por *Guerlinguetus ingrami* (Thomas, 1901) em um fragmento urbano de floresta Atlântica montana. Revista Árvore 34, 637–649.

[brv12809-bib-0214] Rios, R. S. & Pacheco, L. F. (2006). The effect of dung and dispersal on postdispersal seed predation of *Attalea phalerata* (Arecaceae) by bruchid beetles. Biotropica 38, 778–781.

[brv12809-bib-0215] Robertson, A. W. , Trass, A. , Ladley, J. J. & Kelly, D. (2006). Assessing the benefits of frugivory for seed germination: the importance of the deinhibition effect. Functional Ecology 20, 58–66.

[brv12809-bib-0216] * Rocha, V. J. , Reis, N. R. D. & Sekiama, M. L. (1998). Uso de ferramentas por *Cebus apella* (Linnaeus)(Primates, Cebidae) para obtenção de larvas de coleóptera que parasitam sementes de *Syagrus romanzoffianum* (Cham.) Glassm.(Arecaceae). Revista Brasileira de Zoologia 15, 945–950.

[brv12809-bib-0217] Rodrigues, M. , Olmos, F. & Galetti, M. (1993). Seed dispersal by tapir in southeastern Brazil. Mammalia 57, 460–461.

[brv12809-bib-0218] Rodríguez‐Rodríguez, M. C. , Jordano, P. & Valido, A. (2017). Functional consequences of plant‐animal interactions along the mutualism–antagonism gradient. Ecology 98, 1266–1276. 28135774 10.1002/ecy.1756

[brv12809-bib-0219] * Rojas‐Robles, R. , Gary Stiles, F. & Muñoz‐Saba, Y. (2012). Frugivory and seed dispersal *Oenocarpus bataua* palm (Arecaceae) in a forest from the Colombian Andes. Revista de Biologia Tropical 60, 1445–1461. 23342501

[brv12809-bib-0220] * Roldán, A. I. & Simonetti, J. A. (2001). Plant‐mammal interactions in tropical Bolivian forests with different hunting pressures. Conservation Biology 15, 617–623.

[brv12809-bib-0221] * Roman, C. , Neto, L. T. & Cáceres, N. C. (2010). Fruit manipulation of the palm *Syagrus romanzoffiana* by vertebrates in southern Brazil. Neotropical Biology and Conservation 5, 101–105.

[brv12809-bib-0222] * Russo, S. E. , Portnoy, S. & Augspurger, C. K. (2006). Incorporating animal behavior into seed dispersal models: implications for seed shadows. Ecology 87, 3160–3174. 17249240 10.1890/0012-9658(2006)87[3160:iabisd]2.0.co;2

[brv12809-bib-0223] Sales, L. P. , Kissling, W. D. , Galetti, M. , Naimi, B. & Pires, M. M. (2021). Climate change reshapes the eco‐evolutionary dynamics of a Neotropical seed dispersal system. Global Ecology and Biogeography 30, 1129–1138.

[brv12809-bib-0224] Samuels, I. A. & Levey, D. J. (2005). Effects of gut passage on seed germination: do experiments answer the questions they ask? Functional Ecology 19, 365–368.

[brv12809-bib-0225] * Santos, N. N. D. (2017). *Estudo da dispersão do Butia catarinensis Noblick & Lorenzi no Parque Estadual da Serra do Tabuleiro*. Palhoça‐SC. BSc Dissertation: Universidade Federal de Santa Catarina, Florianópolis, Brazil.

[brv12809-bib-0226] * Sanín, M. J. & Galeano, G. (2011). A revision of the Andean wax palms, *Ceroxylon* (Arecaceae). Phytotaxa 34, 1–64.

[brv12809-bib-0227] Santos, A. A. & Ragusa‐Netto, J. (2014). Plant food resources exploited by blue‐and‐yellow macaws (*Ara ararauna*, Linnaeus 1758) at an urban area in Central Brazil. Brazilian Journal of Biology 74, 429–437. 10.1590/1519-6984.27312 25166327

[brv12809-bib-0228] Sazima, I. (2008). The parakeet *Brotogeris tirica* feeds on and disperses the fruits of the palm *Syagrus romanzoffiana* in southeastern Brazil. Biota Neotropica 8, 231–234.

[brv12809-bib-0229] Schleuning, M. , Fründ, J. & García, D. (2015). Predicting ecosystem functions from biodiversity and mutualistic networks: an extension of trait‐based concepts to plant–animal interactions. Ecography 38, 380–392.

[brv12809-bib-0230] Schleuning, M. , Neuschulz, E. L. , Albrecht, J. , Bender, I. M. , Bowler, D. E. , Dehling, D. M. , Fritz, S. A. , Hof, C. , Mueller, T. , Nowak, L. , Sorensen, M. C. , Böhning‐Gaese, K. & Kissling, W. D. (2020). Trait‐based assessments of climate‐change impacts on interacting species. Trends in Ecology & Evolution 35, 319–328. 31987640 10.1016/j.tree.2019.12.010

[brv12809-bib-0231] * Schlindwein, G. , Schlindwein, C. C. D. & Dillenburg, L. R. (2019). Seasonal cycle of seed dormancy controls the recruitment of *Butia odorata* (Arecaceae) seedlings in savanna‐like palm tree formations in southern Brazil. Austral Ecology 44, 1398–1409.

[brv12809-bib-0232] Schupp, E. W. , Jordano, P. & Gómez, J. M. (2010). Seed dispersal effectiveness revisited: a conceptual review. New Phytologist 188, 333–353. 20673283 10.1111/j.1469-8137.2010.03402.x

[brv12809-bib-0233] Schupp, E. W. , Zwolak, R. , Jones, L. R. , Snell, R. S. , Beckman, N. G. , Aslan, C. , Cavazos, B. R. , Effiom, E. , Fricke, E. C. , Montaño‐Centellas, F. , Poulsen, J. , Razafindratsima, O. H. , Sandor, M. E. & Shea, K. (2019). Intrinsic and extrinsic drivers of intraspecific variation in seed dispersal are diverse and pervasive. AoB Plants 11, plz067. 31857875 10.1093/aobpla/plz067 PMC6914678

[brv12809-bib-0234] Schurr, F. M. , Spiegel, O. , Steinitz, O. , Trakhtenbrot, A. , Tsoar, A. & Nathan, R. (2009). Long‐distance seed dispersal. Annual Plant Reviews 38, 204–237. 10.1016/j.tree.2008.08.003 18823680

[brv12809-bib-0235] Selwyn, M. , Garrote, P. J. , Castilla, A. R. & Fedriani, J. M. (2020). Interspecific interactions among functionally diverse frugivores and their outcomes for plant reproduction: a new approach based on camera‐trap data and tailored null models. PLoS One 15, e0240614. 33064761 10.1371/journal.pone.0240614 PMC7567357

[brv12809-bib-0236] Sezen, U. U. , Chazdon, R. L. & Holsinger, K. E. (2009). Proximity is not a proxy for parentage in an animal‐dispersed Neotropical canopy palm. Proceedings of the Royal Society B‐Biological Sciences 276, 2037–2044. 10.1098/rspb.2008.1793 PMC2677255 19324791

[brv12809-bib-0237] * Shiels, A. B. & Ramírez de Arellano, G. E. (2019). Habitat use and seed removal by invasive rats (*Rattus rattus*) in disturbed and undisturbed rain forest, Puerto Rico. Biotropica 51, 378–386.

[brv12809-bib-0238] Sica, Y. V. , Bravo, S. P. & Giombini, M. I. (2014). Spatial pattern of Pindo palm (*Syagrus romanzoffiana*) recruitment in Argentinian Atlantic Forest: the importance of tapir and effects of defaunation. Biotropica 46, 696–703.

[brv12809-bib-0239] Silva, F. R. , Begnini, R. M. , Lopes, C. B. & Castellani, T. T. (2011). Seed dispersal and predation in the palm *Syagrus romanzoffiana* on two islands with different faunal richness, southern Brazil. Studies on Neotropical Fauna and Environment 46, 163–171.

[brv12809-bib-0240] * Silva, J. Z. & Reis, M. S. (2019). Consumption of *Euterpe edulis* fruit by wildlife: implications for conservation and management of the southern Brazilian Atlantic Forest. Anais da Academia Brasileira de Ciências 91, e20180537. 30994770 10.1590/0001-3765201920180537

[brv12809-bib-0241] * Silva, M. G. & Tabarelli, M. (2001). Seed dispersal, plant recruitment and spatial distribution of *Bactris acanthocarpa* Martius (Arecaceae) in a remnant of Atlantic forest in Northeast Brazil. Acta Oecologica 22, 259–268.

[brv12809-bib-0242] * Silvestre, S. M. , da Rocha, P. A. , da Cunha, M. A. , Santana, J. P. & Ferrari, S. F. (2016). Diet and seed dispersal potential of the white‐lined bat, *Platyrrhinus lineatus* (E. Geoffroy, 1810), at a site in northeastern Brazil. Studies on Neotropical Fauna and Environment 51, 37–44.

[brv12809-bib-0243] Silvius, K. M. (2002). Spatio‐temporal patterns of palm endocarp use by three Amazonian forest mammals: granivory or ‘grubivory’? Journal of Tropical Ecology 18, 707–723.

[brv12809-bib-0244] Simmons, B. I. , Sutherland, W. J. , Dicks, L. V. , Albrecht, J. , Farwig, N. , García, D. , Jordano, P. & González‐Varo, J. P. (2018). Moving from frugivory to seed dispersal: incorporating the functional outcomes of interactions in plant–frugivore networks. Journal of Animal Ecology 87, 995–1007. 29603211 10.1111/1365-2656.12831 PMC6849527

[brv12809-bib-0245] Smythe, N. (1989). Seed survival in the palm *Astrocaryum standleyanum*: evidence for dependence upon its seed dispersers. Biotropica 21, 50–56.

[brv12809-bib-0246] Snell, R. S. , Beckman, N. G. , Fricke, E. , Loiselle, B. A. , Carvalho, C. S. , Jones, L. R. , Lichti, N. I. , Lustenhouwer, N. , Schreiber, S. J. , Strickland, C. , Sullivan, L. L. , Cavazos, B. R. , Giladi, I. , Hastings, A. , Holbrook, K. M. , et al. (2019). Consequences of intraspecific variation in seed dispersal for plant demography, communities, evolution and global change. AoB Plants 11, plz016. 31346404 10.1093/aobpla/plz016 PMC6644487

[brv12809-bib-0247] Sorensen, M. C. , Donoso, I. , Neuschulz, E. L. , Schleuning, M. & Mueller, T. (2020). Community‐wide seed dispersal distances peak at low levels of specialisation in size‐structured networks. Oikos 129, 1727–1738.

[brv12809-bib-0248] Sório, V. F. , Damasceno‐Junior, G. A. & Parolin, P. (2015). Dispersal of palm seeds (*Bactris glaucescens* Drude) by the fish *Piaractus mesopotamicus* in the Brazilian pantanal. Ecotropica 20, 15–12.

[brv12809-bib-0249] * Srbek‐Araujo, A. C. , Da Cunha, C. J. & Roper, J. J. (2017). Post‐dispersal seed predation by Atlantic Forest squirrels monitoring lowland tapir latrines. Tropical Ecology 58, 673–678.

[brv12809-bib-0250] Stevenson, P. R. , Cardona, L. M. , Acosta‐Rojas, D. C. , Henao‐Diaz, F. & Cárdenas, S. (2017). Diet of oilbirds (*Steatornis caripensis*) in Cueva de los Guácharos National Park (Colombia): temporal variation in fruit consumption, dispersal, and seed morphology. Ornitología Neotropical 28, 295–307.

[brv12809-bib-0251] Stevenson, P. R. , Cardona, L. , Cárdenas, S. & Link, A. (2021). Oilbirds disperse large seeds at longer distance than extinct megafauna. Scientific Reports 11, 1–8. 33431959 10.1038/s41598-020-79280-4 PMC7801487

[brv12809-bib-0252] Stevenson, P. R. & Link, A. (2010). Fruit preferences of *Ateles belzebuth* in Tinigua Park, northwestern Amazonia. International Journal of Primatology 31, 393–407.

[brv12809-bib-0253] * Stevenson, P. R. , Quiñones, M. J. & Ahumada, J. A. (2000). Influence of fruit availability on ecological overlap among four neotropical primates at Tinigua National Park, Colombia. Biotropica 32, 533–544.

[brv12809-bib-0254] Tella, J. L. , Baños‐Villalba, A. , Hernández‐Brito, D. , Rojas, A. , Pacífico, E. , Díaz‐Luque, J. A. , Carrete, M. , Blanco, G. & Hiraldo, F. (2015). Parrots as overlooked seed dispersers. Frontiers in Ecology and the Environment 13, 338–339.

[brv12809-bib-0255] Tella, J. L. , Hiraldo, F. , Pacifico, E. , Diaz‐Luque, J. A. , Denes, F. V. , Fontoura, F. M. , Guedes, N. & Blanco, G. (2020). Conserving the diversity of ecological interactions: the role of two threatened macaw species as legitimate dispersers of ‘megafaunal’ fruits. Diversity 12, 45.

[brv12809-bib-0256] ter Steege, H. , Pitman, N. C. , Sabatier, D. , Baraloto, C. , Salomão, R. P. , Guevara, J. E. , Phillips, O. L. , Castilho, C. V. , Magnusson, W. E. , Molino, J. F. , Monteagudo, A. , Vargas, P. N. , Montero, J. C. , Feldpausch, T. R. , Coronado, E. N. H. , et al. (2013). Hyperdominance in the Amazonian tree flora. Science 342, 6156. 10.1126/science.1243092 24136971

[brv12809-bib-0257] Thompson, J. N. (1988). Variation in interspecific interactions. Annual Review of Ecology and Systematics 19, 65–87.

[brv12809-bib-0258] Torres, D. A. , Castaño, J. H. & Carranza‐Quiceno, J. A. (2020). Global patterns in seed germination after ingestion by mammals. Mammal Review 50, 278–290.

[brv12809-bib-0259] Traveset, A. (1998). Effect of seed passage through vertebrate frugivores' guts on germination: a review. Perspectives in Plant Ecology, Evolution and Systematics 1, 151–190.

[brv12809-bib-0260] Traveset, A. & Verdú, M. (2002). A meta‐analysis of the effect of gut treatment on seed germination. In Seed Dispersal and Frugivory: Ecology, Evolution, and Conservation (eds D. J. Levey , M. Galetti and W. R. Silva ), pp. 339–350. CAB International, Wallingford.

[brv12809-bib-0261] Tucker Lima, J. M. , Caruso, N. M. , Clugston, J. & Kainer, K. A. (2018). Landscape change alters reproductive phenology and sex expression in *Attalea* palms (Arecaceae) of southwestern Amazonia. Plant Ecology 219, 1225–1245.

[brv12809-bib-0262] Udvardy, M. D. F. (1975). A Classification of the Biogeographical Provinces of the World. IUCN, Morges.

[brv12809-bib-0263] van der Hoek, Y. , Solas, S. A. & Peñuela, M. C. (2019). The palm *Mauritia flexuosa*, a keystone plant resource on multiple fronts. Biodiversity and Conservation 28, 539–551.

[brv12809-bib-0264] van Leeuwen, C. H. A. , Tella, J. L. & Green, A. J. (2020). Editorial: animal‐mediated dispersal in understudied systems. Frontiers in Ecology and Evolution 7, 508.

[brv12809-bib-0265] Vander Wall, S. B. (1990). Food Hoarding in Animals. University of Chicago Press, Chicago.

[brv12809-bib-0266] Vander Wall, S. B. & Beck, M. J. (2012). A comparison of frugivory and scatter‐hoarding seed‐dispersal syndromes. The Botanical Review 78, 10–31.

[brv12809-bib-0267] Verdú, M. & Traveset, A. (2004). Bridging meta‐analysis and the comparative method: a test of seed size effect on germination after frugivores' gut passage. Oecologia 138, 414–418. 14689293 10.1007/s00442-003-1448-4

[brv12809-bib-0268] Villalobos, M. P. & Bagno, M. A. (2012). Avian frugivores feeding on *Mauritia flexuosa* (Arecaceae) fruits in Central Brazil. Revista Brasileira de Ornitologia 20, 26–29.

[brv12809-bib-0269] Villar, N. , Paz, C. , Zipparro, V. , Nazareth, S. , Bulascoschi, L. , Bakker, E. S. & Galetti, M. (2020). Frugivory underpins the nitrogen cycle. Functional Ecology 35, 357–368.

[brv12809-bib-0270] * Visser, M. D. , Muller‐Landau, H. C. , Wright, S. J. , Rutten, G. & Jansen, P. A. (2011). Tri‐trophic interactions affect density dependence of seed fate in a tropical forest palm. Ecology Letters 14, 1093–1100. 21899693 10.1111/j.1461-0248.2011.01677.x

[brv12809-bib-0271] Wang, B. C. & Smith, T. B. (2002). Closing the seed dispersal loop. Trends in Ecology & Evolution 17, 379–386.

[brv12809-bib-0272] * Wehncke, E. V. , López‐Medellín, X. & Ezcurra, E. (2010). Blue fan palm distribution and seed removal patterns in three desert oases of northern Baja California, Mexico. Plant Ecology 208, 1–20.

[brv12809-bib-0273] * Wehncke, E. V. , Medellin, X. L. & Ezcurra, E. (2009). Patterns of frugivory, seed dispersal and predation of blue fan palms (*Brahea armata*) in oases of northern Baja California. Journal of Arid Environments 73, 773–783.

[brv12809-bib-0274] * Wright, S. J. & Duber, H. C. (2001). Poachers and forest fragmentation alter seed dispersal, seed survival, and seedling recruitment in the palm *Attalea butyraceae*, with implications for tropical tree diversity. Biotropica 33, 583–595.

[brv12809-bib-0275] * Wright, S. J. , Zeballos, H. , Domínguez, I. , Gallardo, M. M. , Moreno, M. C. & Ibáñez, R. (2000). Poachers alter mammal abundance, seed dispersal, and seed predation in a Neotropical forest. Conservation Biology 14, 227–239.

[brv12809-bib-0276] Zona, S. & Henderson, A. (1989). A review of animal‐mediated seed dispersal of palms. Selbyana 11, 6–21.

[brv12809-bib-0277] * Zona, S. (1990). A monograph of *Sabal* (Arecaceae: Coryphoideae). Aliso 12, 583–666.

[brv12809-bib-0278] Zorzi, B. T. (2009). Frugivoria por *Tapirus terrestris* em três regiões do Pantanal, Brasil. MSc Dissertation: Universidade Federal do Mato Grosso do Sul, Campo Grande, Brazil.

[brv12809-bib-0279] * Zucaratto, R. & Pires, A. S. (2015). Local extinction of an important seed disperser does not modify the spatial distribution of the endemic palm *Astrocaryum aculeatissimum* (Schott) Burret (Arecaceae). Acta Botanica Brasilica 29, 244–250.

[brv12809-bib-0280] Zwolak, R. & Crone, E. E. (2012). Quantifying the outcome of plant–granivore interactions. Oikos 121, 20–27.

[brv12809-bib-0281] Zwolak, R. (2018). How intraspecific variation in seed‐dispersing animals matters for plants. Biological Reviews, 93, 897–913. 29024277 10.1111/brv.12377

