# Supplementary material for: The mutualism–antagonism continuum in Neotropical palm–frugivore interactions: from interaction outcomes to ecosystem dynamics
Source: Biol Rev Camb Philos Soc. 2021 Nov 1;97(2):527–53. doi: 10.1111/brv.12809 (PMC9297963; doi:10.1111/brv.12809)
Supplement: Supplementary file 1 — Appendix S1. Combination of search terms for literature compilation in the Web of Science. Appendix S2. Interaction types and criteria to classify interactions into positive, negative or dual outcomes based on quantitative and qualitative evidence from published articles on palm–frugivore interactions. Appendix S3. List of palm species by tribe and genera recorded in interactions with frugivores in the data set with types of feeding interactions. Appendix S4. Number of animal species and interaction records in different types of feeding interactions (fruit‐, pulp‐, and seed‐eating), summarized for each family, order and class. Appendix S5. The role of digestive‐processing types for fruits in interactions between fruit‐eaters and palms. Appendix S6. Dispersal distances recorded for interactions between vertebrate frugivores and Neotropical palm species. Appendix S7. The role of fruit‐handling ability in interactions between pulp‐eaters and palms. Appendix S8. The role of handling ability and seed‐caching behaviour in interactions between seed‐eaters and palms. Appendix S9. Fruit size of palm species recorded in interactions with frugivores, according to parts of fruits consumed by animals. [file BRV-97-527-s001.pdf]

SUPPORTING INFORMATION

The mutualism–antagonism continuum in Neotropical palm–frugivore interactions: from interaction outcomes to ecosystem dynamics

Caroline Marques Dracxler* & W. Daniel Kissling

Affiliation

Institute for Biodiversity and Ecosystem Dynamics (IBED), University of Amsterdam, P.O. Box 94240, 1090 GE Amsterdam, The Netherlands

*Author for correspondence (E-mail: carolinemdsbio@yahoo.com.br)

Appendix S1. Combination of search terms for literature compilation in the *Web of Science*.

(seed dispers* or frugivor* or seed predat* or fruit remov* or granivor* or interaction* or dispers* or predat* or remov*) AND (fruit* or seed* or endocarp) AND (americ* or neotropic* or anguilla or "antigua and Barbuda" or aruba or bahamas or barbados or "british virgin islands" or "cayman islands" or cuba or dominica or "dominican republic" or grenada or guadeloupe or haiti or jamaica or martinique or montserrat or antilles or "puerto rico" or "saint kitts and nevis" or "saint lucia" or "saint martin" or "saint vincent and the grenadines" or "saint-barthélemy" or "trinidad and tobago" or "turks and caicos islands" or "united states virgin islands" or belize or "costa rica" or "el salvador" or guatemala or honduras or mexico or nicaragua or panama or bermuda or argentina or bolivia or brazil or chile or colombia or ecuador or "falkland islands" or malvinas or "french guiana" or "guyane francaise" or guyana or paraguay or peru or suriname or uruguay or venezuela) AND (palm* or arecaceae or trithrinax or chelyocarpus or cryosophila or itaya or schippia or thrinax or coccothrinax or zombia or rhapidophyllum or colpothrinax or acoelorraphe or serenoa or brahea or copernicia or washingtonia or sabal or raphia or mauritia or mauritiella or lepidocaryum or pseudophoenix or ceroxylon or juania or gaussia or synechanthus or chamaedorea or wendlandiella or dictyocaryum or iriartella or iriartea or socratea or wettinia or manicaria or leopoldinia or reinhardtia or euterpe or prestoea or neonicholsonia or oenocarpus or hyospathe or roystonea or butia or jubaea or cocos or syagrus or lytocaryum or parajubaea or allagoptera or polyandrococos or attalea or barcella or elaeis or acrocomia or gastrococos or aiphanes or bactris or desmoncus or astrocaryum or pholidostachys or welfia or calyptronoma or calyptrogyne or asterogyne or geonoma or phytelephas or ammandra or aphandra)

Appendix S2. Interaction types and criteria to classify interactions into positive, negative or dual outcomes based on quantitative and qualitative evidence from published articles on palm–frugivore interactions. Interaction outcomes of each pairwise interaction record were assessed separately for each of three interaction types (fruit-eating, seed-eating and pulp-eating).

Interaction type	Outcome	Criteria used to classify interaction outcomes
Fruit-eating	Positive	gut-passed seeds are viable or germinate; endozoochory is observed; seeds are dispersed or animal is (main/effective) disperser; animal contributes to seedling establishment
	Negative	seeds are predated; feeds on unripe fruits; gut-passed seeds are not viable; only fragments of seeds found in stomach or feces; seeds do not germinate or establish into seedlings
	Dual	seeds are both predated and dispersed; animal acts as seed disperser and predator
Pulp-eating	Positive	intact, defleshed seeds are dropped, discarded or spat out after pulp removal; removes only pulp without damaging the seed; animal is a (main/effective) disperser or seeds are dispersed
	Negative	seeds are damaged; seeds do not survive pulp consumption; animal is a seed predator
	Dual	seeds are both predated and dispersed; animal acts as seed disperser and predator
Seed-eating	Positive	seeds are dropped intact; seeds are dispersed; seeds are cached; high proportion of seeds cached rather than predated; caches are not recovered; dispersed or cached seeds germinate or establish as seedlings; ectozoochory is observed; animal is a (main/effective) disperser
	Negative	seeds are predated; most cached seeds are recovered and predated; cached seeds do not germinate; feeds on seeds of unripe fruits; animal is a seed predator
	Dual	seeds are both predated and dispersed (or cached); seeds are removed; seeds are occasionally dispersed; animal is a seed disperser and predator

Appendix S3. List of palm species by tribe and genera recorded in interactions with frugivores in the dataset with types of feeding interactions. * indicates non-native palms included in the interaction dataset.

Tribe Areceae

Archontophoenix
*A. cunninghamiana**

Dypsis
*D. lutescens**

Tribe Ceroxyleae

Ceroxylon
C. alpinum
C. ceriferum
C. quindiuense

Tribe Chamaedoreeae

Chamaedorea
C. linearis
C. tepejilote

Synechanthus
S. warscewiczianus

Tribe Cocoseae

Acrocomia
A. aculeata
A. totai

Aiphanes
A. horrida

Allagoptera
A. arenaria
A. campestris
A. caudescens
A. leucocalyx

Astrocaryum
A. aculeatissimum
A. aculeatum
A. alatum
A. chambira
A. gratum
A. jauari
A. mexicanum
A. murumuru
A. paramaca
A. sciophilum
A. standleyanum
A. tucuma
A. vulgare

Attalea
A. attaleoides
A. barreirensis
A. butyracea
A. cohune
A. dubia
A. eichleri
A. funifera
A. geraensis
A. humilis
A. maripa
A. oleifera
A. phalerata
A. princeps
A. rostrata
A. speciosa

Bactris
B. acanthocarpa
B. barronis
B. ferruginea
B. gasipaes
B. glaucescens
B. major
B. maraja
B. mexicana
B. setosa

Barcella
B. odora

Butia
B. catarinensis
B. eriospatha
B. odorata
B. yatay

Cocos
*C. nucifera**

Elaeis
*E. guineensis**

Syagrus
S. flexuosa
S. loefgrenii
S. oleracea
S. pseudococos
S. romanzoffiana
S. ruschiana
S. sancona

Tribe Cryosophileae

Coccothrinax
C. barbadensis

Cryosophila
C. guagara

Leucothrinax
L. morrisii

Tribe Euterpeae

Euterpe
E. edulis
E. oleracea
E. precatoria

Oenocarpus
O. bacaba
O. bataua
O. mapora

Prestoea
P. acuminata

Tribe Geonomateae

Geonoma
G. pauciflora
G. schottiana
G. undata

Welfia
W. regia

Tribe Irarteeae

Dictyocaryum
D. lamarckianum

Iriartea
I. deltoidea

Iriartella
I. setigera

Socratea
S. exorrhiza

Wettinia
W. fascicularis
W. kalbreyeri

Tribe Lepidocaryeae

Mauritia
M. flexuosa

Mauritiella
M. aculeata

Raphia
R. taedigera

Tribe Phytelepheae

Phytelephas
P. aequatorialis
P. macrocarpa
P. seemannii

Tribe Roystoneeae

Roystonea
R. oleracea

Tribe Sabaleae

Sabal
S. causiarum
S. etonia
S. mexicana
S. palmetto
S. yapa

Tribe Trachycarpeae

Brahea
B. armata
B. brandegeei

Copernicia
C. alba
C. prunifera
C. tectorum

Livistona
*L. chinensis**

Serenoa
S. repens

Washingtonia
W. robusta

Appendix S4. Number of animal species and interaction records in different types of feeding interactions (fruit-, pulp, and seed-eating), summarized for each family, order and class.

Frugivore class and order	Frugivore family	Fruit-eating		Pulp-eating		Seed-eating	
		# species	# records	# species	# records	# species	# records
**Aves**							
Caprimulgiformes	Steatornithidae	1	12				
Charadriiformes	Laridae	1	1				
Columbiformes	Columbidae	4	4				
Coraciiformes	Momotidae	2	5				
Falconiformes	Falconidae			1	1		
Galliformes	Cracidae	10	28				
	Odontophoridae	3	3				
Gruiformes	Psophiidae	1	1				
Passeriformes	Cardinalidae	6	6	1	1		
	Corvidae	8	12	3	3		
	Cotingidae	6	21				
	Formicariidae	1	1				
	Fringillidae	4	4	2	3		
	Icteridae	3	3	1	1		
	Parulidae	1	1	1	1		
	Passerellidae	3	3				
	Pipridae	1	1				
	Ptiliogonatidae	1	1				
	Thraupidae	1	1	12	12		
	Tityridae	1	2				
	Troglodytidae	1	1				
	Turdidae	9	41	2	2		
	Tyrannidae	4	7				
	Vireonidae			1	1		
Piciformes	Picidae	4	4				
	Ramphastidae	14	55				
Psittaciformes	Psittacidae	11	14	8	17	5	17
Rheiformes	Rheidae	1	8				
Tinamiformes	Tinamidae	3	3				
Trogoniformes	Trogonidae	5	8				
**Mammalia**							
Carnivora	Canidae	6	14				
	Felidae	2	3				
	Mustelidae	1	1				
	Procyonidae	4	14	1	2	1	1
	Ursidae	1	3				
Cetartiodactyla	Bovidae	3	9				
	Cervidae	4	7				
	Suidae	1	7				
	Tayassuidae	2	58	2	24	2	12

Chiroptera	Phyllostomidae	3	3	1	1		
Cingulata	Chlamyphoridae			1	1		
	Dasypodidae	1	1	1	1		
Didelphimorphia	Didelphidae	3	4	4	7	1	1
Perissodactyla	Equidae	1	1				
	Tapiridae	2	65	1	1		
Primates	Aotidae	1	1				
	Atelidae	4	9	3	3		
	Callitrichidae	1	1				
	Cebidae	4	9	6	12	1	2
	Pitheciidae	1	1			1	2
Rodentia	Cricetidae	5	5	3	3	15	24
	Cuniculidae	1	3	1	4	1	16
	Dasyproctidae	2	8	3	5	9	102
	Echimyidae	1	1	1	2	9	41
	Erethizontidae					1	1
	Heteromyidae					11	22
	Muridae					1	1
	Sciuridae	10	41	4	7	15	141
**Osteichthyes**							
Characiformes	Characidae	2	3				
	Serrasalmidae	3	5				
Siluriformes	Doradidae	2	4				
	Heptapteridae	1	1				
	Pimelodidae	2	2				
**Reptilia**							
Squamata	Boidae	1	1				
	Teiidae	1	1				
Testudines	Chelidae	1	5				
	Emydidae	1	2				
	Testudinidae	1	1				
	**TOTAL**	**189**	**545**	**64**	**115**	**73**	**383**

Appendix S5. The role of digestive-processing types for fruits in interactions between fruit-eaters and palms. (A) Number of recorded interactions between fruit-eaters and palms separated by digestive-processing type (defecation, regurgitation, or both) and taxonomic classes of animals, including birds (Aves), mammals (Mammalia), reptiles (Reptilia) and bony fish (Osteichthyes). (B) Proportion of interaction records per digestive-processing type resulting in positive (blue), negative (red) and dual outcomes (grey).

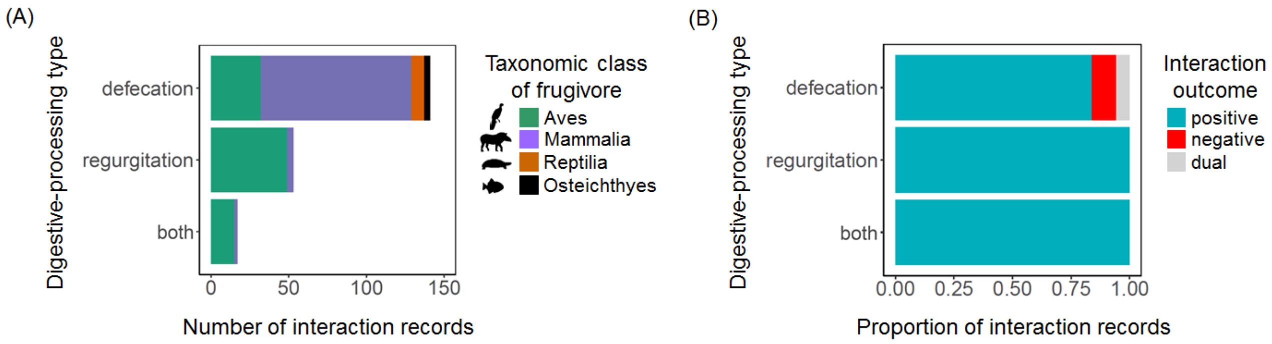

Appendix S6. Examples of dispersal distances recorded for interactions between vertebrate frugivores and Neotropical palm species. Type of feeding interactions that involves consumption of fruits, pulp or seeds of palms are shown (*shows cases for which type of feeding was assumed from the literature). The source shows articles from which data on dispersal distances was compiled, and citation indicates original references cited in the sources (e.g. when sources provided a compilation from other articles).

**Source**	**Frugivore class**	**Frugivore species**	**Palm species**	**Type of feeding interaction**	**Minimum distance (m)**	**Maximum distance (m)**	**Mean distance (m)**	**Citation**
Hirsch *et al.* (2012*b*)	Mammalia	*Dasyprocta punctata*	*Astrocaryum standleyanum*	seed	0.1	241.3	16.8	
Villalobos & Bagno (2012)	Aves	*Orthopsittaca manilatus*	*Mauritia flexuosa*	pulp		500		
Villalobos & Bagno (2012)	Aves	*Cyanocorax cristatellus*	*Mauritia flexuosa*	pulp		300		
Tella *et al.* (2020)	Aves	*Anodorhynchus leari*	*Syagrus coronata*	seed	3	250	248.9	
Tella *et al.* (2020)	Aves	*Anodorhynchus hyacinthinus*	*Acrocomia totai*	seed	1	400	162.4	
Tella *et al.* (2020)	Aves	*Anodorhynchus hyacinthinus*	*Attalea barreirensis*	seed	3	1620	218.9	
Tella *et al.* (2020)	Aves	*Anodorhynchus hyacinthinus*	*Attalea eichleri*	seed	1	223	17.1	
Tella *et al.* (2020)	Aves	*Anodorhynchus hyacinthinus*	*Attalea phalerata*	seed	4	1011	453.6	
Tella *et al.* (2020)	Aves	*Anodorhynchus hyacinthinus*	*Mauritia flexuosa*	seed	40	234	216.4	
Mittelman *et al.* (2021)	Mammalia	*Dasyprocta leporina*	*Astrocaryum aculeatissimum*	seed			5.9	Donatti *et al.* (2009)
Mittelman *et al.* (2021)	Mammalia	*Dasyprocta leporina*	*Astrocaryum aculeatissimum*	seed		48.7	7.8	Pires & Galetti (2012)
Mittelman *et al.* (2021)	Mammalia	*Dasyprocta leporina*	*Astrocaryum aculeatissimum*	seed		30	13.8	Zucaratto & Pires (2015)
Mittelman *et al.* (2021)	Mammalia	*Dasyprocta leporina*	*Astrocaryum aculeatum*	seed		40	6.4	Jorge & Howe (2009)
Mittelman *et al.* (2021)	Mammalia	*Dasyprocta punctata*	*Astrocaryum alatum*	seed		24	4.1	Kuprewicz (2013)
Mittelman *et al.* (2021)	Mammalia	*Dasyprocta punctata*	*Astrocaryum alatum*	seed		22	3.6	Kuprewicz (2015)
Mittelman *et al.* (2021)	Mammalia	*Dasyprocta fuliginosa*	*Astrocaryum chambira*	seed			8.6	Ramírez *et al.* (2009)
Mittelman *et al.* (2021)	Mammalia	*Dasyprocta punctata*	*Astrocaryum standleyanum*	seed		19	7	Gálvez *et al.* (2009)
Mittelman *et al.* (2021)	Mammalia	*Dasyprocta punctata*	*Astrocaryum standleyanum*	seed		280	55	Hirsch *et al.* (2012*a*)
Mittelman *et al.* (2021)	Mammalia	*Dasyprocta punctata*	*Astrocaryum standleyanum*	seed		280	54.9	Jansen *et al.* (2012)
Mittelman *et al.* (2021)	Mammalia	*Dasyprocta azarae*	*Attalea geraensis*	seed		30	6.5	de Almeida & Galetti (2007)
Mittelman *et al.* (2021)	Mammalia	*Dasyprocta prymnolopha*	*Attalea oleifera*	seed		8	5	Pimentel & Tabarelli (2004)
Mittelman *et al.* (2021)	Mammalia	*Dasyprocta azarae*	*Attalea phalerata*	seed		20.5	5.8	de Arruda Nascimento *et al.* (2004)
Mittelman *et al.* (2021)	Mammalia	*Dasyprocta punctata*	*Attalea rostrata*	seed			4	Forget *et al.* (1994)
Mittelman *et al.* (2021)	Mammalia	*Dasyprocta prymnolopha*	*Bactris acanthocarpa*	seed		5.3	4	Silva & Tabarelli (2001)
Mittelman *et al.* (2021)	Mammalia	*Dasyprocta punctata*	*Iriartea deltoidea*	seed		22	6.5	Kuprewicz (2013)

Mittelman *et al.* (2021)	Mammalia	*Dasyprocta punctata*	*Mauritia flexuosa*	seed	12.63		Mendieta-Aguilar *et al.* (2015)
Mittelman *et al.* (2021)	Mammalia	*Dasyprocta fuliginosa*	*Oenocarpus bataua*	seed	53	3.39	Franco-Quimbay & Rojas-Robles (2015)
Mittelman *et al.* (2021)	Mammalia	*Dasyprocta punctata*	*Oenocarpus bataua*	seed	53	3.39	Franco-Quimbay & Rojas-Robles (2015)
Mittelman *et al.* (2021)	Mammalia	*Dasyprocta punctata*	*Oenocarpus bataua*	seed	53	3.1	Rojas-Robles *et al.* (2012)
Mittelman *et al.* (2021)	Mammalia	*Dasyprocta punctata*	*Socratea exorrhiza*	seed	2	2	Kuprewicz (2013)
Blanco *et al.* (2019)	Mammalia	*Tolypeutes tricinctus*	*Attalea barreirensis*	pulp*		0.8	
Blanco *et al.* (2019)	Mammalia	*Sciurus ignitus*	*Attalea princeps*	seed*		25	
Blanco *et al.* (2019)	Mammalia	*Holochilus sciureus*	*Attalea speciosa*	pulp*		2	
Blanco *et al.* (2019)	Mammalia	*Dasyprocta prymnolopha*	*Mauritia flexuosa*	seed*		2.5	
Blanco *et al.* (2019)	Mammalia	*Dasyprocta punctata*	*Attalea speciosa*	seed*		4	
Blanco *et al.* (2019)	Mammalia	*Cuniculus paca*	*Attalea speciosa*	pulp*		4	
Blanco *et al.* (2019)	Mammalia	*Cuniculus paca*	*Mauritia flexuosa*	pulp*		5.3	
Blanco *et al.* (2019)	Mammalia	*Artibeus* sp.	*Mauritia flexuosa*	pulp*		74	
Blanco *et al.* (2019)	Mammalia	*Chrysocyon brachyurus*	*Attalea barreirensis*	pulp*		15	
Blanco *et al.* (2019)	Mammalia	*Chrysocyon brachyurus*	*Mauritia flexuosa*	pulp*		61.5	
Blanco *et al.* (2019)	Aves	*Caracara plancus*	*Attalea barreirensis*	pulp*		18.5	
Blanco *et al.* (2019)	Aves	*Anodorhynchus hyacinthinus*	*Attalea eichleri*	seed		15.7	
Blanco *et al.* (2019)	Aves	*Anodorhynchus hyacinthinus*	*Attalea barreirensis*	seed		66	
Blanco *et al.* (2019)	Aves	*Anodorhynchus hyacinthinus*	*Mauritia flexuosa*	seed		156.3	
Blanco *et al.* (2019)	Aves	*Anodorhynchus hyacinthinus*	*Acrocomia totai*	seed		31.5	
Blanco *et al.* (2019)	Aves	*Ara ararauna*	*Attalea speciosa*	pulp*		24.5	
Blanco *et al.* (2019)	Aves	*Ara ararauna*	*Attalea princeps*	pulp		20.6	
Blanco *et al.* (2019)	Aves	*Ara ararauna*	*Mauritia flexuosa*	pulp		205.8	
Blanco *et al.* (2019)	Aves	*Ara ararauna*	*Acrocomia totai*	pulp*		60	
Blanco *et al.* (2019)	Aves	*Ara glaucogularis*	*Attalea princeps*	pulp		69.1	
Blanco *et al.* (2019)	Aves	*Ara glaucogularis*	*Acrocomia totai*	pulp*		7	
Blanco *et al.* (2019)	Aves	*Ara chloropterus*	*Acrocomia totai*	pulp*		61.8	
Blanco *et al.* (2019)	Aves	*Ara chloropterus*	*Mauritia flexuosa*	pulp*		180.3	
Blanco *et al.* (2019)	Aves	*Ara severus*	*Attalea princeps*	pulp		38.5	
Blanco *et al.* (2019)	Aves	*Ara severus*	*Acrocomia totai*	pulp		90.3	
Blanco *et al.* (2019)	Aves	*Orthopsittaca manilatus*	*Mauritia flexuosa*	pulp		172.8	
Blanco *et al.* (2019)	Aves	*Thectocercus acuticaudatus*	*Acrocomia totai*	pulp*		50	

Blanco *et al.* (2019)	Aves	*Brotogeris chiriri*	*Acrocomia totai*	pulp*		5	
Blanco *et al.* (2019)	Aves	*Amazona aestiva*	*Attalea princeps*	pulp*		8	
Blanco *et al.* (2019)	Aves	*Amazona aestiva*	*Mauritia flexuosa*	pulp		51	
Blanco *et al.* (2019)	Aves	*Amazona aestiva*	*Acrocomia totai*	pulp*		47.5	
Blanco *et al.* (2019)	Aves	*Cyanocorax cyanomelas*	*Attalea princeps*	pulp		17.5	
Stevenson *et al.* (2021)	Aves	*Steatornis caripensis*	*Oenocarpus bataua*	fruit	43200		
Stevenson *et al.* (2021)	Mammalia	*Ateles chamek*	*Socratea exorrhiza*	fruit*	1500	245	Russo *et al.* (2006)
Stevenson *et al.* (2021)	Reptilia	*Chelonoidis denticulata*	*Astrocaryum murumuru*	fruit*	1100	226	Jerozolimski *et al.* (2009)
Stevenson *et al.* (2021)	Osteichthyes	*Colossoma macropomum*	*Astrocaryum jauari*	fruit	5495	445	Anderson *et al.* (2011)

Appendix S7. The role of fruit-handling ability in interactions between pulp-eaters and palms. (A) Number of interaction records according to handling ability separated for birds (Aves) and mammals (Mammalia). (B) Proportion of interaction records for each handling ability category classified according to positive (blue), negative (red) and dual outcomes (grey).

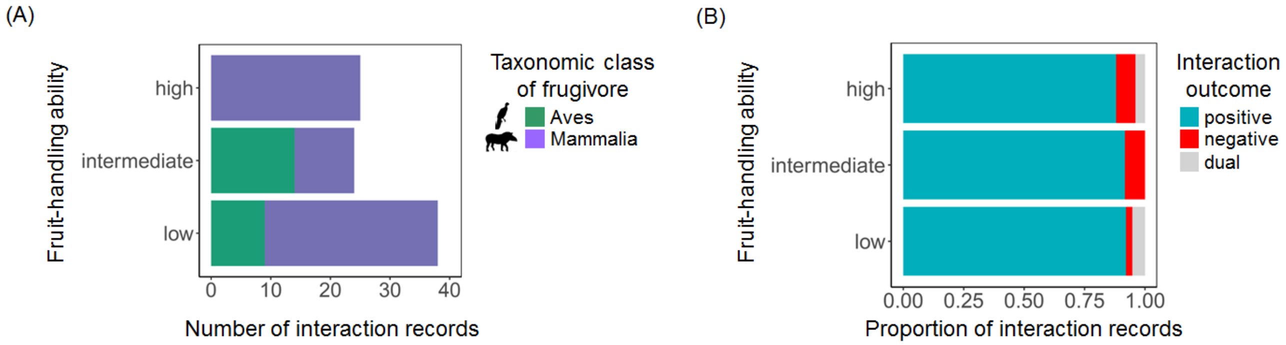

Appendix S8. The role of fruit-handling ability and seed-caching behaviour in interactions between seed-eaters and palms. (A) Number of recorded interactions for seed-eaters per category of fruit-handling ability (high, intermediate, low), separated by taxonomic class (Aves, Mammalia). (B) Proportion of interaction records according to fruit-handling ability in relation to positive (blue), negative (red) and dual interaction outcomes (grey). (C) Number of recorded interactions in relation to seed-caching behaviour (no caching, caching), separated for birds and mammals. (D) Proportion of interaction records in relation to seed-caching behaviour resulting in positive (blue), negative (red) and dual outcomes (grey).

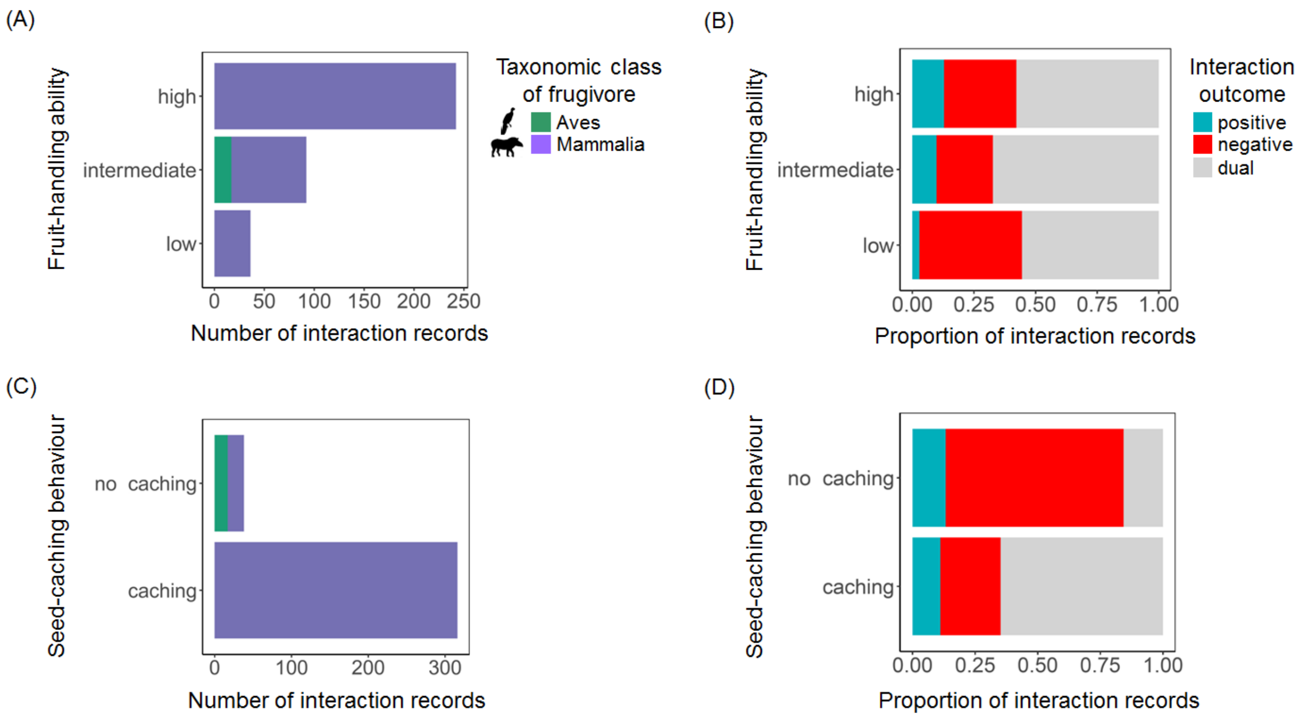

Appendix S9. Fruit size of palm species recorded in interactions with frugivores, according to parts of fruits consumed by animals (f = fruit; p = pulp; s = seed). Fruit size measurements (average fruit width in cm) were extracted from PalmTraits 1.0 (Kissling *et al.*, 2019) for all palm species (*N* = 106 spp.) that are captured in the dataset of palm-frugivore interaction records.

Palm species	Fruit width (cm)	Part of fruit consumed
Acrocomia aculeata	4.6	f, p
Acrocomia totai	3	f, p, s
Aiphanes horrida	2	f
Allagoptera arenaria	1.2	f, s
Allagoptera campestris	0.6	s
Allagoptera caudescens	3.3	f, s
Allagoptera leucocalyx	1.75	f
Archontophoenix cunninghamiana	1.1	f
Astrocaryum aculeatissimum	3.5	f, s
Astrocaryum aculeatum	4	f, p, s
Astrocaryum alatum	1.8	p, s
Astrocaryum chambira	4.8	f, p, s
Astrocaryum gratum	3.5	s
Astrocaryum jauari	2.4	f
Astrocaryum mexicanum	1.8	f, s
Astrocaryum murumuru	4.1	f, p, s
Astrocaryum paramaca	2.3	f, s
Astrocaryum sciophilum	3.3	f, s
Astrocaryum standleyanum	3.1	f, p, s
Astrocaryum tucuma	4	f
Astrocaryum vulgare	3.2	f
Attalea attaleoides	2.3	s
Attalea barreirensis	3.8	s
Attalea butyracea	3.8	f, p, s
Attalea cohune	3.9	s
Attalea dubia	3	s
Attalea eichleri	4.8	s
Attalea funifera	6	p, s
Attalea geraensis	3.8	s
Attalea humilis	5.3	s
Attalea maripa	2.8	f, p, s
Attalea oleifera	5	f, p, s
Attalea phalerata	4	f, p, s
Attalea princeps	3.4	f, p, s
Attalea rostrata	2.5	f, p, s
Attalea speciosa	6.8	f, p
Bactris acanthocarpa	1.4	f, s
Bactris barronis	1.3	s
Bactris ferruginea	1.7	f

Bactris gasipaes	4	f
Bactris glaucescens	1.6	f
Bactris major	2.4	f, s
Bactris maraja	1.5	s
Bactris mexicana	1.1	s
Bactris setosa	1.8	f, s
Barcella odora	2.5	f
Brahea armata	1.6	f, s
Brahea brandegeei	1.75	f
Butia catarinensis	1.2	f, p
Butia eriospatha	1.8	f
Butia odorata	1.9	f
Butia yatay	2.7	f
Ceroxylon alpinum	1.8	f
Ceroxylon ceriferum	1.7	f
Ceroxylon quindiuense	1.8	f
Chamaedorea linearis	1.7	f
Chamaedorea tepejilote	0.8	s
Coccothrinax barbadensis	0.9	f, s
Cocos nucifera	14	f, s
Copernicia alba	1.3	f
Copernicia prunifera	2.2	f
Copernicia tectorum	1.8	p
Cryosophila guagara	1.7	s
Dictyocaryum lamarckianum	2.7	f
Dypsis lutescens	0.9	f
Elaeis guineensis	2.5	f, p, s
Euterpe edulis	1.2	f, p, s
Euterpe oleracea	1.5	f, s
Euterpe precatoria	1.1	f
Geonoma pauciflora	0.8	f, s
Geonoma schottiana	0.8	f
Geonoma undata	0.7	f, s
Iriartea deltoidea	2.5	f, s
Iriartella setigera	0.9	f
Leucothrinax morrisii	0.5	f
Livistona chinensis	2.8	f, p, s
Mauritia flexuosa	5	f, p, s
Mauritiella aculeata	3.8	f
Oenocarpus bacaba	1.4	f
Oenocarpus bataua	2.4	f, p, s
Oenocarpus mapora	2	f, s
Phytelephas aequatorialis	8	s
Phytelephas macrocarpa	15.5	p, s
Phytelephas seemannii	14	s

Prestoea acuminata	1.4	f, s
Raphia taedigera	3.5	f
Roystonea oleracea	0.9	p
Sabal causiarum	0.9	f
Sabal etonia	1.1	f
Sabal Mexicana	1.4	f
Sabal palmetto	1.1	f
Sabal yapa	1.3	f
Serenoa repens	1.6	f
Socratea exorrhiza	1.8	f, s
Syagrus flexuosa	2.3	s
Syagrus loefgrenii	1.3	s
Syagrus oleracea	2.4	f, p, s
Syagrus pseudococos	3.9	f, s
Syagrus romanzoffiana	1.5	f, p, s
Syagrus ruschiana	2	f, s
Syagrus sancona	1.9	f, p
Synechanthus warscewiczianus	0.9	s
Washingtonia robusta	1	f
Welfia regia	1.5	f, s
Wettinia fascicularis	5	s
Wettinia kalbreyeri	5	s
